# Pathway-Focused PCR Array Profiling of Enriched Populations of Laser Capture Microdissected Hippocampal Cells after Traumatic Brain Injury

**DOI:** 10.1371/journal.pone.0127287

**Published:** 2015-05-27

**Authors:** Deborah R. Boone, Maria-Adelaide Micci, Isabella G. Taglialatela, Judy L. Hellmich, Harris A. Weisz, Min Bi, Donald S. Prough, Douglas S. DeWitt, Helen L. Hellmich

**Affiliations:** Department of Anesthesiology, The University of Texas Medical Branch, 301 University Boulevard, Galveston, Texas 77555–0830, United States of America; Universidade Federal do ABC, BRAZIL

## Abstract

Cognitive deficits in survivors of traumatic brain injury (TBI) are associated with irreversible neurodegeneration in brain regions such as the hippocampus. Comparative gene expression analysis of dying and surviving neurons could provide insight into potential therapeutic targets. We used two pathway-specific PCR arrays (RT2 Profiler Apoptosis and Neurotrophins & Receptors PCR arrays) to identify and validate TBI-induced gene expression in dying (Fluoro-Jade-positive) or surviving (Fluoro-Jade- negative) pyramidal neurons obtained by laser capture microdissection (LCM). In the Apoptosis PCR array, dying neurons showed significant increases in expression of genes associated with cell death, inflammation, and endoplasmic reticulum (ER) stress compared with adjacent, surviving neurons. Pro-survival genes with pleiotropic functions were also significantly increased in dying neurons compared to surviving neurons, suggesting that even irreversibly injured neurons are able to mount a protective response. In the Neurotrophins & Receptors PCR array, which consists of genes that are normally expected to be expressed in both groups of hippocampal neurons, only a few genes were expressed at significantly different levels between dying and surviving neurons. Immunohistochemical analysis of selected, differentially expressed proteins supported the gene expression data. This is the first demonstration of pathway-focused PCR array profiling of identified populations of dying and surviving neurons in the brain after TBI. Combining precise laser microdissection of identifiable cells with pathway-focused PCR array analysis is a practical, low-cost alternative to microarrays that provided insight into neuroprotective signals that could be therapeutically targeted to ameliorate TBI-induced neurodegeneration.

## Introduction

Presently, there are no approved treatments that can be administered after traumatic brain injury (TBI) to mitigate the progression of brain damage and improve functional outcome. During the past several decades, thousands of gene expression studies have provided much insight into the pathogenesis of TBI, and led to therapeutic strategies to inhibit genes and cellular pathways associated with cell death but these have not translated into effective treatments [1–4]; to date, hundreds of clinical trials of brain injury have failed to advance past Phase 2 trials [5,6]. This failure is reflected in the clinical management of TBI patients; once they are stabilized, there is little other than supportive care that can be done to limit the cascades of neuronal inflammation, oxidative stress and cell death [7–9]. Therefore, it is imperative to continue exploring the molecular underpinnings of TBI, particularly, the injury-induced changes in the molecular signals that are critically important for survival, regeneration and recovery of the injured brain.

In a recent, comparative, genome-wide gene expression analysis of dying and surviving neurons obtained by laser capture microdissection (LCM), we gained several insights into mechanisms of cell survival in the hippocampus of rats subjected to TBI [10]. Specifically, we showed evidence of pre- and post-injury stochasticity in gene expression in both dying and surviving neurons, thus suggesting that random fluctuations in pro-survival gene expression likely influence the effects of TBI on vulnerable brain cells, i.e., determine whether a neuron dies or survives following TBI. Since these microarray studies were costly, labor-intensive, and a bioinformatics challenge, in separate studies we investigated whether gene expression in limited numbers of laser captured neurons could also be delineated using pathway-focused PCR arrays. Because the majority of molecular biology laboratories have access to thermal cyclers capable of generating quantitative, real-time PCR data, we tested a qPCR-based method (RT^2^ Profiler PCR arrays) with the features of a miniarray. Since these PCR arrays are comprised of functionally related genes in disease-associated and/or known cell signaling pathways, they are designed to interrogate expression of groups of genes that are functionally and coordinately regulated, and they have the added benefit of providing immediate information about activation or inhibition of key canonical pathways without resorting to extensive bioinformatics analysis. The broad diversity of known cellular pathways covered by PCR arrays allows us to test our hypotheses about the biological roles of specific genes in identified populations of neurons. Although previous studies have used, for example, cDNA arrays [11], genome-wide arrays [12], Taqman pathway PCR arrays [13] or single-nucleotide polymorphism arrays [14] to examine gene expression in laser captured cells from salivary gland, breast, prostate, and glioblastoma tissues, respectively, to our knowledge this is the first study to use pathway-focused arrays for analysis of laser capture microdissected hippocampal neurons from brain injured rats.

Here, using an established rat model of experimental brain injury and LCM techniques that we have successfully employed to study the molecular mechanisms of TBI in enriched populations of brain cells [10,15–19], we used PCR arrays to compare gene expression data in dying, Fluoro-Jade positive and surviving, Fluoro-Jade negative hippocampal neurons obtained by LCM. Fluoro-Jade, which we have used extensively to identify dying neurons after TBI, is an anionic fluorochrome which binds with great sensitivity and specificity to dying, degenerating neurons identified as such by classical, well accepted methods [20,21]. Our study shows that PCR arrays can provide significant, biologically relevant insights into the effects of TBI in the hippocampus, an area of the brain central for learning and memory [22, 23].

## Experimental procedures

### Animal preparations for fluid percussion brain injury

The Institutional Animal Care and Use Committee of The University of Texas Medical Branch approved all experimental protocols. Adult, male, Charles River Sprague-Dawley rats (300–400 g) were anesthetized with 4% isoflurane, intubated, then mechanically ventilated. A craniotomy was performed laterally to the sagittal suture, midway between the lambda and bregma structures. The fluid percussion device was attached, and the animal was subjected to lateral fluid percussion traumatic brain injury (TBI) as previously described [15]. Each animal was injured at a moderate-to-severe level. The rats were sacrificed 24 hours after injury. Animals were euthanized using methods consistent with the AVMA Guidelines for the Euthanasia of Animals, 2013 Edition, Section S2.2.2.1, Inhaled Agents. Animals were deeply anesthetized in a small chamber containing 5% isoflurane delivered by a precision vaporizer at a flow rate sufficient to saturate the chamber in approximately one minute. Animals remained in the chamber for more than 8 minutes to ensure deep anesthesia. Death was confirmed by decapitation, brains dissected out, placed on dry ice for 10 minutes, and stored at -80°C until LCM was performed.

### Tissue preparation

Brains were removed from -80°C and allowed to warm up to -25°C in a cryostat. The tissue was embedded in Tissue-Tek O.C.T. (Sakura, Hayward CA) mounting medium and prepared for cryosectioning. 10 μM serial coronal sections of the hippocampus were collected on pre-cleaned slides (VWR, West Chester PA). Sections were fixed in 75% ethanol for 1 min, rinsed in RNase free water for 1 min, counterstained with 1% cresyl violet for 15 to 20 sec, rinsed in RNase free water 2X30 sec, stained with 0.001% Fluoro-jade (Histo-Chem, Inc., Jefferson AR) for 4 min, rinsed in RNase free water 3X1min, dehydrated in 95% ethanol for 30 sec, 100% ethanol for 30 sec, then cleared in xylene 2X3 min each. Sections were then air dried in a fume hood for approximately 10 min before proceeding to LCM as previously described [10].

### Laser capture microdissection (LCM)

LCM was performed using a PixCell IIe laser capture microscope with an infrared diode laser (Arcturus Engineering, Mountain View CA) as previously described [10]. Based on an estimate of neuronal cell size of 20–30μm, we determined that three captured neurons from three 10μm thick sections contained the total RNA equivalent of a single cell. Thus, 1500 captured cells, the equivalent of 500 dying/injured (Fluoro-Jade positive) neurons were captured onto the thermoplastic film of a CapSure Macro LCM Cap (Arcturus Engineering, Mountain View CA) from the CA1/CA3 subfields of the hippocampus; each biological replicate of cells were pooled from 3 TBI brains (9 brains total). The smallest laser spot size (7.5um) was used, and the power set to 65–80mW with pulse duration of 0.40–0.50 (these last 2 settings were adjusted for optimal capture of single neurons). The cells were lysed in 100 μl of lysis buffer (Ambion, Austin TX) and vortexed before storing at -80°C. 500 adjacent uninjured (Fluoro-Jade negative) neurons were also captured, lysed, and stored in this same manner. We should point out that slight contamination of dying cells with some surviving cells and/or processes of supporting glial cells is virtually unavoidable despite the small size of the laser.

### RNA isolation

500 injured/dying (FJ+) neurons from 3 TBI brains were pooled to create one biological sample of injured/dying cells. The same was repeated for the 500 uninjured/surviving (FJ-) neurons. Pooling small samples of cells for array analysis is considered advantageous in cases where the level of biological variation is high compared to technical variation on the array [24]. There were three such biological pools of injured and uninjured samples (from a total of 9 TBI rats) that were assayed with individual PCR arrays. Total RNA from each injured/uninjured pool was isolated using the RNAqueous-Micro kit (Ambion, Austin TX), following the manufacturer’s RNAqueous-Micro Protocol for LCM. Genomic DNA was removed from each sample by treatment with rDNase at 37°C for 20 minutes (Ambion, Austin TX). The injured/uninjured samples were run through the Agilent Bioanalyzer (Agilent Technologies, Santa Clara CA) to check both RNA quality and quantity. Our RIN values for our samples ranged from as low as 4 to as high as 9 but averaged between 5 and 7, which is in line with values reported in the literature [25,26]**.** Afterwards, approximately 1.2 ng of each sample were precipitated with 3M Sodium Acetate and 100% ethanol, and resuspended in 10 μl of nuclease-free water in preparation for the *RT* ^*2*^ *Nano PreAMP cDNA synthesis* kit (SA Biosciences, Frederick MD).

### cDNA synthesis and amplification

1.2 ng of injured and uninjured total RNA were reverse transcribed using the first strand cDNA synthesis kit (SA Biosciences, Frederick MD), omitting the gDNA elimination buffer step. Following the manufacturer’s protocol, 5 μl of cDNA was then preamplified using either the Neurotrophin and Receptors or Apoptosis array-specific primers and the *RT* ^*2*^ *Nano PreAMP cDNA synthesis* kit (SA Biosciences, Frederick MD). Cycling conditions for the preamplification of each cDNA sample was as follows: 1 cycle (10min) at 95°C, 12 cycles of (15sec at 95°C, then 2 min at 60°C). Samples were then placed on ice where 2ul of a side reaction reducer was added, incubated at 37°C for 15 minutes, then heat inactivated at 95°C for 5 minutes.

### PCR Arrays

The amplified cDNA was then diluted with nuclease-free water and added to the *RT* ^*2*^ *qPCR SYBR green Master Mix* (SA Biosciences, Frederick MD). 25 μl of the experimental cocktail was added to each well of the rat *Neurotrophin and Receptor PCR array* (SA Biosciences, Frederick MD) or the rat *Apoptosis PCR array* (SA Biosciences, Frederick MD). Real-Time PCR was performed on the Mx3000P QPCR System (Stratagene, La Jolla CA) and used SYBR green detection with the following thermal profile: segment 1 – 1 cycle: 95°C for 10 minutes, segment 2 – 40 cycles: 95°C for 15 seconds followed by 60°C for 1 minute, segment 3 (dissociation curve) – 95°C for 1 minute, 55°C 30 seconds, and 95°C for 30 seconds. All data from the PCR was collected by the MXPro software (provided by Stratagene with purchase of Mx3000 Multiplex Quantitative PCR System) and analyzed by SA Bioscience’s PCR Array Data Analysis Web Portal.

### Analysis of Real-Time PCR array data

Each array contained 5 separate housekeeping genes (*RPLP1*, *HPRT*, *RPL13A*, *LDHA*, and *ACTB*) that were used for normalization of the sample data. Normalization to the house keeping genes (HKG) was performed by calculating the ΔCt for each gene of interest (GOI) in the plate (Ct value of GOI-Ct value of HKG). Any C_t_ value >35 was considered to be a negative call. If the C_t_ value of the genomic DNA control was >30, then no genomic DNA was detectable. The RT^2^ Profiler PCR Array data analysis software calculates the fold change based on the widely used and agreed upon ∆∆ Ct method first described by Livak K.J. and Schmittgen T.D. in 2001 [27]. The RT^2^ software averages the triplicate (biological) normalized expression levels for each gene (ΔCt), raw data shown in S3 Table, before calculating ∆∆Ct between one control (surviving cells) and one experimental group (dying cells). Only individual pairwise comparisons are performed, not any ΔCt comparison across multiple groups at the same time. The software allows for the ability to define the best reference genes for normalization with guidance and recommendations from the recommendations made in the following publication by Vandesompele et al., [28]. Arikawa et al, compared the results of the original MicroArray Quality Control (MAQC) study with the RT Profiler platform and reported the PCR arrays deliver gene expression data that is highly comparable with TaqMan PCR and high-density microarrays [29].

### Statistical analysis of PCR array data

The RT^2^ Profiler PCR Array Data Analysis software does not perform any statistical analysis beyond the calculation of p-values using a Student’s t-test (two tail distribution and equal variances between the two samples) based on the triplicate 2^(-ΔC_T_) values for each gene in the injured group compared to the surviving group. The Microarray Quality Control (MAQC) published results indicating that a ranked list of genes based on fold-change and such a p-value calculation was sufficient to demonstrate reproducible results across multiple microarray and PCR Arrays including the RT^2^ Profiler PCR Arrays [30,31].

### Immunofluorescence analysis

Rats were anesthetized with sodium pentobarbital (70 mg/kg, i.p.) 24 hours after TBI, transcardially perfused and fixed with freshly prepared ice-cold 4% paraformaldehyde (PFA) in 0.1 M phosphate-buffered saline (PBS, pH 7.4). The brain was removed, post-fixed in 4% PFA and cryoprotected by infiltration in 20% sucrose solution in PBS overnight at 4°C. The tissue was rapidly frozen in O.C.T. embedding medium (Tissue Tek, Sakura, Tokyo). Frozen serial sections (10 μm thick) were cut on a cryostat (Leica CM1850, Wetzlar, Germany), placed on plus slides (VWR, West Chester, Pennsylvania), and stored at –20°C until needed. Frozen sections were blocked and permeabilized for 30 minutes at room temperature with 0.3% Triton X-100 in PBS containing 5% normal goat serum. After washing in PBS, sections were incubated with primary antibodies diluted in PBS overnight at 4°C. After washing in PBS, sections were incubated for 1 hour at room temperature with Alexa-conjugated secondary antibodies (Alexa-594: 1:400 dilution; Molecular Probes, Eugene, Oregon). The sections were washed 2 times for 10 minutes each in PBS and once in tap water, and incubated in 0.06% potassium permanganate in tap water for 1 minute. The sections were then washed in tap water for 5 minutes and incubated with Fluor-Jade C (0.0001% in tap water with 0.1% acetic acid) for 10 minutes. After three more washes in tap water (1 minute each), sections were cover- slipped with acidic mounting media (0.1% acetic acid/80% glycerol in tap water).

For the detection of CD40, the rats were anesthetized and euthanized by decapitation. The brains were rapidly removed, quickly frozen on dry ice, and embedded in O.C.T. embedding medium. Frozen serial sections (10 μm thick) were cut as described above and stored at –80°C until needed. The sections were fixed in methanol pre-chilled at -20°C for 20 minutes before immunofluorescence analysis.

The slides were viewed with an Olympus BX51 fluorescence microscope equipped with a cooled CCD camera (Microfire by Optronics, Goleta, CA) and image acquisition software (PictureFrame, Optronics, Goleta, CA).

## Results

### Laser capture microdissection of identified neurons

**Fig 1:**
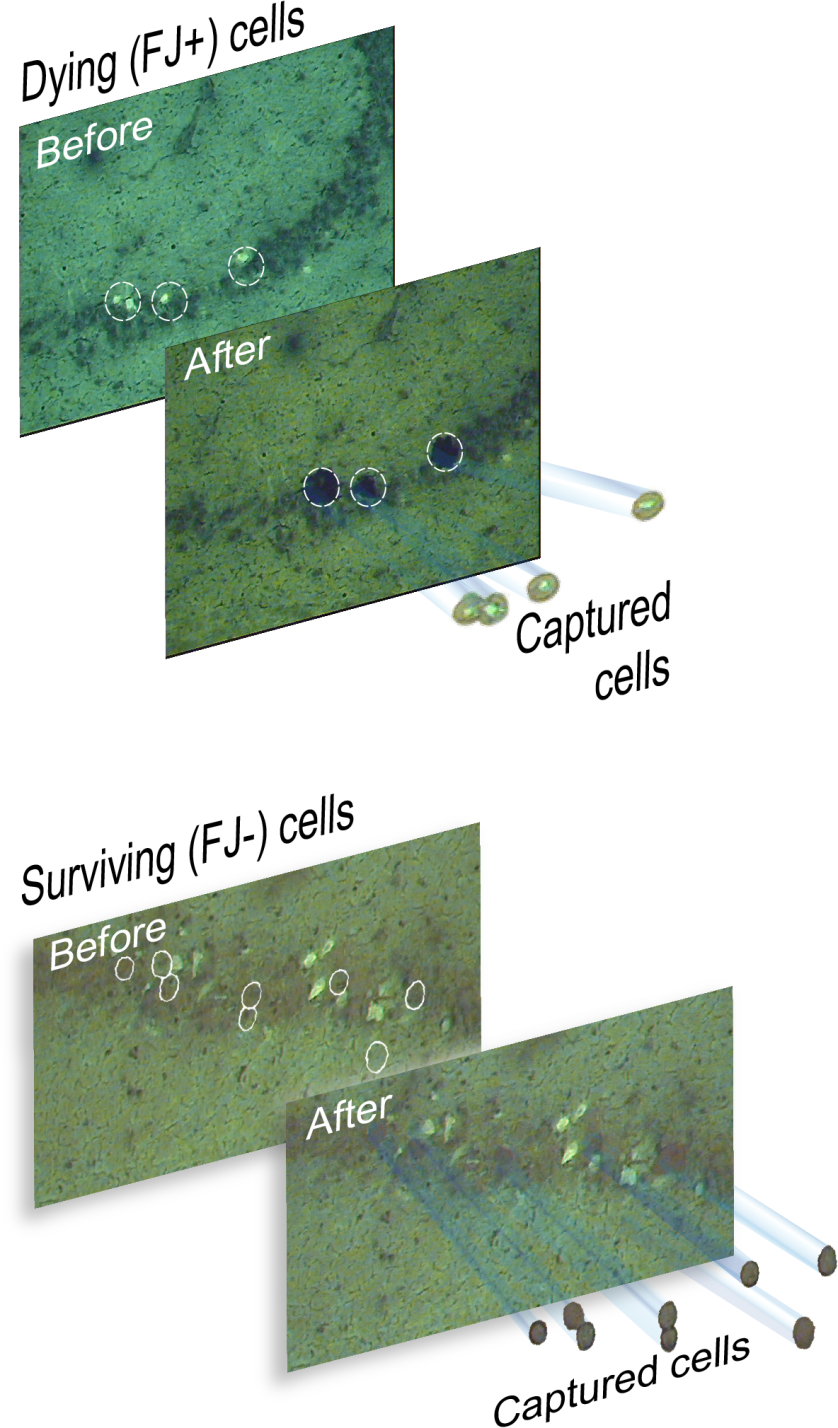
Laser capture microdissection of rat hippocampal neurons after fluid percussion brain injury. (A). Dying, Fluoro-Jade-positive neurons in the CA3 subfield of the rat hippocampus 24 hr after TBI are shown, before and after LCM on the capture caps. (B). Surviving, Fluoro-Jade negative neurons were captured immediately adjacent to FJ positive, dying neurons.

We used LCM to obtain enriched pools of dying, degenerating, Fluoro-Jade (FJ)-positive pyramidal neurons from the injured rat hippocampal CA1-CA3 subfields (Fig 1A). Enriched pools of surviving, FJ-negative pyramidal neurons immediately adjacent to FJ-positive neurons were also captured onto separate LCM caps (Fig 1B). The quality and quantity of isolated total RNA samples were assessed using the ultrasensitive pico assay on the Agilent Bioanalyzer (S1 Fig). Overall, the RNA quality from LCM procedures was excellent, in many cases matching the quality of total RNA isolated from hippocampal tissue samples using established TRIzol-based methods. This is in spite of the fact that RNA isolated from laser capture samples tends to give lower RNA integrity (RIN) values; this typically reflects the long arduous laser microdissection techniques [25]. Moreover, in all our previous LCM studies, our analysis showed a similar uniformity in RNA quality [10,17,18]. This gave us confidence that gene expression data from these experimental LCM RNA samples obtained using any established gene expression profiling platform would be reliable. Each biological sample from approximately 500 cells yielded several nanograms of total RNA, which was sufficient starting material for preamplification of the RNA samples [32] and subsequent real time PCR array analysis.

### Expression of apoptosis-associated genes in dying neurons

**Fig 2:**
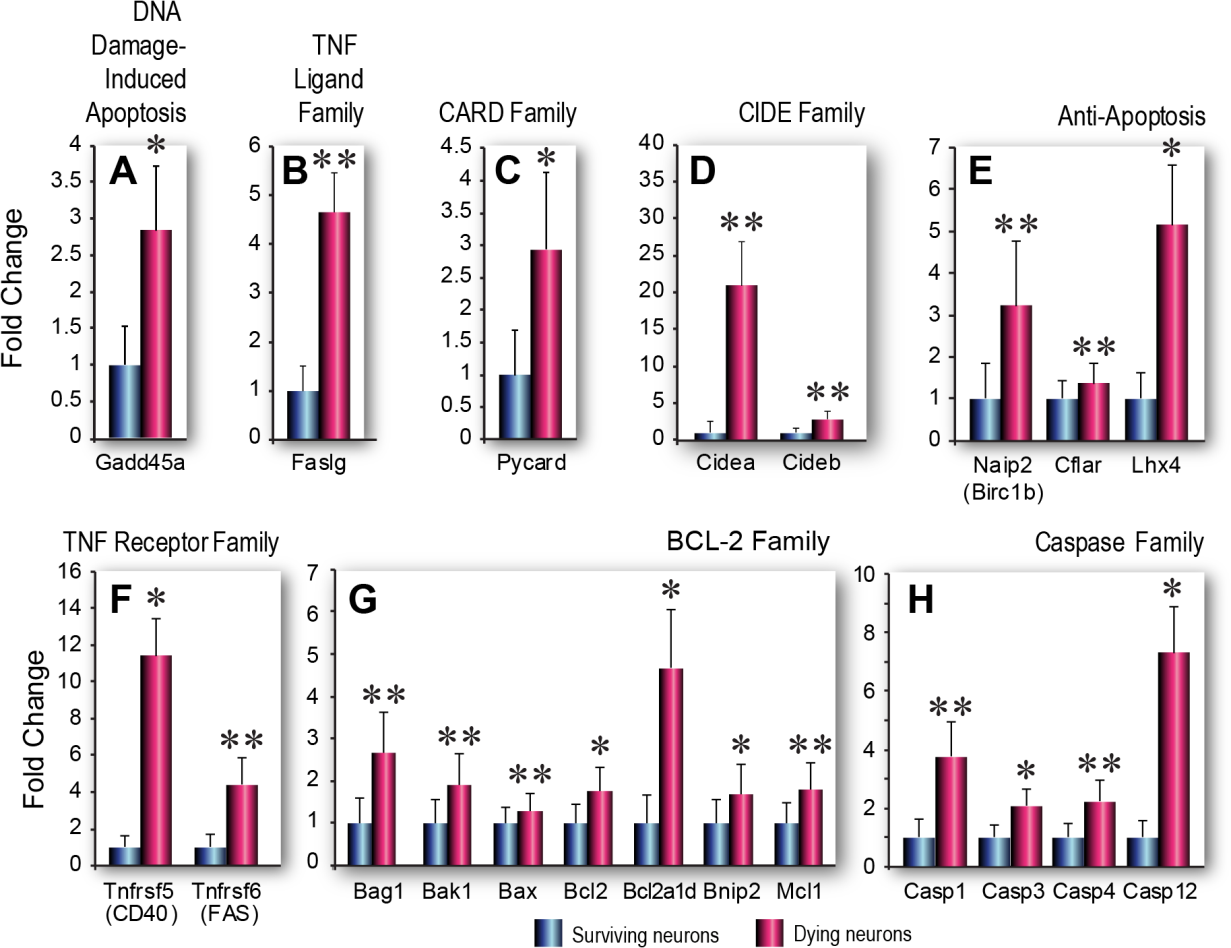
Apoptosis-related gene expression in dying and surviving neurons. A-H. Functional groups of genes involved in programmed cell death that are upregulated with significant (p<0.05) or borderline significance (0.05 < p < 0.1) in dying vs surviving neurons. (A) DNA Damage-induced Apoptosis. (B) TNF Ligand Family. (C) CARD Family. (D) CIDE Family. (E) Anti-apoptosis. (F) TNF Receptor Family. (G) Bcl-2 Family. (H) Caspase Family. Data are shown as fold changes in mRNA expression in dying compared to surviving cells, mean ± SEM (n = 3 biological pools each of Fluoro-Jade positive or Fluoro-Jade negative cells). Statistical analysis was performed using Student’s t-test, *p< 0.05, **p<0.1

In the apoptosis PCR array, we found that, compared to adjacent, surviving FJ-negative neurons, dying, FJ-positive pyramidal neurons had significantly higher expression of many genes known to be associated with apoptotic cell death (Table 1, S1 Table, fold changes are shown as ratios of gene expression in dying vs surviving neurons). Among these were multiple genes associated with the caspase family of cysteine proteases [33,34], the pro- and anti-apoptotic genes in the *Bcl-2* family [35] and genes in the *TNF* receptor family [36–38] that possess pro-survival and/or pro-death functions (Fig 2A–2H). *In silico* analysis of gene functions in the PCR array confirmed that the majority of these genes are associated with apoptotic cell death. Moreover, we found that expression of most of the differentially expressed genes (particularly those that were significantly different or of borderline significance) has been linked to TBI pathology (Table 1). Complete gene expression data and supporting references are provided in S1 and S2 Tables and in S1 References, raw data shown in S3 Table. For instance, although *Gadd45a* was expressed with borderline significance in our study, previous studies have shown that this gene is involved in oxidative stress-induced apoptotic cell death in hippocampal neurons [39]. Thus, increased expression of this gene in dying neurons is consistent with its known functions. Our observation that several significantly upregulated genes in dying neurons, such as *Pycard* (also known as *Asc*) are known to be involved in inflammatory cell death cascades [40] is consistent with a large number of TBI studies showing that inflammatory processes play a prominent role in neuronal injury and death [41]. On the other hand, we noted that several genes, such as *Bnip2*, that were increased significantly or with borderline significance have previously been shown to have multifunctional or pleiotropic roles in cell signaling processes [42]. The apoptosis array was initially chosen because we anticipated that if the array data was to be deemed biologically relevant, then apoptosis-related genes should be differentially expressed at significant levels in dying neurons compared to surviving neurons. Thus, our study serves as a training set for future studies of identified cell populations with postulated functions.

**Table 1:** Differential expression of Apoptosis and Neurotrophin genes in dying and surviving neurons.

Apoptosis PCR Array								
**Unigene**	**Refseq**	**Symbol**	**Description**	**Gene Name**	**Fold Change**	**p-value**	**Gene Card**	**PubMed Links**
Rn.81078	NM_130422	Casp12	Caspase 12	-	7.3276	0.000217	Casp12	I. Mehmeti et al. 2011 (DOI); K. Shimoke et al. 2004 (DOI); O. Diaz-Horta et al. 2002 (DOI)
Rn.25180	NM_134360	Cd40	CD40 molecule, TNF receptor superfamily member 5	Tnfrsf5	11.3924	0.000815	Cd40	E. Ripoll et al. 2013 (DOI); H. Sun et al. 2008 (DOI)
Rn.10562	NM_012922	Casp3	Caspase 3	Lice/ MGC93645	2.0801	0.002937	Casp3	G. Kanbak et al. 2013 (DOI); C. Espinosa-Garcia et al. 2013 (DOI)
Rn.19770	NM_133416	Bcl2a1d	B-cell leukemia/ lymphoma 2 related protein A1d	Bcl2a1	4.6697	0.005411	Bcl2a1d	C. M. Cartagena et al. 2013 (DOI)
Rn.9996	NM_016993	Bcl2	B-cell CLL/ lymphoma 2	Bcl-2	1.7532	0.007618	Bcl2	W. Mao et al. 2013 (DOI); H. Sin et al. (DOI)
Rn.48080	NM_001108348	Lhx4	LIM homeobox 4	-	5.1575	0.01244	Lhx4	T.-M. Hung et al. 2011 (DOI); A. Goc et al. 2012 (DOI)
Rn.44218	NM_053353	Cd40lg	CD40 ligand	Tnfsf5	5.6438	0.013359	Cd40lg	D. Obregon et al. 2008 (DOI); N. Y. Calingasan et al. 2002 (DOI)
Rn.9725	NM_012908	Faslg	Fas ligand (TNF superfamily, member 6)	Apt1Lg1/ CD95-L/ Fasl/ Tnfsf6	4.6482	0.020486	Faslg	N. Shioda et al. 2007 (DOI); Y. Sun et al. 2009 (DOI)
Rn.7817	NM_172322	Pycard	PYD and CARD domain containing	Asc	2.9349	0.023679	Pycard	J. Masumoto et al. 2002 (DOI)
Rn.16320	NM_001106647	Bag1	BCL2-associated athanogene	-	2.6697	0.026695	Bag1	T. Xu et al. 2012 (DOI); V. lanchamp et al. 2008 (DOI)
Rn.14598	NM_053812	Bak1	BCL2-antagonist/ killer 1	MGC108627	1.8877	0.03968	Bak1	C. Brooks et al. 2007 (DOI)
Rn.10250	NM_024127	Gadd45a	Growth arrest and DNA-damage-inducible, alpha	Ddit1/ Gadd45	2.8415	0.04049	Gadd45a	M. Sarkisian & D. Siebzhenrubl et al. 2012 (DOI)
Rn.11821	NM_001106835	Bnip2	BCL2/ adenovirus E1B interacting protein 2	-	1.6896	0.042881	Bnip2	Y. T. Zhou et al. 2005 (DOI)
Rn.129914	NM_021846	Mcl1	Myeloid cell leukemia sequence 1	-	1.7818	0.054103	Mcl1	S. M. Mahmudul Hasan et al. 2013 (DOI)
Rn.10668	NM_017059	Bax	Bcl2-associated X protein	-	1.2834	0.056162	Bax	X.-J. Zou et al. 2012 (DOI); W. Mao et al. 2013 (DOI)
Rn.204016	NM_001108869	Cideb	Cell death-inducing DFFA-like effector b	-	3.6723	0.059174	Cideb	S. Tiwari et al. 2013 (DOI); Z. Chen et al. 2010 (DOI)
Rn.16195	NM_053736	Casp4	Caspase 4, apoptosis-related cysteine peptidase	Casp11/ MGC124949	2.2346	0.064688	Casp4	J. Hitomi et al. 2004 (DOI); S.-J. Kim et al. 2006 (DOI)
Rn.162521	NM_139194	Fas	Fas (TNF receptor superfamily, member 6)	Tnfrsf6	4.4178	0.064856	Fas	C. G. Besirli et al. 2011 (DOI); X. H. Yin et al. 2013 (DOI)
Rn.37508	NM_012762	Casp1	Caspase 1	Ice/ Il1bc	3.7581	0.074822	Casp1	M. Sifringer et al. 2007 (DOI); G. Nilufer Yonguc et al. 2012 (DOI)
Rn.204752	NM_057138	Cflar	CASP8 and FADD-like apoptosis regulator	Flip/ MGC108616	1.3566	0.081339	Cflar	K. Järvinen et al. 2011 (DOI); Y. Matsumori et al. 2006 (DOI)
Rn.8171	NM_001170467	Cidea	Cell death-inducing DFFA-like effector a	-	26.1125	0.091347	Cidea	N. Omae et al. 2012 (DOI); M. Ito et al. 2011 (DOI)
Rn.92423	XM_226742	Naip2	NLR family, apoptosis inhibitory protein 2	Birc1/ Birc1a/ Birc1b/ Naip	3.2266	0.095072	Naip2	M. Ito et al. 2011 (DOI)
Rn.83633	NM_130426	Tnfrsf1b	Tumor necrosis factor receptor superfamily, member 1b	Tnfr2	3.3096	0.098788	Tnfrsf1b	M. S. Weinberg et al. 2013 (DOI)
Neurotrophins & Receptors PCR Array								
**Unigene**	**RefSeq**	**Symbol**	**Description**	**Gene Name**	**Fold Change**	**p-value**	**GeneCard**	**PubMed Links**
Rn.55036	NM_019139	Gdnf	Glial cell derived neurotrophic factor	-	14.0367	0.011829	Gdnf	Y.-.M Yoo et al.2012 (DOI); I. Kanter-Schlifke et al. 2009 (DOI); J. E. Minnich et al. 2012 (DOI); L.-F. Wong et al. 2006 (DOI)
Rn.973	NM_181091	Gmfg	Glia maturation factor, gamma	-	4.8159	0.017405	Gmfg	H. Tsuiki et al. 2000 (DOI)
Rn.24822	NM_019172	Galr2	Galanin receptor 2	-	3.3122	0.025542	Galr2	R. Toifghi et al. 2008 (DOI); Y. Yang et al. 2006 (DOI)
Rn.137580	NM_022205	Cxcr4	Chemokine (C-X-C motif) receptor 4	MGC108696	29.8805	0.036531	Cxcr4	A. J. Shepherd et al. 2012 (DOI); V. Ödemis et al. 2002 (DOI); X. Liu et al. 2013 (DOI)
Rn.34398	NM_030997	Vgf	VGF nerve growth factor inducible	-	2.0202	0.060712	Vgf	J. Adler et al. 2003 (DOI); G.-L. Ferri et al. 2011 (DOI)
Rn.204252	NM_022196	Lif	Leukemia inhibitory factor	-	11.6678	0.078427	Lif	B. E. Deverman & P. H. Patterson et al. 2012 (DOI)

Differentially expressed genes that were deemed significant (p<0.05) or of borderline significance (p<0.1) are shown. The complete datasets are shown in S1 and S2 Tables. Hyperlinks to the GeneCard entry for each gene and Digital Object Identifier (DOI) for at least one supporting published reference are included. GeneCards is a searchable, integrated, database of human genes that provides concise genomic related information, on all known and predicted human genes.

Fold changes are ratios of gene expression levels (dying/surviving neurons).

We noted that several prominent pro-survival genes, such as *Bcl-2* and *Mcl1* [35,43], were expressed at significantly higher levels in the dying neurons than in adjacent surviving neurons. The neuropeptide galanin and its receptors (*GalR1* and *GalR2*) have previously been shown to be involved in hippocampal neuron survival [44]; therefore, it was not surprising to find that *GalR* expression-the gene for galanin is not on the PCR array list-was significantly increased after TBI. We should like to emphasize that in our study, the use of LCM allowed us to demonstrate for the first time that *GalR* expression is significantly higher in dying neurons than surviving neurons.

### Expression of neurotrophins and their receptor genes in dying neurons

**Fig 3:**
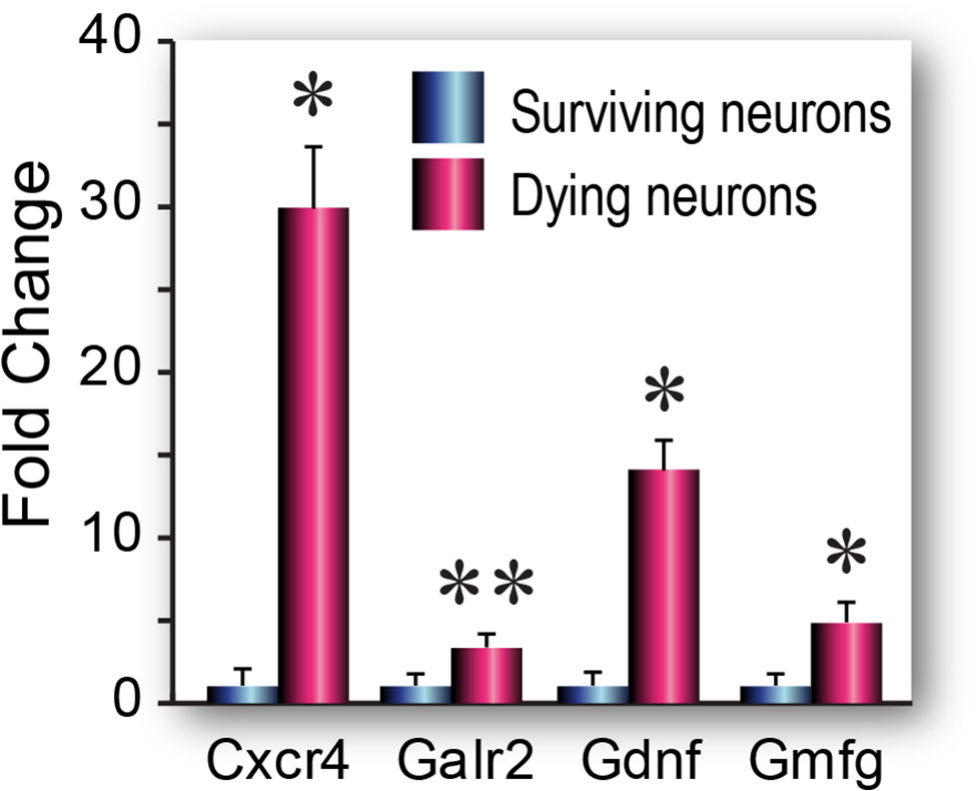
Neurotrophins & Receptors PCR array gene expression in dying and surviving neurons. Data are shown as fold changes (mean ± SEM) (n = 3 biological pools each of Fluoro-Jade positive or Fluoro-Jade negative cells) in dying cells compared to surviving cells. Statistical analysis was performed using Student’s t test, *p< 0.05, **p<0.1

The genes on the Neurotrophins & Receptors PCR array represent a broad range of normal neuronal functions. Because the two groups of dying and surviving hippocampal pyramidal neurons are morphologically and functionally similar, we were not surprised that there were fewer numbers of differentially expressed genes (Fig 3, Table 1, S2 Table). On the other hand, we found that the few genes from this array that were upregulated in dying neurons were either associated with neurodegenerative disorders when expressed at high levels, e.g., glia maturation factor gamma (*Gmfγ*) is a proinflammatory gene previously linked to Alzheimer’s pathology [45], or were known to promote pro-survival functions after brain injury (e.g., *Gdnf)* [46]. As we found in the apoptosis array, several of the genes upregulated in dying neurons are known to have pleiotropic functions—e.g., increased expression of chemokine receptors such as CXCR4 have been linked to hippocampal damage and neuronal loss in HIV patients [47], but this gene has also been shown to modulate hippocampal plasticity and neurogenesis [48].

### Immunohistochemical validation of gene expression data

**Fig 4:**
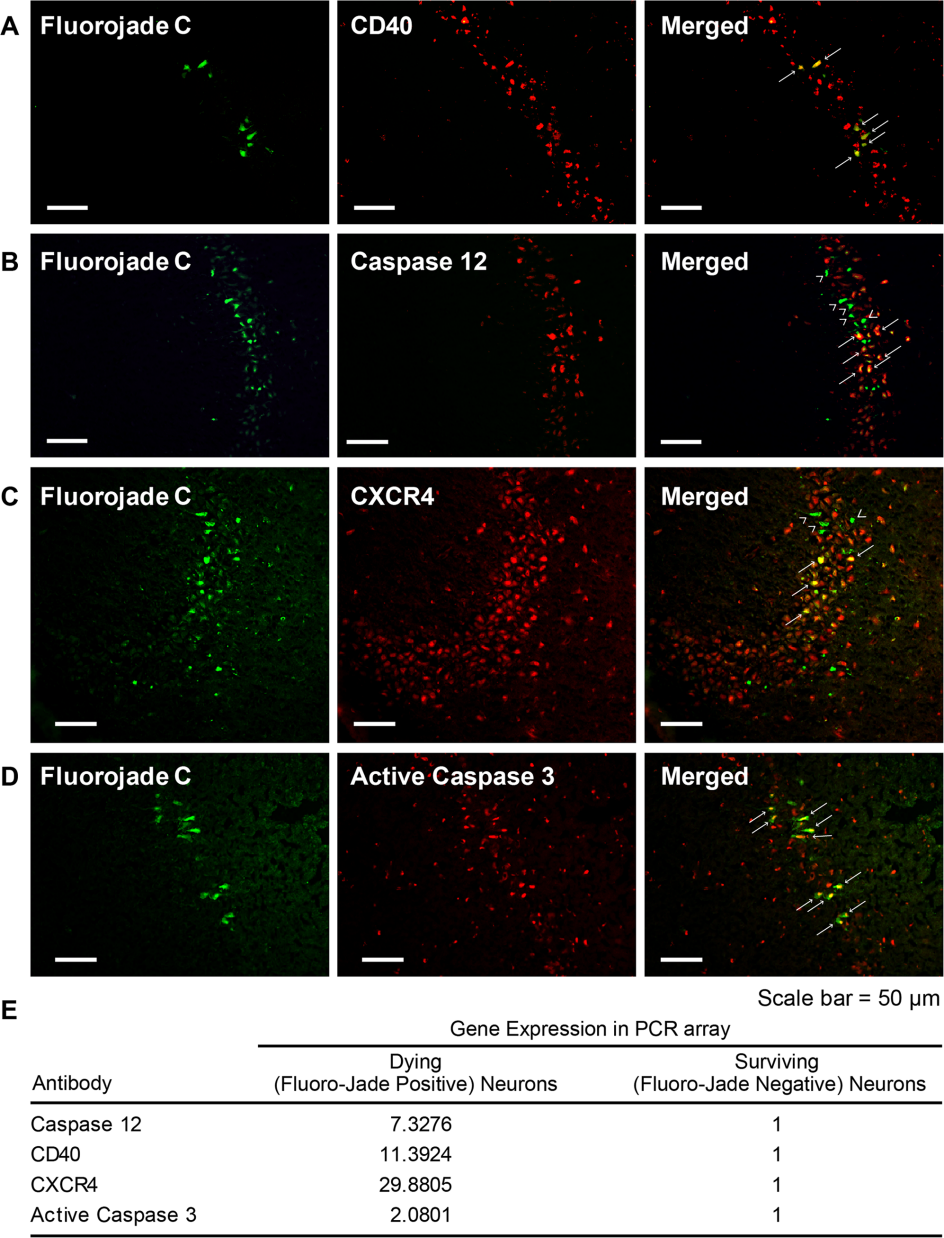
Immunohistochemical validation of gene expression data. Representative images of rat brain sections double-stained, using Fluoro-Jade C (FJ, in green) to identify injured neurons, and specific antibodies against CD40 (A), CASP 12 (B), CXCR4 (C) and active caspase 3 (D). Arrows point to cells co-expressing Fluoro-Jade C and the protein of interest. Arrowheads point to cells expressing low levels of CASP12 (B) and CXCR4 (C). (E) Gene expression in dying and surviving neurons, expressed as fold changes in dying to surviving neurons (normalized to a value of 1) are shown as a point of reference for the protein expression data. Calibration bars = 50 mm

Double immunofluorescence analysis using specific antibodies against a selected pool of proteins, whose mRNA was significantly increased in FJ-positive neurons as compared to adjacent FJ-negative neurons, was performed in order to confirm the PCR array data (Fig 4). Our data show that, 24 hours following TBI, the TNF receptor CD40 is highly expressed in FJ-positive pyramidal neurons of the hippocampus, although it is also detected, albeit at low levels, in FJ-negative neurons (Fig 4A). We found that both CASP12 and CXCR4were highly expressed in some but not all FJ-positive cells, thus indicating that a certain degree of stochasticity exists at the protein level (Fig 4B and 4C). In order to confirm that FJ-positive cells are indeed irreversibly injured, we performed immunofluorescence analysis using an antibody that specifically recognizes the active form of CASP3, an enzyme that plays a critical role in the activation of the apoptotic cascade. Our results show that active caspase 3 is expressed in FJ-positive neurons but not in FJ-negative neurons (Fig 4D), confirming the PCR array results, i.e., a significant increase in the expression of *Casp3* mRNA in degenerating neurons as compared to adjacent uninjured neurons.

We also sought to confirm, using immunofluorescence analysis, the differential expression of the protein products of some of the genes for which differential expression was of borderline significance or not statistically significant, e.g., LIF, DAD1, STAT3 and TGFα. Our reasoning was that mRNA expression often correlates poorly with protein expression [49] and some of the statistically insignificant changes in gene expression (due to high variability in the RNA samples) may actually correspond to significant differences at the level of protein expression. However, our results were equivocal; specific staining for each of these proteins was detected in the injured rat hippocampus, but it could not be unambiguously determined whether expression of these proteins was higher in dying compared to surviving neurons (S2 Fig). Therefore, the immunohistochemical data neither confirmed nor refuted the gene expression data and further studies may be needed to validate individual gene expression data.

## Discussion

This is the first report of a study utilizing pathway-focused PCR-based arrays to examine TBI-induced gene expression in dying and surviving hippocampal neurons obtained by LCM. Genome-wide microarray analysis is a hybridization-based method which works well for moderate to relatively abundant transcripts but is not ideal for detecting genes expressed at low levels. On the other hand, PCR is an amplification-based method and because of this, PCR arrays could technically be far more sensitive with a greater dynamic range than microarrays. Previous studies have reported use of pathway specific PCR arrays to examine gene expression in breast tissue after pregnancy to ascertain mechanisms of cancer risk [50] and to identify genetic markers of angiogenesis in laser dissected blood vessels [51]. PCR arrays have also been used to determine molecular mechanisms of brain damage and neuroprotection in manually microdissected samples of the ipsilateral cortex containing the ischemic penumbra after experimental transient focal ischemia [52] and to study the effects of progesterone treatment after TBI [53]. However, none of these brain injury studies involved LCM. As these arrays can generate gene expression data from as little as 1 nanogram of total RNA, our study shows that they are ideal tools for gene expression studies of small numbers of laser captured cells.

In addition to providing support for the use of PCR arrays for limited numbers of laser captured cells, the second purpose of this study was to gain insight into the prosurvival mechanisms induced *in vivo* in injured neurons after TBI. We reasoned that knowing the identity of genes involved in endogenous protection will help in design of pharmacotherapeutic strategies targeted towards increasing the expression or activity of these genes and possibly mitigating the effects of TBI on the injured brain cells. Previous studies have used *in vitro* approaches to identify neuroprotective genes, such as the ones involved in the endogenous protective response induced by preconditioning paradigms [54]. We suggest that our approach is more biologically relevant, as we are precisely determining TBI-induced gene expression *in situ* in defined populations of neurons.

Comparing our PCR results with microarray data [10], we were surprised that only 9 genes on the PCR arrays were also found on the filtered microarray dataset (S4 Table). However, because the microarray data has the potential for significant numbers of false positives, in our previous study we had used a stringent 5-fold cutoff to study gene expression differences between dying and surviving neurons and we suggest that this, along with the greater dynamic range of the PCR-based method as well as the pre-selected (thus, biased) gene lists, could explain the lack of extensive concordance between the microarrays and PCR arrays. On the other hand, the trends (increase or decrease) in gene expression in all 9 common genes were concordant in both the microarray and PCR array datasets. We also surmised another positive outcome of this study, that despite the pathway-focused and limited gene sets, the overall sensitivity of the PCR arrays allowed us to detect gene expression differences that were not apparent in the transcriptome analysis.

We chose to profile dying and surviving hippocampal neurons as proof-of-concept because we could easily identify these two populations of neurons with a well characterized fluorescent marker of neurodegeneration, Fluoro-Jade [20]. Additionally, by using LCM to capture enriched pools of dying or surviving neurons, we have greatly increased the resolution of the gene expression analysis over earlier studies of laser captured neurons that sampled broad swaths of neurons from injured brains and contained a mix of dying and surviving cell types [55]. We show that profiling distinct populations of identified neurons can provide valuable information about mechanisms of cell death by contrasting our cell-specific results with that of a recent article about the neurodegenerative effects of blast-induced neurotrauma in the rat hippocampus [56]. In that study, the authors showed that expression of apoptotic genes *Bax* and *Casp3* were increased in the injured hippocampus, evidenced by overall increased Fluoro-Jade B staining throughout the hippocampus. However, unlike our study, the authors did not assess gene expression in selected, identified populations of dying and surviving neurons. Thus, their results merely imply that dying cells express apoptotic genes, whereas our study shows clearly that apoptotic gene expression is higher in the identified dying cells compared with surviving cells.

The value of using precise LCM techniques for identified cells cannot be overstated. A cDNA array study of apoptosis-related genes showed that *Mcl-1* was down-regulated in the cerebella of *Igf-1* transgenic mice. However, subsequent Northern analysis and LCM of cerebellar granule neurons followed by qPCR analysis showed that expression of this gene was actually increased in cerebellar granule neurons compared to non-transgenic littermates [57]. Thus, when gene expression analysis of a distinct cell type was performed, the results were completely the opposite of that gained from analysis of a heterogeneous mix of brain cell types in the cerebellum. On the other hand, because we were able to examine gene expression in relatively pure populations of defined neurons, we have greater confidence in the definitive conclusions we made about the causative role of specific apoptosis related genes in neuronal cell death. Studies similar to ours could be performed using identified groups of immunolabeled cells obtained by LCM [58]. Because of the great heterogeneity of cell types in the mammalian brain, as many as 2500–5000 cell types [59], gene expression studies of identifiable and functionally distinct cell populations that can be obtained by LCM are essential in efforts to elucidate disease-associated pathogenesis.

Our study results also bolstered our previous observation that a strong, protective response is induced in both dying and surviving neurons after TBI [10]. Interestingly, in the present study we found that several, well known genes associated with cell survival were actually expressed significantly higher in dying neurons compared to surviving neurons. This seems to be counterintuitive, but the prominent expression of many other deleterious genes in the dying cells appears to counter the beneficial effects. *In silico* analysis of genes that were differentially expressed revealed that many of these also possessed pleiotropic functions, i.e., immune-modulatory genes such as *Cxcr4* had functional roles in critical cellular processes such as synaptic plasticity but also were implicated in hippocampal injury [47,48]. Bcl-2-associated athanogene-1 (*Bag1*), a co-chaperone for *Hsp70/Hsc70*, is also a multifunctional protein that has been shown to suppress apoptosis and enhance neuronal differentiation. The TBI-induced increases in *Bag1* and *Casp12*, (the former reduces endoplasmic reticulum [ER] stress and apoptosis [60] while the latter increases ER stress and apoptosis [61]), show that multiple pro-survival- and pro-death-associated genes are upregulated in both dying and surviving cells. Thus, the difference in cell fate is likely due to a preponderance of prosurvival gene expression in surviving neurons and a preponderance of prodeath genes in dying neurons. This is the same conclusion that we drew in our microarray study, thus showing that use of different gene expression platforms can lead to similar insights.

Immunohistochemical analysis of differentially expressed gene products clearly showed that protein expression was a continuum rather than an all or none phenomenon. The expression of any one gene was also variable from cell to cell, indicating that the stochastic nature of TBI-induced gene expression is also reflected in protein expression. This was not unexpected since the poor correlation of gene and protein expression is well known [49]. Protein expression of the genes that were not statistically significant or were of borderline significance was particularly variable, and we were unable to correlate the higher expression in dying neurons at the mRNA level with protein expression. However, we noted that only the expression of LIF had borderline significance; protein expression of the other three non-significant genes was examined because their mRNA expression was higher in dying neurons and each had been functionally implicated in pro- or anti-apoptotic functions [62–64]. Interestingly, like several other genes with borderline significance (S1 References), LIF is known to possess pleiotropic functions in both prosurvival and pathogenic pathways [65]. Because of this functional duality, even at the resolution of single cells, it cannot be determined whether LIF is playing a beneficial or deleterious role in dying cells.

In contrast, it was clear that the pro-apoptotic genes, *Casp12*, *Casp3*, *Cxcr4* and *CD4*, that were found to be statistically significantly differentially expressed between dying and surviving neurons, were prominently colocalized in dying, FJ positive neurons. The extensive colocalization of the protein-specific antibodies with the FJ stain provided a mechanistic explanation and support for the statistically significant increased expression found in the qPCR array experiments. The colocalization of the immunolabeling and FJ was particularly striking for two genes that are strongly implicated in cell death, CD40 and active caspase 3. These data also validated the accuracy and precision of the LCM experiments [66,67]. On the other hand, we should point out that even pro-death associated genes, such as CD40, have also been shown to have prominent roles in neuronal differentiation and proliferation [68]. This indicates that the interpretation of cell-specific gene expression of any apoptosis –related gene is entirely dependent on the context, and we should be careful about drawing definitive conclusions about the functional role of any gene without additional supporting evidence. However, the validation of gene expression data at the protein level gave us confidence in the qPCR array results, and has spurred current studies of drug effects using other pathway-focused PCR arrays.

Pathway-specific PCR arrays have several advantages for different applications. One, they can quickly provide clinically relevant data without the need for the extensive bioinformatics analysis necessary to decipher the results of genome-wide gene arrays. For instance, Hansel et al. showed that PCR array analysis could distinguish an allergy-associated gene expression profile of CD4+ T cells in allergic patients compared to the more cumbersome data obtained from Affymetrix oligonucleotide arrays [69]. Second, as shown in our present study, PCR array analysis of LCM samples provides information about specific cellular mechanisms that cannot be gleaned from profiling heterogeneous tissues comprised of multiple cell types. Marciano et al. compared expression in caspase positive dying neurons to that of uninjured neurons from uninjured mice [70]. Using LCM to procure pure or enriched cell populations, we were able to refine the original experimental design in the Marciano study and focus on why neurons subjected to the same injury as adjacent dying neurons in the same injured animal are able to survive TBI. In future studies, we plan to design and use custom PCR arrays of identified neuroprotective genes to test our hypothesis that TBI induces a significant protective response in the injured brain and that drugs that enhance this protective response will improve functional outcome after TBI.

Several therapeutic agents in clinical use for other indications might be able to boost the expression of endogenous neuroprotective genes such as the ones identified in our study. For instance, antidepressants have been shown to increase expression of GDNF in cultured cells [71]. Because depression is a common comorbidity in TBI patients [72], it is possible that those who are treated with antidepressants may be benefiting from the neuroprotective effects of these treatments. Notably, edaravone, a free radical scavenger drug currently in clinical trials for various neurological disorders, has been shown to be neuroprotective by enhancing expression of BDNF, Bcl-2 and suppressing caspase-3 activity [73]. Drugs targeted to single mechanisms of brain injury have failed to consistently improve outcome in clinical trials, so one current line of thinking is that pharmacotherapeutic agents with multiple mechanisms of action (i.e., antioxidative and anti-inflammatory, etc.) are more likely to be successful [74]. Indeed, our study shows that genes involved in multiple pathological and prosurvival processes are significantly induced in dying neurons, and multifunctional drugs that target these genes may have more efficacy than drugs with single targets in reducing cell death after TBI.

## Supporting Information

S1 Fig Analysis of total RNA isolated from laser capture microdissected hippocampal neurons from traumatically brain injured rats using the pico assay on the Agilent Bioanalyzer. Total RNA from each pool (500 cells) of dying or surviving neurons is assayed in duplicate.
(TIF)

S2 Fig Immunohistochemical analysis of proteins that were upregulated in dying neurons compared to surviving neurons in the PCR arrays with borderline significance (LIF) or not statistically significant (DAD1, TGFα, STAT3). The equivocal protein expression levels appear to validate the lack of significance in PCR arrays.
(TIF)

S1 Table Apoptosis PCR Array. (DOCX)

S2 Table Neurotrophins &Receptors PCR Array. (DOCX)

S3 Table Apoptosis and Neurotrophins & Receptors data (DOCX)

S4 Table Comparison of genes in Agilent microarrays and pathway-specific PCR arrays. (DOCX)

S1 References Lists of published reports supporting the functional roles of each detectable gene on both arrays. (DOC)
