# Supplementary figures and images for: Pathway-Focused PCR Array Profiling of Enriched Populations of Laser Capture Microdissected Hippocampal Cells after Traumatic Brain Injury
Source: PLoS One. 2015 May 27;10(5):e0127287. doi: 10.1371/journal.pone.0127287 (PMC4446038; doi:10.1371/journal.pone.0127287)

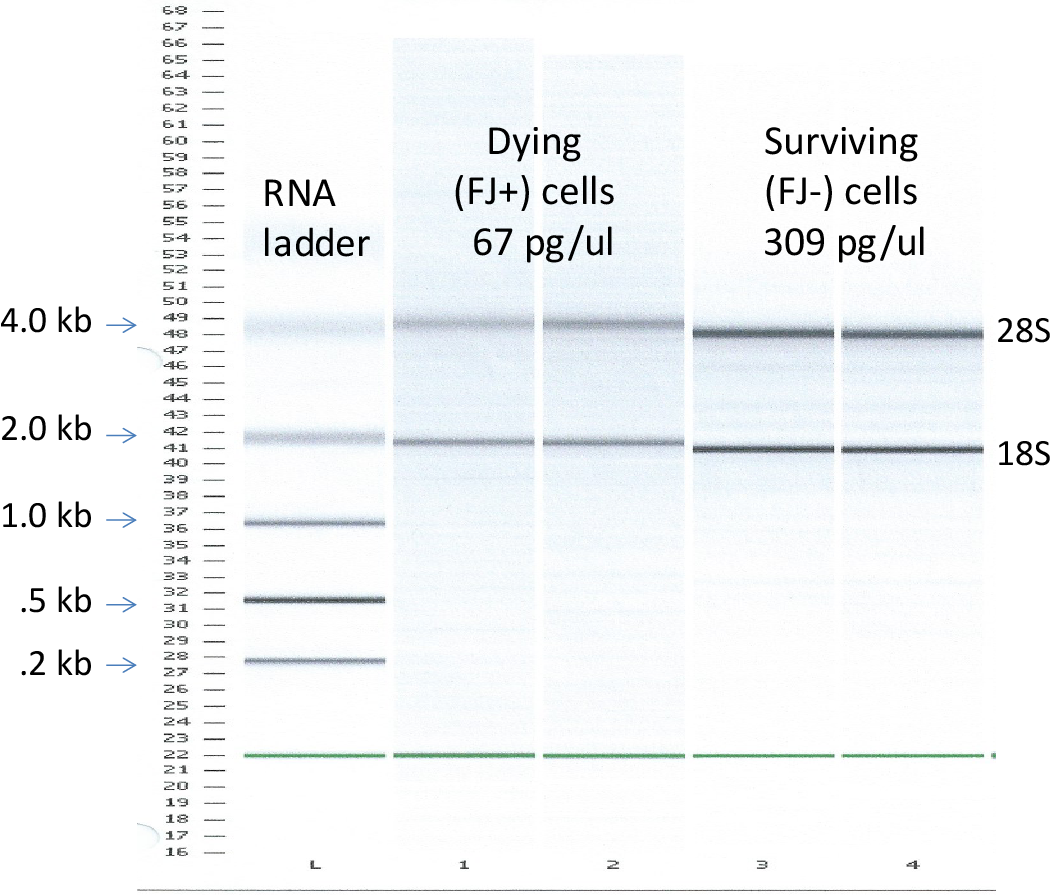

Supplement: S1 Fig — Total RNA from each pool (500 cells) of dying or surviving neurons is assayed in duplicate. (TIF) [file pone.0127287.s001.tif]

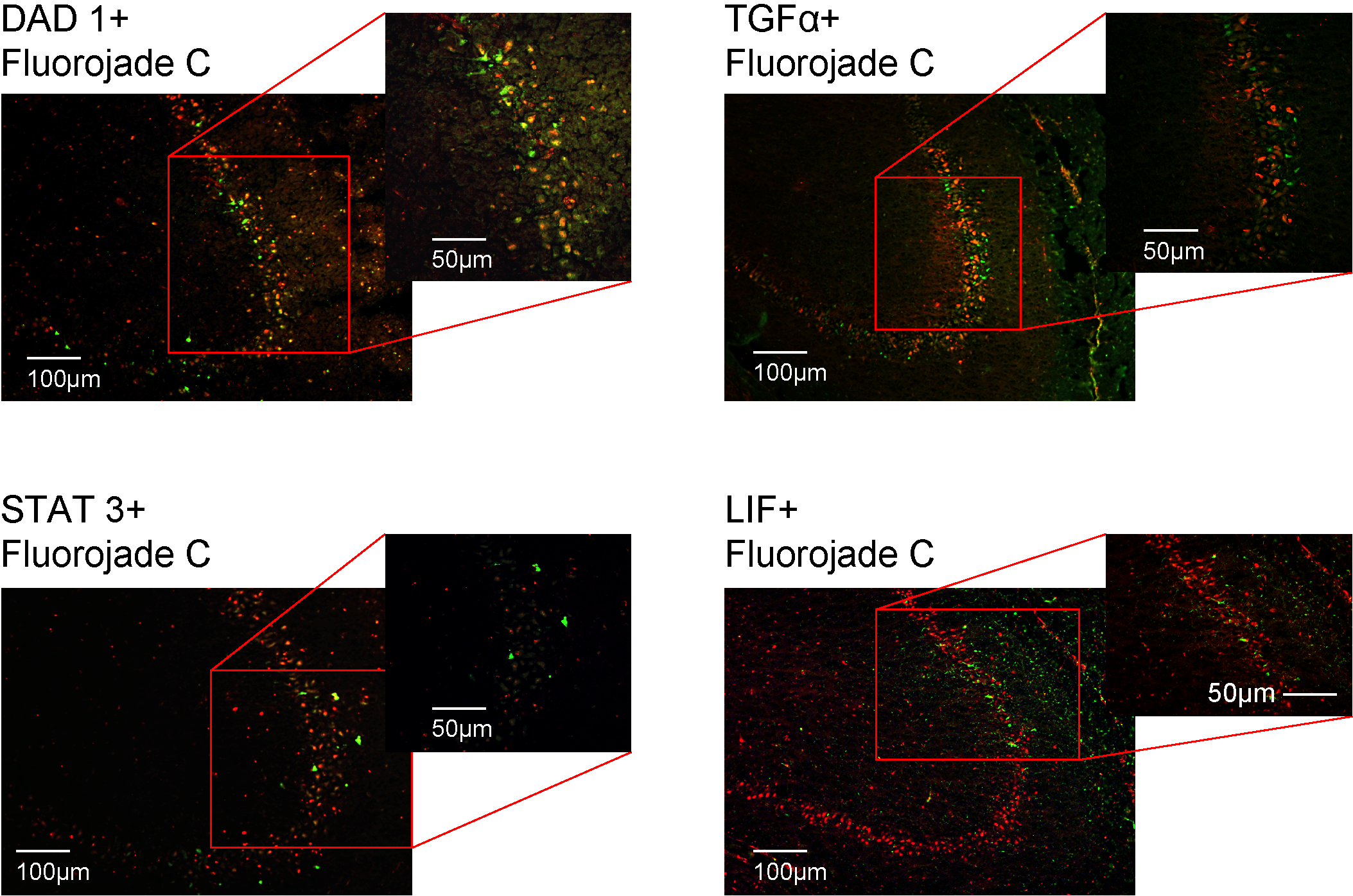

Supplement: S2 Fig — The equivocal protein expression levels appear to validate the lack of significance in PCR arrays. (TIF) [file pone.0127287.s002.tif]
