# Supplementary material for: Pathway-Focused PCR Array Profiling of Enriched Populations of Laser Capture Microdissected Hippocampal Cells after Traumatic Brain Injury
Source: PLoS One. 2015 May 27;10(5):e0127287. doi: 10.1371/journal.pone.0127287 (PMC4446038; doi:10.1371/journal.pone.0127287)
Supplement: S1 Table — (DOCX) [file pone.0127287.s003.docx]

**Table S1. Apoptosis PCR Array.**

Fold changes are shown as ratios of gene expression in dying versus surviving neurons.

| **Unigene** | **Refseq** | **Symbol** | **Description** | **Gene Name** | **Fold Change** | **p-value** | **Gene Card** | **PubMed Links** |
| --- | --- | --- | --- | --- | --- | --- | --- | --- |
| Rn.81078 | NM_130422 | Casp12 | Caspase 12 | - | 7.3276 | 0.000217 | [Casp12](http://www.genecards.org/cgi-bin/carddisp.pl?gene=Casp12) | I. Mehmeti et al. 2011 ([DOI](http://dx.doi.org/10.1016/j.bbamcr.2011.06.022));  K. Shimoke et al. 2004 ([DOI](http://dx.doi.org/10.1093/jb/mvh053%20));  O. Diaz-Horta et al. 2002 ([DOI](http://dx.doi.org/10.2337/diabetes.51.6.1815)) |
| Rn.25180 | NM_134360 | Cd40 | CD40 molecule, TNF receptor superfamily member 5 | Tnfrsf5 | 11.3924 | 0.000815 | [Cd40](http://www.genecards.org/cgi-bin/carddisp.pl?gene=Cd40) | E. Ripoll et al. 2013 ([DOI](http://www.plosone.org/article/info%3Adoi%2F10.1371%2Fjournal.pone.0065068));  H. Sun et al. 2008 ([DOI](http://dx.doi.org/10.1016/j.ijdevneu.2008.01.009)) |
| Rn.10562 | NM_012922 | Casp3 | Caspase 3 | Lice/ MGC93645 | 2.0801 | 0.002937 | [Casp3](http://www.genecards.org/cgi-bin/carddisp.pl?gene=Casp3) | G. Kanbak et al. 2013 ([DOI](http://dx.doi.org/10.1016/j.gene.2012.10.012));  C. Espinosa-Garcia et al. 2013 ([DOI](http://dx.doi.org/10.1016/j.neulet.2013.06.023)) |
| Rn.19770 | NM_133416 | Bcl2a1d | B-cell leukemia/ lymphoma 2 related protein A1d | Bcl2a1 | 4.6697 | 0.005411 | [Bcl2a1d](http://www.genecards.org/cgi-bin/carddisp.pl?gene=Bcl2a1d) | C. M. Cartagena et al. 2013 ([DOI](http://dx.doi.org/10.1007/s12031-012-9828-z)); |
| Rn.9996 | NM_016993 | Bcl2 | B-cell CLL/ lymphoma 2 | Bcl-2 | 1.7532 | 0.007618 | [Bcl2](http://www.genecards.org/cgi-bin/carddisp.pl?gene=Bcl2) | W. Mao et al. 2013 ([DOI](http://www.dx.doi.org/10.3109/00207454.2013.838236));  H. Sin et al. ([DOI](http://www.dx.doi.org/10.2174/1871527311312030011)) |
| Rn.48080 | NM_001108348 | Lhx4 | LIM homeobox 4 | - | 5.1575 | 0.01244 | [Lhx4](http://www.genecards.org/cgi-bin/carddisp.pl?gene=Lhx4) | T. -M. Hung et al. 2011 ([DOI](http://dx.doi.org/10.1093/carcin/bgr219));  A. Goc et al. 2012 ([DOI](http://dx.doi.org/10.1186/1471-2407-12-409)) |
| Rn.44218 | NM_053353 | Cd40lg | CD40 ligand | Tnfsf5 | 5.6438 | 0.013359 | [Cd40lg](http://www.genecards.org/cgi-bin/carddisp.pl?gene=Cd40lg) | D. Obregon et al. 2008 ([DOI](http://dx.doi.org/10.1016/j.nbd.2007.09.009));  N. Y. Calingasan et al. 2002 ([DOI](http://dx.doi.org/10.1016/S0197-4580(01)00246-9)) |
| Rn.9725 | NM_012908 | Faslg | Fas ligand (TNF superfamily, member 6) | Apt1Lg1/ CD95-L/ Fasl/ Tnfsf6 | 4.6482 | 0.020486 | [Faslg](http://www.genecards.org/cgi-bin/carddisp.pl?gene=Faslg) | N. Shioda et al. 2007 ([DOI](http://onlinelibrary.wiley.com/doi/10.1111/j.1471-4159.2007.04600.x/abstract)); Y. Sun et al. 2009 ([DOI](http://dx.doi.org/10.1016/j.bbalip.2008.09.007)) |
| Rn.7817 | NM_172322 | Pycard | PYD and CARD domain containing | Asc | 2.9349 | 0.023679 | [Pycard](http://www.genecards.org/cgi-bin/carddisp.pl?gene=Pycard) | J. Masumoto et al. 2002 ([DOI](http://dx.doi.org/10.1074/jbc.M203944200)); |
| Rn.16320 | NM_001106647 | Bag1 | BCL2-associated athanogene | - | 2.6697 | 0.026695 | [Bag1](http://www.genecards.org/cgi-bin/carddisp.pl?gene=Bag1) | T. Xu et al. 2012 ([DOI](http://dx.doi.org/10.1007/s10735-012-9408-0)); V. Planchamp et al. 2008 ([DOI](http://dx.doi.org/10.1093/brain/awn196)) |
| Rn.14598 | NM_053812 | Bak1 | BCL2-antagonist/ killer 1 | MGC108627 | 1.8877 | 0.03968 | [Bak1](http://www.genecards.org/cgi-bin/carddisp.pl?gene=Bak1) | C. Brooks et al. 2007 ([DOI](http://dx.doi.org/10.1073/pnas.0703976104)); |
| Rn.10250 | NM_024127 | Gadd45a | Growth arrest and DNA-damage-inducible, alpha | Ddit1/ Gadd45 | 2.8415 | 0.04049 | [Gadd45a](http://www.genecards.org/cgi-bin/carddisp.pl?gene=Gadd45a) | M. Sarkisian & D. Siebzhenrubl et al. 2012 ([DOI](http://dx.doi.org/10.1371/journal.pone.0044207)); |
| Rn.11821 | NM_001106835 | Bnip2 | BCL2/ adenovirus E1B interacting protein 2 | - | 1.6896 | 0.042881 | [Bnip2](http://www.genecards.org/cgi-bin/carddisp.pl?gene=Bnip2) | Y. T. Zhou et al. 2005 ([DOI](http://dx.doi.org/10.1016/j.yexcr.2004.08.044)); |
| Rn.129914 | NM_021846 | Mcl1 | Myeloid cell leukemia sequence 1 | - | 1.7818 | 0.054103 | [Mcl1](http://www.genecards.org/cgi-bin/carddisp.pl?gene=Mcl1) | S. M. Mahmudul Hasan et al. 2013 ([DOI](http://dx.doi.org/10.1242/dev.090910%20)); |
| Rn.10668 | NM_017059 | Bax | Bcl2-associated X protein | - | 1.2834 | 0.056162 | [Bax](http://www.genecards.org/cgi-bin/carddisp.pl?gene=Bax) | X. -J. Zou et al. 2012 ([DOI](http://dx.doi.org/10.1258/ebm.2012.012041)); W. Mao et al. 2013 ([DOI](http://dx.doi.org/10.3109/00207454.2013.838236)) |
| Rn.204016 | NM_001108869 | Cideb | Cell death-inducing DFFA-like effector b | - | 3.6723 | 0.059174 | [Cideb](http://www.genecards.org/cgi-bin/carddisp.pl?gene=Cideb) | S. Tiwari et al. 2013 ([DOI](http://dx.doi.org/10.1074/jbc.M112.434258));  Z. Chen et al. 2010 ([DOI](http://dx.doi.org/10.1074/jbc.M110.141598)) |
| Rn.16195 | NM_053736 | Casp4 | Caspase 4, apoptosis-related cysteine peptidase | Casp11/ MGC124949 | 2.2346 | 0.064688 | [Casp4](http://www.genecards.org/cgi-bin/carddisp.pl?gene=Casp4) | J. Hitomi et al. 2004 ([DOI](http://jcb.rupress.org/content/165/3/347.full.pdf+html));  S. -J. Kim et al. 2006 ([DOI](http://dx.doi.org/10.1093/hmg/ddl105)) |
| Rn.162521 | NM_139194 | Fas | Fas (TNF receptor superfamily, member 6) | Tnfrsf6 | 4.4178 | 0.064856 | [Fas](http://www.genecards.org/cgi-bin/carddisp.pl?gene=Fas) | C. G. Besirli et al. 2011 ([DOI](http://dx.doi.org/10.1167/iovs.10-7090));  X. H. Yin et al. 2013 ([DOI](http://dx.doi.org/10.1016/j.neuroscience.2013.06.012)) |
| Rn.37508 | NM_012762 | Casp1 | Caspase 1 | Ice/ Il1bc | 3.7581 | 0.074822 | [Casp1](http://www.genecards.org/cgi-bin/carddisp.pl?gene=Casp1) | M. Sifringer et al. 2007 ([DOI](http://dx.doi.org/10.1016/j.nbd.2006.11.003));  G. Nilufer Yonguc et al. 2012 ([DOI](http://dx.doi.org/10.1007/s11033-012-1913-4)) |
| Rn.204752 | NM_057138 | Cflar | CASP8 and FADD-like apoptosis regulator | Flip/ MGC108616 | 1.3566 | 0.081339 | [Cflar](http://www.genecards.org/cgi-bin/carddisp.pl?gene=Cflar) | K. Järvinen et al. 2011 ([DOI](http://dx.doi.org/10.1016/j.yexcr.2011.08.014));  Y. Matsumori et al. 2006 ([DOI](http://stroke.ahajournals.org/content/37/2/507.full.pdf+html)) |
| Rn.8171 | NM_001170467 | Cidea | Cell death-inducing DFFA-like effector a | - | 26.1125 | 0.091347 | [Cidea](http://www.genecards.org/cgi-bin/carddisp.pl?gene=Cidea) | N. Omae et al. 2012 ([DOI](http://dx.doi.org/10.1016/j.mce.2011.09.013));  M. Ito et al. 2011 ([DOI](http://dx.doi.org/10.1194/jlr.M012427)) |
| Rn.92423 | XM_226742 | Naip2 | NLR family, apoptosis inhibitory protein 2 | Birc1/ Birc1a/ Birc1b/ Naip | 3.2266 | 0.095072 | [Naip2](http://www.genecards.org/cgi-bin/carddisp.pl?gene=Naip2) | M. Ito et al. 2011 ([DOI](http://dx.doi.org/10.1194/jlr.M012427)) |
| Rn.83633 | NM_130426 | Tnfrsf1b | Tumor necrosis factor receptor superfamily, member 1b | Tnfr2 | 3.3096 | 0.098788 | [Tnfrsf1b](http://www.genecards.org/cgi-bin/carddisp.pl?gene=Tnfrsf1b) | M. S. Weinberg et al. 2013 ([DOI](http://dx.doi.org/10.1016/j.expneurol.2013.01.011)) |
| Rn.136874 | XM_001080233 | LOC687813 | Similar to Tnf receptor-associated factor 1 |  | 16.9514 | 0.101719 | [LOC687813](http://www.genecards.org/cgi-bin/carddisp.pl?gene=LOC687813) |  |
| Rn.23108 | NM_001107335 | Dapk1 | Death associated protein kinase 1 | - | 0.4559 | 0.106278 | [Dapk1](http://www.genecards.org/cgi-bin/carddisp.pl?gene=Dapk1) | S. Nair et al. 2013 ([DOI](http://dx.doi.org/10.3390/ijms140713858)) |
| Rn.3211 | NM_001001513 | Tnfsf12 | Tumor necrosis factor ligand superfamily member 12 | TWEAK | 2.2501 | 0.136259 | [Tnfsf12](http://www.genecards.org/cgi-bin/carddisp.pl?gene=Tnfsf12) | E. Rousselet et al. 2012 ([DOI](http://dx.doi.org/10.1124/mol.112.079608));  R. Echeverry et al. 2012 ([DOI](http://dx.doi.org/10.1186/1742-2094-9-45)) |
| Rn.19329 | NM_001008315 | Ltbr | Lymphotoxin beta receptor (TNFR superfamily, member 3) | MGC94657 | 2.3839 | 0.161475 | [Ltbr](http://www.genecards.org/cgi-bin/carddisp.pl?gene=Ltbr) | L. Onder et al. 2013 ([DOI](http://dx.doi.org/10.1084/jem.20121462));  Y. Wang et al. 2010 ([DOI](http://dx.doi.org/10.1016/j.immuni.2010.02.011)) |
| Rn.11119 | NM_013091 | Tnfrsf1a | Tumor necrosis factor receptor superfamily, member 1a | MGC105478/ Tnfr1 | 3.9086 | 0.166855 | [Tnfrsf1a](http://www.genecards.org/cgi-bin/carddisp.pl?gene=Tnfrsf1a) | Y. Wang et al. 2012 ([DOI](http://dx.doi.org/10.1371/journal.pone.0043436));  DM. Sama et al. 2012 ([DOI](http://dx.doi.org/10.1371/journal.pone.0038170)) |
| Rn.2275 | NM_012675 | Tnf | Tumor necrosis factor (TNF superfamily, member 2) | MGC124630/ RATTNF/ TNF-alpha/ Tnfa | 3.2868 | 0.170138 | [Tnf](http://www.genecards.org/cgi-bin/carddisp.pl?gene=Tnf) | R. Waters et al. 2013 ([DOI](http://dx.doi.org/10.1089/neu.2012.2792)) |
| Rn.41053 | NM_001012066 | Sphk2 | Sphingosine kinase 2 | - | 0.5141 | 0.193435 | [Sphk2](http://www.genecards.org/cgi-bin/carddisp.pl?gene=Sphk2) | LD. Mastrandrea et al. 2010 ([DOI](http://dx.doi.org/10.1194/jlr.M000802)); LM. Young et al. 2012 ([DOI](http://dx.doi.org/10.1161/STROKEAHA.111.626911)) |
| Rn.64522 | NM_023979 | Apaf1 | Apoptotic peptidase activating factor 1 | - | 0.6894 | 0.225031 | [Apaf1](http://www.genecards.org/cgi-bin/carddisp.pl?gene=Apaf1) | C. Johnson et al. 2007 ([DOI](http://dx.doi.org/10.1073/pnas.0709101105));  OP. Mishra et al. 2010 ([DOI](http://dx.doi.org/10.1016/j.neulet.2010.05.081)) |
| Rn.106419 | NM_080895 | Faim | Fas apoptotic inhibitory molecule | - | 0.6522 | 0.225286 | [Faim](http://www.genecards.org/cgi-bin/carddisp.pl?gene=Faim) | TJ. Schneider et al. 1999 ([DOI](http://dx.doi.org/10.1084/jem.189.6.949)) |
| Rn.54474 | NM_022277 | Casp8 | Caspase 8 | - | 2.8812 | 0.228476 | [Casp8](http://www.genecards.org/cgi-bin/carddisp.pl?gene=Casp8) | M. Kraiewska et al. 2011 ([DOI](http://dx.doi.org/10.1371/journal.pone.0024341));  K. Jarvinen 2011 ([DOI](http://dx.doi.org/10.1016/j.yexcr.2011.08.014)) |
| Rn.198715 | XM_232860 | Casp8ap2 | Caspase 8 associated protein 2 | - | 2.3784 | 0.235216 | [Casp8ap2](http://www.genecards.org/cgi-bin/carddisp.pl?gene=Casp8ap2) | HW. Kim et al. 2009 ([DOI](http://dx.doi.org/10.1074/jbc.M109.020925)) |
| Rn.145049 | NM_001107757 | Aven | Apoptosis, caspase activation inhibitor | - | 0.5535 | 0.255463 | [Aven](http://www.genecards.org/cgi-bin/carddisp.pl?gene=Aven) | K. Olzisik et al. 2006 ([DOI](http://www.sciencedirect.com/science/article/pii/S0041134506010402)) |
| Rn.44461 | NM_017312 | Bok | BCL2-related ovarian killer | - | 0.7354 | 0.263491 | [Bok](http://www.genecards.org/cgi-bin/carddisp.pl?gene=Bok) | L. Soane et al. 2007 ([DOI](http://dx.doi.org/10.1002/jnr.21584)); F.  Ke et al. 2013 ([DOI](http://dx.doi.org/10.1038/cddis.2013.176)) |
| Rn.16757 | NM_080897 | Bnip1 | BCL2/ adenovirus E1B interacting protein 1 | - | 0.7371 | 0.266069 | [Bnip1](http://www.genecards.org/cgi-bin/carddisp.pl?gene=Bnip1) | C. Bongki et al. 2012 ([DOI](http://dx.doi.org/10.1007/s10059-012-0051-0)) |
| Rn.16183 | NM_152937 | Fadd | Fas (TNFRSF6)-associated via death domain | Mort1 | 0.473 | 0.268501 | [Fadd](http://www.genecards.org/cgi-bin/carddisp.pl?gene=Fadd) | C. Mc Guire et al. 2010 ([DOI](http://www.jimmunol.org/content/185/12/7646.short)) |
| Rn.88160 | NM_031775 | Casp6 | Caspase 6 | MGC93335/ Mch2 | 1.7492 | 0.276674 | [Casp6](http://www.genecards.org/cgi-bin/carddisp.pl?gene=Casp6) | R. Graham et al. 2010 ([DOI](http://dx.doi.org/10.1523/JNEUROSCI.2071-10.2010));  V. Uribe et al. 2012 ([DOI](http://dx.doi.org/10.1093/hmg/dds005)) |
| Rn.32199 | NM_031632 | Casp9 | Caspase 9, apoptosis-related cysteine peptidase | Apaf3/ Casp-9-CTD/ Casp9_v1/ Ice-Lap6/ Mch6 | 0.6613 | 0.279784 | [Casp9](http://www.genecards.org/cgi-bin/carddisp.pl?gene=Casp9) | R. Hakem et al. 1998? ([DOI](http://dx.doi.org/10.1016/S0092-8674(00)81477-4)) |
| Rn.38487 | NM_053704 | Bik | BCL2-interacting killer (apoptosis-inducing) | Biklk/ Blk | 7.3956 | 0.284132 | [Bik](http://www.genecards.org/cgi-bin/carddisp.pl?gene=Bik) | E. Lomonosova et al. 2009 ([DOI](http://dx.doi.org/10.1038/onc.2009.39)) |
| Rn.7262 | NM_138910 | Dad1 | Defender against cell death 1 | - | 0.8351 | 0.298959 | [Dad1](http://www.genecards.org/cgi-bin/carddisp.pl?gene=Dad1) | M. Rosen et al. 2005 ([DOI](file:///J:\HLH\2013\2013-08-xx%20Super%20array%20paper\original%20files\http.\dx.doi.org\10.1016\j.reprotox.2004.10.005)) |
| Rn.202973 | NM_012870 | Tnfrsf11b | Tumor necrosis factor receptor superfamily, member 11b | MGC93568/ Opg | 1.7092 | 0.30769 | [Tnfrsf11b](http://www.genecards.org/cgi-bin/carddisp.pl?gene=Tnfrsf11b) | TJ. Song et al. 2012 ([DOI](http://dx.doi.org/10.3109/1354750X.2012.727027)) |
| Rn.138066 | NM_001130554 | Card10 | Caspase recruitment domain family, member 10 | - | 2.308 | 0.30785 | [Card10](http://www.genecards.org/cgi-bin/carddisp.pl?gene=Card10) | Mx. Chang et al. 2009 ([DOI](http://dx.doi.org/10.1016/j.dci.2009.08.002)) |
| Rn.18545 | NM_001100480 | Tradd | TNFRSF1A-associated via death domain | - | 1.5764 | 0.30859 | [Tradd](http://www.genecards.org/cgi-bin/carddisp.pl?gene=Tradd) | S. Chakraborty et al. 2013 ([DOI](http://www.neoplasia.com/pdf/manuscript/v15i08/neo13608.pdf)) |
| Rn.53995 | NM_022260 | Casp7 | Caspase 7 | - | 7.5336 | 0.309645 | [Casp7](http://www.genecards.org/cgi-bin/carddisp.pl?gene=Casp7) | S. Larner et al. 2005 ([DOI](http://dx.doi.org/10.1111/j.1471-4159.2005.03172.x)) |
| Rn.2060 | NM_053420 | Bnip3 | BCL2/ adenovirus E1B interacting protein 3 | MGC93043 | 0.6799 | 0.320226 | [Bnip3](http://www.genecards.org/cgi-bin/carddisp.pl?gene=Bnip3) | R. Schmidt-Kastner et al. 2004 ([DOI](http://dx.doi.org/10.1016/j.brainres.2003.11.065)) |
| Rn.36696 | NM_022698 | Bad | BCL2-associated agonist of cell death | MGC72439 | 0.683 | 0.327261 | [Bad](http://www.genecards.org/cgi-bin/carddisp.pl?gene=Bad) | S. Yu Has et al. 1997 ([DOI](http://dx.doi.org/10.1210/me.11.12.1858)) |
| Rn.105232 | NM_001107815 | Traf2 | Tnf receptor-associated factor 2 | - | 1.2002 | 0.328346 | [Traf2](http://www.genecards.org/cgi-bin/carddisp.pl?gene=Traf2) | S. Shinoda et al. 2003 ([DOI](http://dx.doi.org/10.1046/j.1460-9568.2003.02655.x)) |
| Rn.211872 | NM_138852 | Prok2 | Prokineticin 2 | Bv8 | 1.8489 | 0.339955 | [Prok2](http://www.genecards.org/cgi-bin/carddisp.pl?gene=Prok2) | MY. Cheng et al. 2012 ([DOI](http://dx.doi.org/10.1073/pnas.1113363109)) |
| Rn.44266 | NM_053777 | Mapk8ip1 | Mitogen-activated protein kinase 8 interacting protein 1 | JIP1/ Jip-1/ Mapk8ip | 1.3692 | 0.350913 | [Mapk8ip1](http://www.genecards.org/cgi-bin/carddisp.pl?gene=Mapk8ip1) | M. Scheinfeld et al. 2003 ([DOI](http://dx.doi.org/10.1074/jbc.M304853200)) |
| Rn.6514 | NM_053679 | Dffa | DNA fragmentation factor, alpha subunit | ICAD-S | 0.7579 | 0.353491 | [Dffa](http://www.genecards.org/cgi-bin/carddisp.pl?gene=Dffa) | J. Slane et al. 2002 ([DOI](http://dx.doi.org/10.1016/S0031-9384(02)00716-3)) |
| Rn.10323 | NM_031535 | Bcl2l1 | Bcl2-like 1 | Bcl-xl/ Bcl2l/ Bclx/ bcl-X | 1.5087 | 0.356015 | [Bcl2l1](http://www.genecards.org/cgi-bin/carddisp.pl?gene=Bcl2l1) | Q. Xiao et al. 2012 ([DOI](http://www.jneurosci.org/content/32/39/13587.long)) |
| Rn.9757 | NM_012630 | Prlr | Prolactin receptor | MGC105486/ RATPRLR | 5.0865 | 0.367101 | [Prlr](http://www.genecards.org/cgi-bin/carddisp.pl?gene=Prlr) | R. Dutia et al.2012 ([DOI](http://dx.doi.org/10.1016/j.peptides.2012.07.006)) |
| Rn.47 | NM_012583 | Hprt1 | Hypoxanthine phosphoribosyltransferase 1 | Hgprtase/ Hprt/ MGC112554 | 0.7649 | 0.38904 | [Hprt1](http://www.genecards.org/cgi-bin/carddisp.pl?gene=Hprt1) | M. Camici et al. 2010 ([DOI](http://dx.doi.org/10.1016/j.neuint.2009.12.003)) |
| Rn.2511 | NM_017169 | Prdx2 | Peroxiredoxin 2 | Tdpx1 | 0.7561 | 0.398161 | [Prdx2](http://www.genecards.org/cgi-bin/carddisp.pl?gene=Prdx2) | SY. Shim et al. 2012 ([DOI](http://dx.doi.org/10.3109/10715762.2011.649749));  R. Leak et al. 2013 ([DOI](http://dx.doi.org/10.1161/STROKEAHA.111.680157)) |
| Rn.42907 | NM_019221 | Tp63 | Tumor protein p63 | Ket/ P73l/ Tp73l/ Trp63 | 2.214 | 0.445419 | [Tp63](http://www.genecards.org/cgi-bin/carddisp.pl?gene=Tp63) | NC. Hernandez-Acosta et al. 2011 ([DOI](http://dx.doi.org/10.1016/j.brainres.2010.11.041)) |
| Rn.64578 | NM_023987 | Birc3 | Baculoviral IAP repeat-containing 3 | Birc2/ IAP1/ MGC93416 | 2.6574 | 0.465176 | [Birc3](http://www.genecards.org/cgi-bin/carddisp.pl?gene=Birc3) | Y. Wang et al. 2012 ([DOI](http://dx.doi.org/10.1371/journal.pone.0043436)) |
| Rn.9346 | NM_017141 | Polb | Polymerase (DNA directed), beta | - | 0.7203 | 0.477368 | [Polb](http://www.genecards.org/cgi-bin/carddisp.pl?gene=Polb) | C. L. Gridley et al. 2013 ([DOI](http://dx.doi.org/10.1021/bi301368f%0a)) |
| Rn.83627 | NM_145681 | Tnfsf10 | Tumor necrosis factor (ligand) superfamily, member 10 | Trail | 1.8067 | 0.481825 | [Tnfsf10](http://www.genecards.org/cgi-bin/carddisp.pl?gene=Tnfsf10) | B. Pennarum et al 2010 ([DOI](http://dx.doi.org/10.1016/j.bbcan.2009.11.004)) |
| Rn.9868 | NM_012854 | Il10 | Interleukin 10 | IL10X | 4.1989 | 0.489497 | [Il10](http://www.genecards.org/cgi-bin/carddisp.pl?gene=Il10) | P. Gonzalez et al. 2009 ([DOI](http://histoserver.uab.es/PDFs/Publicaciones/P0000000103Gonzalez2009_JNEN.pdf)) |
| Rn.104526 | NM_001106413 | Card6 | Caspase recruitment domain family, member 6 | - | 0.816 | 0.497378 | [Card6](http://www.genecards.org/cgi-bin/carddisp.pl?gene=Card6) | A. Dufner et al. 2006 ([DOI](http://www.ncbi.nlm.nih.gov/pmc/articles/PMC1327733/pdf/pnas-0510380103.pdf)) |
| Rn.15652 | XM_214967 | Bclaf1 | BCL2-associated transcription factor 1 | Aa2-041 | 1.2114 | 0.519843 | [Bclaf1](http://www.genecards.org/cgi-bin/carddisp.pl?gene=Bclaf1) | H. Sarras et al. 2010 ([DOI](http://dx.doi.org/10.1100/tsw.2010.132)) |
| Rn.54471 | NM_022274 | Birc5 | Baculoviral IAP repeat-containing 5 | AP14 | 1.6358 | 0.534041 | [Birc5](http://www.genecards.org/cgi-bin/carddisp.pl?gene=Birc5) | BA. Mayer et al. 2011 ([DOI](http://dx.doi.org/10.1161/ATVBAHA.111.234294)) |
| Rn.67077 | NM_053362 | Dffb | DNA fragmentation factor, beta polypeptide (caspase-activated DNase) | Cad | 1.3503 | 0.547588 | [Dffb](http://www.genecards.org/cgi-bin/carddisp.pl?gene=Dffb) | V. Guimarais et al. 2013 ([DOI](http://dx.doi.org/10.1074/jbc.M112.411371)) |
| Rn.86956 | NM_053516 | Nol3 | Nucleolar protein 3 (apoptosis repressor with CARD domain) | Arc | 1.1865 | 0.573757 | [Nol3](http://www.genecards.org/cgi-bin/carddisp.pl?gene=Nol3) | Q. Wang et al. 2012 ([DOI](http://www.ijcep.com/files/IJCEP1205012.pdf)) |
| Rn.102179 | XM_342810 | Ripk2 | Receptor-interacting serine-threonine kinase 2 | - | 1.3947 | 0.581631 | [Ripk2](http://www.genecards.org/cgi-bin/carddisp.pl?gene=Ripk2) | P. J. Shaw et al. 2011 ([DOI](http://dx.doi.org/10.1016/j.immuni.2010.12.015)) |
| Rn.31142 | NM_022684 | Bid | BH3 interacting domain death agonist | - | 1.162 | 0.58683 | [Bid](http://www.genecards.org/cgi-bin/carddisp.pl?gene=Bid) | XM. Yin 2006 ([DOI](http://dx.doi.org/10.1016/j.gene.2005.10.038)) |
| Rn.44267 | NM_021850 | Bcl2l2 | Bcl2-like 2 | BCL-W/ BCL-WEL/ BCL-WS/ Bclw/ MGC91704 | 1.1329 | 0.594474 | [Bcl2l2](http://www.genecards.org/cgi-bin/carddisp.pl?gene=Bcl2l2) | L. Gibson et al 1996 ([DOI](http://www.ncbi.nlm.nih.gov/pubmed/8761287?dopt=Abstract)) |
| Rn.3219 | NM_001107017 | Traf4 | Tnf receptor associated factor 4 | - | 0.9223 | 0.653778 | [Traf4](http://www.genecards.org/cgi-bin/carddisp.pl?gene=Traf4) | S. Blaise et al. 2012 ([DOI](http://dx.doi.org/10.1371/journal.pone.0030917)) |
| Rn.85739 | NM_001108085 | Cradd | CASP2 and RIPK1 domain containing adaptor with death domain | RAIDD | 0.8274 | 0.668123 | [Cradd](http://www.genecards.org/cgi-bin/carddisp.pl?gene=Cradd) | O. Jabado et al. 2004 ([DOI](http://dx.doi.org/10.1038/sj.cdd.4401397)) |
| Rn.50333 | XM_223012 | Tp53bp2 | Tumor protein p53 binding protein, 2 | Trp53bp2 | 0.5152 | 0.671082 | [Tp53bp2](http://www.genecards.org/cgi-bin/carddisp.pl?gene=Tp53bp2) | Y. Samuels-Lev et al. 2001 ([DOI](http://dx.doi.org/10.1016/S1097-2765(01)00367-7)) |
| Rn.2411 | XM_342346 | Nfkb1 | Nuclear factor of kappa light polypeptide gene enhancer in B-cells 1 | NF-kB | 0.8971 | 0.675962 | [Nfkb1](http://www.genecards.org/cgi-bin/carddisp.pl?gene=Nfkb1) | M. Pizzi et al. 2009 ([DOI](http://dx.doi.org/10.1111/j.1742-4658.2008.06767.x)) |
| Rn.160577 | NM_080769 | Lta | Lymphotoxin alpha (TNF superfamily, member 1) | Tnfb | 2.1535 | 0.677997 | [Lta](http://www.genecards.org/cgi-bin/carddisp.pl?gene=Lta) | V. Upadhyay et al. 2013 ([DOI](http://dx.doi.org/10.1038/nri3406)) |
| Rn.92211 | NM_173340 | Rpl13a | Ribosomal protein L13A | - | 1.146 | 0.701604 | [Rpl13a](http://www.genecards.org/cgi-bin/carddisp.pl?gene=Rpl13a) | K. M. Curtis et al. 2010 ([DOI](http://dx.doi.org/10.1186/1471-2199-11-61)) |
| Rn.12033 | NM_001108724 | Traf3 | Tnf receptor-associated factor 3 | - | 1.0968 | 0.731067 | [Traf3](http://www.genecards.org/cgi-bin/carddisp.pl?gene=Traf3) | J. Q. He et al. 2006 ([DOI](http://dx.doi.org/10.1084/jem.20061166)) |
| Rn.91239 | NM_022231 | Xiap | X-linked inhibitor of apoptosis | Api3/ Birc4/ riap3 | 0.9548 | 0.73844 | [Xiap](http://www.genecards.org/cgi-bin/carddisp.pl?gene=Xiap) | R. W. Keane et al. 2001 ([DOI](http://dx.doi.org/10.1097/00004647-200110000-00007)) |
| Rn.89639 | NM_057130 | Hrk | Harakiri, BCL2 interacting protein (contains only BH3 domain) | Bid3/ Dp5 | 1.0401 | 0.78345 | [Hrk](http://www.genecards.org/cgi-bin/carddisp.pl?gene=Hrk) | S. Barrera-Vilarmau et al. 2011 ([DOI](http://dx.doi.org/10.1371/journal.pone.0021413)) |
| Rn.107896 | NM_017025 | Ldha | Lactate dehydrogenase A | Ldh1 | 1.0943 | 0.786915 | [Ldha](http://www.genecards.org/cgi-bin/carddisp.pl?gene=Ldha) | R. L. Büyükuysal 2005 ([DOI](http://dx.doi.org/10.1016/j.neuint.2005.06.009)) |
| Rn.54443 | NM_030989 | Tp53 | Tumor protein p53 | MGC112612/ Trp53/ p53 | 1.1487 | 0.802669 | [Tp53](http://www.genecards.org/cgi-bin/carddisp.pl?gene=Tp53) | C. Wan et al. 2013 ([DOI](http://dx.doi.org/10.1007/s12031-013-0050-4)) |
| Rn.13007 | NM_031328 | Bcl10 | B-cell CLL/ lymphoma 10 | - | 1.0619 | 0.864752 | [Bcl10](http://www.genecards.org/cgi-bin/carddisp.pl?gene=Bcl10) | R. Jürgen et al. 2001 ([DOI](http://dx.doi.org/10.1016/S0092-8674(01)00189-1)) |
| Rn.103860 | NM_001108696 | Tp73 | Tumor protein p73 | P73/ Trp73 | 0.9659 | 0.872976 | [Tp73](http://www.genecards.org/cgi-bin/carddisp.pl?gene=Tp73) | A. E. Sayan et al. 2008 ([DOI](http://dx.doi.org/10.1038/onc.2008.64)) |
| Rn.103083 | NM_001127379 | Api5 | Apoptosis inhibitor 5 | MGC187857 | 0.9885 | 0.873643 | [Api5](http://www.genecards.org/cgi-bin/carddisp.pl?gene=Api5) | M. Tewari et al. 1997 ([DOI](http://cancerres.aacrjournals.org/content/57/18/4063.long)) |
| Rn.1438 | NM_022522 | Casp2 | Caspase 2 | - | 1.1199 | 0.953025 | [Casp2](http://www.genecards.org/cgi-bin/carddisp.pl?gene=Casp2) | C. Tamm et al. 2008 ([DOI](http://dx.doi.org/10.1007/s10495-007-0172-7)) |

Data for all detectable genes (those genes that were flagged as undetectable or unreliable are not included) are arranged in descending order based on p values. Hyperlinks to the GeneCard entry for each gene and Digital Object Identifier (DOI) for at least one supporting published reference are included. GeneCards is a searchable, integrated, database of human genes that provides concise genomic related information, on all known and predicted human genes.
