# Supplementary material for: Pathway-Focused PCR Array Profiling of Enriched Populations of Laser Capture Microdissected Hippocampal Cells after Traumatic Brain Injury
Source: PLoS One. 2015 May 27;10(5):e0127287. doi: 10.1371/journal.pone.0127287 (PMC4446038; doi:10.1371/journal.pone.0127287)
Supplement: S2 Table — (DOCX) [file pone.0127287.s004.docx]

**Table S2. Neurotrophins and Receptors PCR Array.**Fold changes are shown as ratios of gene expression in dying versus surviving neurons.

| **Unigene** | **RefSeq** | **Symbol** | **Description** | **Gene Name** | **Fold Change** | **p-value** | **Gene**  **Card** | **PubMed Links** |
| --- | --- | --- | --- | --- | --- | --- | --- | --- |
| Rn.55036 | NM_019139 | Gdnf | Glial cell derived neurotrophic factor | - | 14.0367 | 0.011829 | [Gdnf](http://www.genecards.org/cgi-bin/carddisp.pl?gene=Gdnf) | Y. -.M Yoo et al.2012 ([DOI](http://dx.doi.org/10.1016/j.brainres.2012.09.006)); I. Kanter-Schlifke et al. 2009 ([DOI](http://dx.doi.org/10.1016/j.expneurol.2008.12.021)); J. E. Minnich et al. 2012 ([DOI](http://dx.doi.org/10.3233/RNN-2010-0528)); L. -F. Wong et al. 2006 ([DOI](http://dx.doi.org/10.1016/j.ymthe.2004.08.026)) |
| Rn.973 | NM_181091 | Gmfg | Glia maturation factor, gamma | - | 4.8159 | 0.017405 | [Gmfg](http://www.genecards.org/cgi-bin/carddisp.pl?gene=Gmfg) | H. Tsuiki et al. 2000 ([DOI](https://www.jstage.jst.go.jp/article/biochemistry1922/127/3/127_3_517/_pdf)) |
| Rn.24822 | NM_019172 | Galr2 | Galanin receptor 2 | - | 3.3122 | 0.025542 | [Galr2](http://www.genecards.org/cgi-bin/carddisp.pl?gene=Galr2) | R. Toifghi et al. 2008 ([DOI](http://dx.doi.org/10.1073/pnas.0712300105)); Y. Yang et al. 2006 ([DOI](http://dx.doi.org/10.1016/j.brainres.2005.12.060)) |
| Rn.137580 | NM_022205 | Cxcr4 | Chemokine (C-X-C motif) receptor 4 | MGC108696 | 29.8805 | 0.036531 | [Cxcr4](http://www.genecards.org/cgi-bin/carddisp.pl?gene=Cxcr4) | A. J. Shepherd et al. 2012 ([DOI](http://www.jneurosci.org/content/32/49/17725.full.pdf+html)); V. Ödemis et al. 2002 ([DOI](http://dx.doi.org/10.1074/jbc.M200472200)); X. Liu et al. 2013 ([DOI](http://dx.doi.org/10.1038/bonekey.2013.52)) |
| Rn.34398 | NM_030997 | Vgf | VGF nerve growth factor inducible | - | 2.0202 | 0.060712 | [Vgf](http://www.genecards.org/cgi-bin/carddisp.pl?gene=Vgf) | J. Adler et al. 2003 ([DOI](http://www.jneurosci.org/content/23/34/10800.full.pdf+html)); G. -L. Ferri et al. 2011 ([DOI](http://dx.doi.org/10.1016/j.jchemneu.2011.05.007)) |
| Rn.204252 | NM_022196 | Lif | Leukemia inhibitory factor | - | 11.6678 | 0.078427 | [Lif](http://www.genecards.org/cgi-bin/carddisp.pl?gene=Lif) | B. E. Deverman & P. H. Patterson et al. 2012 ([DOI](http://dx.doi.org/10.1523/JNEUROSCI.3803-11.2012)) |
| Rn.92460 | NM_173340 | Rpl13a | Ribosomal protein L13A | - | 1.775 | 0.085532 | [Rpl13a](http://www.genecards.org/cgi-bin/carddisp.pl?gene=Rpl13a) | K. M. Curtis et al. 2010 ([DOI](http://dx.doi.org/10.1186/1471-2199-11-61)) |
| Rn.22182 | NM_012583 | Hprt1 | Hypoxanthine phosphoribosyltrans-ferase 1 | Hgprtase/ Hprt/ MGC112554 | 0.6319 | 0.101294 | [Hprt1](http://www.genecards.org/cgi-bin/carddisp.pl?gene=Hprt1) | D. Sculley et al. 1992 ([DOI](http://dx.doi.org/10.1007/BF00220062)) |
| Rn.24402 | NM_001007604 | Rplp1 | Ribosomal protein, large, P1 | MGC72935 | 1.5416 | 0.1443 | [Rplp1](http://www.genecards.org/cgi-bin/carddisp.pl?gene=Rplp1) | S. C. Tan et al. 2012 ([DOI](http://link.springer.com/article/10.1007/s11033-011-1281-5)) |
| Rn.11642 | NM_012671 | Tgfa | Transforming growth factor alpha | RATTGFAA/ TGFAA | 7.9143 | 0.164729 | [Tgfa](http://www.genecards.org/cgi-bin/carddisp.pl?gene=Tgfa) | M. Guerra-Crespo et al. 2009 ([DOI](http://dx.doi.org/10.1016/j.neuroscience.2009.02.029)) |
| Rn.1772 | NM_017025 | Ldha | Lactate dehydrogenase A | Ldh1 | 0.5788 | 0.165148 | [Ldha](http://www.genecards.org/cgi-bin/carddisp.pl?gene=Ldha) | D. Martins-de-Souza et al. 2012 ([DOI](http://dx.doi.org/10.1021/pr2012279)) |
| Rn.11266 | NM_013014 | Pspn | Persephin | PSP | 5.1259 | 0.167325 | [Pspn](http://www.genecards.org/cgi-bin/carddisp.pl?gene=Pspn) | J. Milbrandt et al. 1998 ([DOI](http://dx.doi.org/10.1016/S0896-6273(00)80453-5)) |
| Rn.47 | NM_133534 | Cx3cr1 | Chemokine (C-X3-C motif) receptor 1 | Rbs11 | 7.6447 | 0.179383 | [Cx3cr1](http://www.genecards.org/cgi-bin/carddisp.pl?gene=Cx3cr1) | M. Zhang et al. 2012 ([DOI](http://dx.doi.org/10.1371/journal.pone.0035446)) |
| Rn.9873 | NM_031032 | Gmfb | Glia maturation factor, beta | MGC93372 | 0.6899 | 0.190486 | [Gmfb](http://www.genecards.org/cgi-bin/carddisp.pl?gene=Gmfb) | R. Thangavel et al. 2012 ([DOI](http://onlinelibrary.wiley.com/doi/10.1111/j.1365-2990.2011.01232.x/pdf)) |
| Rn.12072 | XM_344662 | Nrg2 | Neuregulin 2 | NTAK_alpha2a/ Ntak | 38.795 | 0.22268 | [Nrg2](http://www.genecards.org/cgi-bin/carddisp.pl?gene=Nrg2) | M. Longart et al. 2004 ([DOI](http://onlinelibrary.wiley.com/doi/10.1002/cne.20016/pdf)) |
| Rn.10686 | NM_053397 | Artn | Artemin | - | 3.1191 | 0.225643 | [Artn](http://www.genecards.org/cgi-bin/carddisp.pl?gene=Artn) | R. H. Baloh et al. 1998 ([DOI](http://dx.doi.org/10.1016/S0896-6273(00)80649-2)) |
| Rn.10980 | NM_012589 | Il6 | Interleukin 6 | ILg6/ Ifnb2 | 3.6582 | 0.22893 | [Il6](http://www.genecards.org/cgi-bin/carddisp.pl?gene=Il6) | X. -H. Wei et al. 2013 ([DOI](http://dx.doi.org/10.1016/j.expneurol.2012.12.007)) |
| Rn.9714 | NM_031970 | Hspb1 | Heat shock protein 1 | Hsp25/ Hsp27 | 3.2742 | 0.231898 | [Hspb1](http://www.genecards.org/cgi-bin/carddisp.pl?gene=Hspb1) | S. Hu et al. 2009 ([DOI](http://dx.doi.org/10.1016/j.brainres.2008.10.054)) |
| Rn.89609 | NM_134360 | Cd40 | CD40 molecule, TNF receptor superfamily member 5 | Tnfrsf5 | 8.1934 | 0.23266 | [Cd40](http://www.genecards.org/cgi-bin/carddisp.pl?gene=Cd40) | N. Y. Calingasan et al. 2002 ([DOI](http://dx.doi.org/10.1016/S0197-4580(01)00246-9)) |
| Rn.9952 | NM_019305 | Fgf2 | Fibroblast growth factor 2 | Fgf-2/ bFGF | 2.7091 | 0.241352 | [Fgf2](http://www.genecards.org/cgi-bin/carddisp.pl?gene=Fgf2) | F. Gaughran et al. 2006 ([DOI](http://dx.doi.org/10.1016/j.brainresbull.2006.04.008)) |
| Rn.10184 | XM_227525 | Ngf | Nerve growth factor (beta polypeptide) | Ngfb | 2.2467 | 0.25445 | [Ngf](http://www.genecards.org/cgi-bin/carddisp.pl?gene=Ngf) | M. F. Philips et al. 2001 ([DOI](http://thejns.org/doi/abs/10.3171/jns.2001.94.5.0765)) |
| Rn.10316 | NM_030989 | Tp53 | Tumor protein p53 | MGC112612/ Trp53/ p53 | 2.0202 | 0.255341 | [Tp53](http://www.genecards.org/cgi-bin/carddisp.pl?gene=Tp53) | S. Medrano et al. 2009 ([DOI](http://dx.doi.org/10.1016/j.neurobiolaging.2007.07.016)) |
| Rn.11246 | NM_022197 | Fos | FBJ osteosarcoma oncogene | c-fos | 3.8937 | 0.260306 | [Fos](http://www.genecards.org/cgi-bin/carddisp.pl?gene=Fos) | K. Nakadate et al. 2013 ([DOI](http://dx.doi.org/10.1016/j.neuroscience.2013.01.013)) |
| Rn.10247 | NM_001012137 | Fus | Fusion (involved in t(12;16) in malignant liposarcoma) (human) | - | 1.4754 | 0.265795 | [Fus](http://www.genecards.org/cgi-bin/carddisp.pl?gene=Fus) | C. Huang et al. 2010 ([DOI](http://dx.doi.org/10.7150/ijbs.6.396)) |
| Rn.90137 | NM_031581 | Ppyr1 | Pancreatic polypeptide receptor 1 | - | 6.0817 | 0.269137 | [Ppyr1](http://www.genecards.org/cgi-bin/carddisp.pl?gene=Ppyr1) | A. Yahya et al. 2006 ([DOI](http://dx.doi.org/10.1016/j.peptides.2006.07.003)) |
| Rn.9868 | NM_013184 | Ntf4 | Neurotrophin 4 | NT4P/ Ntf5 | 4.9857 | 0.270765 | [Ntf4](http://www.genecards.org/cgi-bin/carddisp.pl?gene=Ntf4) | T. Oshitari et al. 2011 ([DOI](http://dx.doi.org/10.1016/j.neulet.2011.06.057)) |
| Rn.25174 | NM_031088 | Ptger2 | Prostaglandin E receptor 2 (subtype EP2) | EP2/ Ptger-ep2 | 9.8341 | 0.271072 | [Ptger2](http://www.genecards.org/cgi-bin/carddisp.pl?gene=Ptger2) | Y. Quan et al. 2013 ([DOI](http://www.jbc.org/content/288/13/9293.long)) |
| Rn.10213 | NM_030999 | Crhr1 | Corticotropin releasing hormone receptor 1 | - | 2.917 | 0.272741 | [Crhr1](http://www.genecards.org/cgi-bin/carddisp.pl?gene=Crhr1) | H. A. Dunn et al. 2013 ([DOI](http://dx.doi.org/10.1074/jbc.M113.473660)) |
| Rn.88489 | NM_139194 | Fas | Fas (TNF receptor superfamily, member 6) | Tnfrsf6 | 1.7668 | 0.275026 | [Fas](http://www.genecards.org/cgi-bin/carddisp.pl?gene=Fas) | J. Yang et al. 2010 ([DOI](http://online.liebertpub.com/doi/pdf/10.1089/neu.2009.1229)) |
| Rn.44349 | NM_012603 | Myc | Myelocytomatosis oncogene | MGC105490/ RNCMYC/ c-myc/ mMyc | 6.2671 | 0.276859 | [Myc](http://www.genecards.org/cgi-bin/carddisp.pl?gene=Myc) | J. T. Babcock et al. 2013 ([DOI](http://dx.doi.org/10.1074/jbc.M112.431056)) |
| Rn.88262 | NM_012609 | Nf1 | Neurofibromin 1 | - | 1.596 | 0.28152 | [Nf1](http://www.genecards.org/cgi-bin/carddisp.pl?gene=Nf1) | H. -F. Wang et al. 2011 ([DOI](http://dx.doi.org/10.1172/JCI45677)) |
| Rn.9893 | NM_012667 | Tacr1 | Tachykinin receptor 1 | Tac1r | 6.3988 | 0.286024 | [Tacr1](http://www.genecards.org/cgi-bin/carddisp.pl?gene=Tacr1) | N. Pantaleo et al. 2010 ([DOI](http://www.ncbi.nlm.nih.gov/pmc/articles/PMC2967650/pdf/nihms223084.pdf)) |
| Rn.9758 | NM_001113357 | Npy1r | Neuropeptide Y receptor Y1 | MGC109393/ NPY-1 | 2.9783 | 0.287219 | [Npy1r](http://www.genecards.org/cgi-bin/carddisp.pl?gene=Npy1r) | Y. -S. Hsieh et al. 2013 ([DOI](http://dx.doi.org/10.1007/s12017-012-8206-x)) |
| Rn.6067 | NM_031048 | Lifr | Leukemia inhibitory factor receptor alpha | - | 1.9832 | 0.287863 | [Lifr](http://www.genecards.org/cgi-bin/carddisp.pl?gene=Lifr) | C. Nogueira-Silva et al. 2012 ([DOI](http://dx.doi.org/10.1371/journal.pone.0030517)) |
| Rn.7628 | NM_001109127 | Cbln1 | Cerebellin 1 precursor | - | 5.4811 | 0.287921 | [Cbln1](http://www.genecards.org/cgi-bin/carddisp.pl?gene=Cbln1) | M. Rucinski et al . 2009 ([DOI](http://www.spandidos-publications.com/ijmm/23/3/363)) |
| Rn.44225 | NM_053968 | Mt3 | Metallothionein 3 | GIF/ Mt-3 | 2.3693 | 0.288318 | [Mt3](http://www.genecards.org/cgi-bin/carddisp.pl?gene=Mt3) | J. He et al. 2013 ([DOI](http://dx.doi.org/10.1007/s10072-012-0978-0)) |
| Rn.9996 | NM_001024775 | Zfp110 | Zinc finger protein 110 | Nrif1 | 2.6289 | 0.291013 | [Zfp110](http://www.genecards.org/cgi-bin/carddisp.pl?gene=Zfp110) | F. S. Collins 2002 ([DOI](http://dx.doi.org/10.1073/pnas.242603899)) |
| Rn.1716 | NM_013179 | HcRt | Hypocretin | orexin-A | 39.6102 | 0.293383 | [HcRt](http://www.genecards.org/cgi-bin/carddisp.pl?gene=HcRt) | T. A. Butterick et al. 2012 ([DOI](http://dx.doi.org/10.1016/j.neulet.2012.07.002)) |
| Rn.107896 | NM_001169120 | Zfp91 | Zinc finger protein 91 | - | 1.7465 | 0.298142 | [Zfp91](http://www.genecards.org/cgi-bin/carddisp.pl?gene=Zfp91) | V. Di Pietro et al. 2013 ([DOI](http://link.springer.com/article/10.1007/s11010-012-1541-2)) |
| Rn.10668 | NM_133511 | Adcyap1r1 | Adenylate cyclase activating polypeptide 1 receptor 1 | PACAP-R1A/ PACAPR1/ PACAPR1A | 4.6197 | 0.299837 | [Adcyap1r1](http://www.genecards.org/cgi-bin/carddisp.pl?gene=Adcyap1r1) | A. U. Syed et al . 2012 ([DOI](http://dx.doi.org/10.1007/s12031-012-9851-0)) |
| Rn.53970 | NM_001012226 | Stat4 | Signal transducer and activator of transcription 4 | - | 2.9714 | 0.300263 | [Stat4](http://www.genecards.org/cgi-bin/carddisp.pl?gene=Stat4) | K. Robinson et al. 2006 ([DOI](http://www.sciencedirect.com/science/article/pii/S1072751506000457)) |
| Rn.10190 | NM_021578 | Tgfb1 | Transforming growth factor, beta 1 | - | 6.8739 | 0.300853 | [Tgfb1](http://www.genecards.org/cgi-bin/carddisp.pl?gene=Tgfb1) | R. Vivar et al. 2013 ([DOI](http://dx.doi.org/10.1016/j.bbadis.2013.02.004)) |
| Rn.9789 | NM_013123 | Il1r1 | Interleukin 1 receptor, type I | - | 7.1493 | 0.306963 | [Il1r1](http://www.genecards.org/cgi-bin/carddisp.pl?gene=Il1r1) | S. Spulber et al. 2009 ([DOI](http://dx.doi.org/10.1016/j.jneuroim.2009.01.010)) |
| Rn.31808 | NM_001100491 | Mc2r | Melanocortin 2 receptor | - | 15.3248 | 0.310411 | [Mc2r](http://www.genecards.org/cgi-bin/carddisp.pl?gene=Mc2r) | I. A. Malik et al. 2012 ([DOI](http://dx.doi.org/10.1007/s00418-011-0899-7)) |
| Rn.40136 | NM_012706 | Grpr | Gastrin releasing peptide receptor | - | 4.7826 | 0.325695 | [Grpr](http://www.genecards.org/cgi-bin/carddisp.pl?gene=Grpr) | J. Presti-Torres et al. 2007 ([DOI](http://dx.doi.org/10.1016/j.neuropharm.2006.09.020)) |
| Rn.39098 | NM_053398 | Gfra3 | GDNF family receptor alpha 3 | - | 8.9041 | 0.330388 | [Gfra3](http://www.genecards.org/cgi-bin/carddisp.pl?gene=Gfra3) | S. L. Forrest & J. R. Keast et al. 2008 ([DOI](http://onlinelibrary.wiley.com/doi/10.1002/cne.21535/pdf)) |
| Rn.44379 | NM_001003929 | Cntfr | Ciliary neurotrophic factor receptor | - | 2.4135 | 0.332817 | [Cntfr](http://www.genecards.org/cgi-bin/carddisp.pl?gene=Cntfr) | N. Lee et al. 2013 ([DOI](http://dx.doi.org/10.1523/JNEUROSCI.3386-12.2013)) |
| Rn.103750 | NM_017020 | Il6r | Interleukin 6 receptor | IL6R1/ Il6ra | 1.8589 | 0.338919 | [Il6r](http://www.genecards.org/cgi-bin/carddisp.pl?gene=Il6r) | M. Mihara et al. 2012 ([DOI](http://dx.doi.org/10.1042/CS20110340)) |
| Rn.44431 | NM_001008725 | Il6st | Interleukin 6 signal transducer | Ac1055/ Gp130/ Il-6rb | 2.4303 | 0.342286 | [Il6st](http://www.genecards.org/cgi-bin/carddisp.pl?gene=Il6st) | S. P. Lam et al. 2010 ([DOI](http://dx.doi.org/10.1002/lt.22136)) |
| Rn.10023 | XR_006259 | LOC685671 | Similar to myocyte enhancer factor 2C | - | 2.4642 | 0.346806 | Not located | V. G. Dmitrieva et al. 2008 ([DOI](http://link.springer.com/article/10.1134%2FS1607672908050037?LI=true)) |
| Rn.64505 | NM_012610 | Ngfr | Nerve growth factor receptor (TNFR superfamily, member 16) | LNGFR/ RNNGFRR/ p75/ p75NTR | 17.6443 | 0.347529 | [Ngfr](http://www.genecards.org/cgi-bin/carddisp.pl?gene=Ngfr) | F. Montazeri et al. 2013 ([DOI](http://dx.doi.org/10.1179/2045772312Y.0000000077)) |
| Rn.64524 | NM_012750 | Gfra2 | GDNF family receptor alpha 2 | Retl2 | 8.7612 | 0.34959 | [Gfra2](http://www.genecards.org/cgi-bin/carddisp.pl?gene=Gfra2) | T. Kawakami et al. 2003 ([DOI](http://dx.doi.org/10.1002/nau.10074)) |
| Rn.9797 | NM_139183 | Crhbp | Corticotropin releasing hormone binding protein | Crfbp | 2.2781 | 0.350287 | [Crhbp](http://www.genecards.org/cgi-bin/carddisp.pl?gene=Crhbp) | A. -M. Buga et al. 2012 ([DOI](http://www.plosone.org/article/info%3Adoi%2F10.1371%2Fjournal.pone.0050985)) |
| Rn.54443 | NM_057193 | Il10ra | Interleukin 10 receptor, alpha | - | 2.3099 | 0.352689 | [Il10ra](http://www.genecards.org/cgi-bin/carddisp.pl?gene=Il10ra) | K. Kamm et al. 2006 ([DOI](http://dx.doi.org/10.1097/01.ta.0000196345.81169.a1)) |
| Rn.9704 | NM_016993 | Bcl2 | B-cell CLL/ lymphoma 2 | Bcl-2 | 2.3045 | 0.35529 | [Bcl2](http://www.genecards.org/cgi-bin/carddisp.pl?gene=Bcl2) | N. Z. Hoh et al. 2010 ([DOI](http://dx.doi.org/10.1089/neu.2009.1256)) |
| Rn.10499 | NM_012854 | Il10 | Interleukin 10 | IL10X | 20.7418 | 0.361909 | [Il10](http://www.genecards.org/cgi-bin/carddisp.pl?gene=Il10) | A. Y. Lai et al. 2006 ([DOI](http://dx.doi.org/10.1016/j.jneuroim.2006.03.001)) |
| Rn.10349 | NM_013166 | Cntf | Ciliary neurotrophic factor | - | 2.2939 | 0.364864 | [Cntf](http://www.genecards.org/cgi-bin/carddisp.pl?gene=Cntf) | M. K. Paintlia et al. 2012 ([DOI](http://dx.doi.org/10.1074/jbc.M112.405654)) |
| Rn.10454 | NM_012614 | Npy | Neuropeptide Y | NPY02/ RATNPY/ RATNPY02 | 2.8768 | 0.365288 | [Npy](http://www.genecards.org/cgi-bin/carddisp.pl?gene=Npy) | P. K. Dash et al. 2010 ([DOI](http://dx.doi.org/10.1016/j.nurt.2009.10.019)) |
| Rn.14529 | NM_052980 | Nr1i2 | Nuclear receptor subfamily 1, group I, member 2 | MGC108643/ PXR | 7.4014 | 0.370205 | [Nr1i2](http://www.genecards.org/cgi-bin/carddisp.pl?gene=Nr1i2) | C. -C. Hung et al. 2007 ([DOI](http://dx.doi.org/10.2217/14622416.8.9.1151)) |
| Rn.10695 | XM_341934 | Tgfb1i1 | Transforming growth factor beta 1 induced transcript 1 | Ara55/ Hic-5 | 2.2005 | 0.374339 | [Tgfb1i1](http://www.genecards.org/cgi-bin/carddisp.pl?gene=Tgfb1i1) | S. Inui et al. 2012 ([DOI](http://dx.doi.org/10.1016/j.jdermsci.2012.09.007)) |
| Rn.9715 | NM_022714 | Crhr2 | Corticotropin releasing hormone receptor 2 | Crf2r | 5.9154 | 0.378437 | [Crhr2](http://www.genecards.org/cgi-bin/carddisp.pl?gene=Crhr2) | D. K. Grammatopoulos and G. P. Chrousos 2002 ([DOI](http://dx.doi.org/10.1016/S1043-2760(02)00670-7)) |
| Rn.10264 | NM_017059 | Bax | Bcl2-associated X protein | - | 1.9423 | 0.388732 | [Bax](http://www.genecards.org/cgi-bin/carddisp.pl?gene=Bax) | A. Iwata et al. 2010 ([DOI](http://www.plosone.org/article/info%3Adoi%2F10.1371%2Fjournal.pone.0009103)) |
| Rn.94978 | NM_012513 | Bdnf | Brain-derived neurotrophic factor | MGC105254 | 0.5192 | 0.39019 | [Bdnf](http://www.genecards.org/cgi-bin/carddisp.pl?gene=Bdnf) | W. W. Poon et al. 2013 ([DOI](http://dx.doi.org/10.1074/jbc.M113.463711)); Z. Li et al. 2013 ([DOI](http://dx.doi.org/10.1111/jnc.12249)) |
| Rn.9869 | NM_031588 | Nrg1 | Neuregulin 1 | - | 4.9284 | 0.396303 | [Nrg1](http://www.genecards.org/cgi-bin/carddisp.pl?gene=Nrg1) | Chongchong Xu et al. 2012 ([DOI](http://dx.doi.org/10.1016/j.brainres.2012.05.044)); W. -P. Guo et al. 2006 ([DOI](http://dx.doi.org/10.1016/j.brainres.2006.03.007)) |
| Rn.10574 | NM_012958 | Galr1 | Galanin receptor 1 | Galnr1 | 2.6595 | 0.397276 | [Galr1](http://www.genecards.org/cgi-bin/carddisp.pl?gene=Galr1) | L. Holm et al. 2012 ([DOI](http://dx.doi.org/10.1016/j.npep.2011.11.001)) |
| Rn.37438 | NM_012688 | Cckar | Cholecystokinin A receptor | Cck-ar | 1.9877 | 0.397428 | [Cckar](http://www.genecards.org/cgi-bin/carddisp.pl?gene=Cckar) | P. Petkova-Kirova et al. 2012 ([DOI](http://dx.doi.org/10.1016/j.brainresbull.2012.08.009)) |
| Rn.3841 | NM_001011905 | Stat2 | Signal transducer and activator of transcription 2 | - | 1.4186 | 0.421666 | [Stat2](http://www.genecards.org/cgi-bin/carddisp.pl?gene=Stat2) | W. L. Hsu et al. 2009 ([DOI](http://dx.doi.org/10.1038/cdd.2009.91)) |
| Rn.33229 | NM_013064 | Hcrtr1 | Hypocretin (orexin) receptor 1 | Hctr1 | 1.4619 | 0.432431 | [Hcrtr1](http://www.genecards.org/cgi-bin/carddisp.pl?gene=Hcrtr1) | R. Fronczek et al. 2009 ([DOI](http://dx.doi.org/10.1016/j.smrv.2008.05.002)) |
| Rn.207164 | NM_012952 | Fgf9 | Fibroblast growth factor 9 | - | 1.4551 | 0.442479 | [Fgf9](http://www.genecards.org/cgi-bin/carddisp.pl?gene=Fgf9) | J. -Y. Huanga & J. -I. Chuang 2010 ([DOI](http://dx.doi.org/10.1016/j.freeradbiomed.2010.06.026)) |
| Rn.154711 | NM_053401 | Ngfrap1 | Nerve growth factor receptor (TNFRSF16) associated protein 1 | Bex3/ Nade | 1.5416 | 0.444715 | [Ngfrap1](http://www.genecards.org/cgi-bin/carddisp.pl?gene=Ngfrap1) | T. A. Slotkin et al. 2008 ([DOI](http://dx.doi.org/10.1016/j.brainresbull.2008.01.001)) |
| Rn.15499 | NM_031073 | Ntf3 | Neurotrophin 3 | - | 1.3926 | 0.456925 | [Ntf3](http://www.genecards.org/cgi-bin/carddisp.pl?gene=Ntf3) | K. I. Park et al. 2006 ([DOI](http://dx.doi.org/10.1016/j.expneurol.2006.03.016)) |
| Rn.3126 | NM_031019 | Crh | Corticotropin releasing hormone | CRF | 1.1738 | 0.461046 | [Crh](http://www.genecards.org/cgi-bin/carddisp.pl?gene=Crh) | F. Tanriverdi et al. 2008 ([DOI](http://dx.doi.org/10.1530/EJE-08-0050)) |
| Rn.54596 | NM_001017382 | Frs3 | Fibroblast growth factor receptor substrate 3 | - | 1.3703 | 0.46208 | [Frs3](http://www.genecards.org/cgi-bin/carddisp.pl?gene=Frs3) | D. Sun et al. 2009 ([DOI](http://dx.doi.org/10.1016/j.expneurol.2008.11.011)) |
| Rn.11325 | NM_001012144 | Tfg | Trk-fused gene | - | 1.4823 | 0.490621 | [Tfg](http://www.genecards.org/cgi-bin/carddisp.pl?gene=Tfg) | R. Qu et al. 2007 ([DOI](http://dx.doi.org/10.1111/j.1440-1789.2007.00792.x)) |
| Rn.64632 | NM_031069 | Nell1 | NEL-like 1 (chicken) | - | 1.2756 | 0.514189 | [Nell1](http://www.genecards.org/cgi-bin/carddisp.pl?gene=Nell1) | K. -H. Lee et al. 2009 ([DOI](http://dx.doi.org/10.1007/s11064-008-9867-6)) |
| Rn.88408 | NM_032612 | Stat1 | Signal transducer and activator of transcription 1 | - | 1.1002 | 0.527117 | [Stat1](http://www.genecards.org/cgi-bin/carddisp.pl?gene=Stat1) | G. Wang et al. 2012 ([DOI](http://dx.doi.org/10.1186/1742-2094-9-170)) |
| Rn.10482 | NM_012731 | Ntrk2 | Neurotrophic tyrosine kinase, receptor, type 2 | RATTRKB1/ TRKB1/ Tkrb/ trkB | 0.7816 | 0.535421 | [Ntrk2](http://www.genecards.org/cgi-bin/carddisp.pl?gene=Ntrk2) | D. Sun et al. 2007 ([DOI](http://www.nature.com/npp/journal/v32/n1/abs/1301134a.html)) |
| Rn.25180 | NM_012747 | Stat3 | Signal transducer and activator of transcription 3 | MGC93551 | 2.1702 | 0.548485 | [Stat3](http://www.genecards.org/cgi-bin/carddisp.pl?gene=Stat3) | S. Bokua et al. 2013 ([DOI](http://dx.doi.org/10.1016/j.bbrc.2013.04.011)) |
| Rn.34568 | NM_023980 | Npffr2 | Neuropeptide FF receptor 2 | Gpr74/ Npff2/ Npgpr | 2.2781 | 0.561718 | [Npffr2](http://www.genecards.org/cgi-bin/carddisp.pl?gene=Npffr2) | A. K. Smith et al. 2011 ([DOI](http://onlinelibrary.wiley.com/doi/10.1002/ajmg.b.31212/full)) |
| Rn.162521 | NM_012959 | Gfra1 | GDNF family receptor alpha 1 | - | 1.0578 | 0.577119 | [Gfra1](http://www.genecards.org/cgi-bin/carddisp.pl?gene=Gfra1) | N. Kholodilov et al. 2011 ([DOI](http://dx.doi.org/10.1111/j.1471-4159.2010.07128.x)) |
| Rn.92211 | NM_001108097 | Frs2 | Fibroblast growth factor receptor substrate 2 | - | 1.0361 | 0.683488 | [Frs2](http://www.genecards.org/cgi-bin/carddisp.pl?gene=Frs2) | W. E. Leadbeater et al. 2006 ([DOI](http://dx.doi.org/10.1111/j.1471-4159.2005.03632.x)) |
| Rn.15333 | NM_024146 | Fgfr1 | Fibroblast growth factor receptor 1 | - | 0.9316 | 0.715398 | [Fgfr1](http://www.genecards.org/cgi-bin/carddisp.pl?gene=Fgfr1) | M. E. Woodbury & T. Ikezu 2013 ([DOI](http://link.springer.com/article/10.1007%2Fs11481-013-9501-5)) |
| Rn.22168 | NM_023968 | Npy2r | Neuropeptide Y receptor Y2 | - | 0.9103 | 0.797693 | [Npy2r](http://www.genecards.org/cgi-bin/carddisp.pl?gene=Npy2r) | G. C. Mitchell et al. 2008 ([DOI](http://www.jneurosci.org/content/28/53/14428)) |
| Rn.103260 | NM_013074 | Hcrtr2 | Hypocretin (orexin) receptor 2 | - | 0.923 | 0.84658 | [Hcrtr2](http://www.genecards.org/cgi-bin/carddisp.pl?gene=Hcrtr2) | L. Chen et al. 2013 ([DOI](http://dx.doi.org/10.1111/ejn.12101)) |
| Rn.22434 | NM_031144 | Actb | Actin, beta | Actx | 1.0077 | 0.909438 | [Actb](http://www.genecards.org/cgi-bin/carddisp.pl?gene=Actb) | J. -H. Yi et al. 2008 ([DOI](http://dx.doi.org/10.1016/j.brainres.2008.09.074)) |
| Rn.110468 | NM_053409 | Maged1 | Melanoma antigen, family D, 1 | MGC93306/ Nrage | 7.9143 | 0.962139 | [Maged1](http://www.genecards.org/cgi-bin/carddisp.pl?gene=Maged1) | A. H. Salehi et al. 2000 ([DOI](http://dx.doi.org/10.1016/S0896-6273(00)00036-2)) |

Data for all detectable genes (those genes that were flagged as undetectable or unreliable are not included) are arranged in descending order based on p values. Hyperlinks to the GeneCard entry for each gene and Digital Object Identifier (DOI) for at least one supporting published reference are included. GeneCards is a searchable, integrated, database of human genes that provides concise genomic related information, on all known and predicted human genes.
