# Supplementary material for: Pathway-Focused PCR Array Profiling of Enriched Populations of Laser Capture Microdissected Hippocampal Cells after Traumatic Brain Injury
Source: PLoS One. 2015 May 27;10(5):e0127287. doi: 10.1371/journal.pone.0127287 (PMC4446038; doi:10.1371/journal.pone.0127287)
Supplement: S3 Table — (DOCX) [file pone.0127287.s005.docx]

| **Table S3. Apoptosis array CT values** | | | | | | | **Neurotrophin and Receptors array CT values** | | | | | | |
| --- | --- | --- | --- | --- | --- | --- | --- | --- | --- | --- | --- | --- | --- |
| **Well** | **Uninjured-1** | **Uninjured-2** | **Uninjured - 3** | **Injured-1** | **Injured-2** | **Injured-3** | **Well** | **Uninjured 1** | **Uninjured 2** | **Uninjured 3** | **Injured 1** | **Injured 2** | **Injured 3** |
| A1 | 25.65 | 27.08 | 25.85 | 30.31 | 28.03 | 28.62 | A1 | 26.13 | 28.65 | 28.06 | 25 | 27.2 | 29.46 |
| A2 | 21.34 | 21.59 | 21.23 | 24.56 | 22.87 | 23.55 | A2 | 25.96 | 26.7 | 27.75 | 25.55 | 26.73 | 28.65 |
| A3 | 29.22 | 30.46 | 31.67 | No Ct | 32.47 | 33.21 | A3 | 23.22 | 24.64 | 24.14 | 22.7 | 25.54 | 26.33 |
| A4 | 25.83 | 27.52 | 26.65 | 29.59 | 29.07 | 29.76 | A4 | 24.43 | 25.7 | 25.98 | 23.96 | 26.52 | 27.46 |
| A5 | 22.04 | 21.69 | 21.64 | 23.28 | 21.54 | 23.07 | A5 | 30.45 | 31.52 | 32.39 | 32.83 | 34.83 | 34.98 |
| A6 | 25.18 | 26.68 | 25.93 | 27.76 | 26.59 | 27.46 | A6 | 29.15 | 30.8 | 29.25 | 27.25 | 32.58 | 31.84 |
| A7 | 23.41 | 23.98 | 24.09 | 26.45 | 25.06 | 25.66 | A7 | 23.51 | 25.27 | 25.39 | 23.3 | 25.77 | 26.95 |
| A8 | 24.64 | 26.02 | 25.3 | 28.22 | 26.87 | 27.38 | A8 | 21.13 | 22.17 | 22.83 | 20.75 | 22.87 | 24.14 |
| A9 | 25.2 | 26.23 | 25.37 | 27.82 | 26.21 | 27.11 | A9 | 25.92 | 27.29 | 26.34 | 25.71 | 27.55 | 31.04 |
| A10 | 27.05 | 26.26 | 26.94 | 27.85 | 25.77 | 26.73 | A10 | 25.5 | 26.37 | 27.07 | 25.02 | 27.44 | 28.36 |
| A11 | 26.23 | 28.18 | 26.94 | 29.28 | 29.09 | 27.97 | A11 | 23.87 | 25.54 | 25.63 | 23.69 | 25.67 | 26.49 |
| A12 | No Ct | No Ct | No Ct | No Ct | 38.81 | No Ct | A12 | 28 | 29.21 | No Ct | 24.91 | 30.05 | No Ct |
| B1 | 21.37 | 21.75 | 22.02 | 24.68 | 22.41 | 24.28 | B1 | 27.38 | 29.42 | 29.5 | 26.14 | 28.06 | 28.74 |
| B2 | 20.56 | 20.09 | 20.36 | 22.87 | 21.28 | 22.8 | B2 | 31.39 | 34.07 | 33.06 | 28.91 | 30.06 | 30.29 |
| B3 | 23.18 | 22.79 | 22.74 | 25.62 | 23.71 | 25.5 | B3 | 31.2 | 32.72 | 31.14 | 29.65 | No Ct | 31.54 |
| B4 | 30 | 30.91 | 31.2 | 34.64 | 31.74 | 32.33 | B4 | 23.31 | 24.83 | 23.98 | 23.82 | 25.68 | 26.44 |
| B5 | 30.45 | 29.15 | 31.28 | 28.17 | 29.32 | 31.5 | B5 | 20.76 | 23.01 | 22.65 | 23.63 | 23.9 | 24.64 |
| B6 | 26.8 | 27.59 | 28.63 | 28.36 | 27 | 29.36 | B6 | 21.39 | 22.13 | 23.67 | 20.54 | 22.29 | 23.92 |
| B7 | 30.93 | 34.09 | No Ct | 32.56 | No Ct | No Ct | B7 | 27.98 | 29.08 | 28.37 | 31.02 | 30.24 | 29.46 |
| B8 | 24.17 | 26.46 | 25.3 | 28.71 | 27.07 | 27.12 | B8 | 29.05 | 30.47 | 29.73 | 28.67 | 31.56 | 33.1 |
| B9 | 25.08 | 23.3 | 24.48 | 26.63 | 24.42 | 26.45 | B9 | 24.37 | 24.8 | 24.88 | 25.17 | 26.1 | 26.54 |
| B10 | 23.4 | 24.54 | 23.59 | 26.79 | 26.02 | 26.81 | B10 | 27.28 | 30.75 | 30.52 | 25.87 | 32.25 | 31.64 |
| B11 | 24.24 | 24.84 | 25.39 | 26.92 | 25.63 | 26.42 | B11 | 34.74 | 35.55 | 35.75 | 36.34 | 39.79 | 38.61 |
| B12 | 22.02 | 23.99 | 23.01 | 26.64 | 25.55 | 25.27 | B12 | No Ct | No Ct | No Ct | 32.57 | 32.21 | 34.23 |
| C1 | 21.46 | 22.25 | 22.29 | 25.63 | 23.48 | 24.99 | C1 | 25.15 | 27.17 | 26.1 | 25.63 | 28.37 | 29.62 |
| C2 | 26.19 | 25.27 | 27.44 | 28.89 | 25.66 | 27.5 | C2 | 25.49 | 27.54 | 27.7 | 22.24 | 26.71 | 27.83 |
| C3 | 29.05 | 31.57 | 30.54 | 33.82 | 32.23 | 32.76 | C3 | 29.87 | 34.65 | 32.8 | 27.72 | 30.58 | No Ct |
| C4 | 32.47 | 33 | 32.49 | 35.39 | 31.59 | 32.41 | C4 | 18.2 | 19.06 | 19.79 | 21.56 | 20.51 | 22.03 |
| C5 | 26.35 | 27.05 | 26.82 | 28.01 | 27.24 | 28.26 | C5 | 24.6 | 25.02 | 25.91 | 24.16 | 24.45 | 25.56 |
| C6 | 28.21 | 27.61 | 28.42 | 28.08 | 26.86 | 27.45 | C6 | No Ct | 33.21 | 31.61 | 33.43 | 33.27 | No Ct |
| C7 | No Ct | No Ct | No Ct | No Ct | No Ct | No Ct | C7 | 33.39 | No Ct | 33.7 | 30.97 | 35.05 | 34.79 |
| C8 | 25.26 | 27.56 | 27.06 | 29.06 | 28.15 | 28.95 | C8 | No Ct | 33.83 | 34.59 | 27.73 | 30.21 | No Ct |
| C9 | 24.24 | 24.4 | 24.52 | 26.42 | 24.71 | 25.63 | C9 | 27.61 | 29.03 | 29.21 | 28.62 | 29.96 | 31.07 |
| C10 | 30.54 | 31.05 | 31.7 | 32.5 | 30.14 | No Ct | C10 | 23.6 | 24.47 | 25.52 | 25.05 | 26.09 | 28.24 |
| C11 | 35.97 | 34.62 | 33.42 | 31.99 | 34.13 | 34.95 | C11 | 18.57 | 19.34 | 20.08 | 17.96 | 19.75 | 20.59 |
| C12 | 27.68 | 30.75 | 30.09 | 30.23 | 29.82 | 30.66 | C12 | 33.16 | 30.51 | 36.24 | 25.58 | 30.41 | No Ct |
| D1 | 31.66 | 33.02 | 34.79 | 38.8 | 33.99 | 33.5 | D1 | 32.21 | 32.57 | 33.22 | No Ct | 33.21 | 31.61 |
| D2 | 23.96 | 24.24 | 24.91 | 28.77 | 25.94 | 26.96 | D2 | No Ct | No Ct | No Ct | 35.24 | No Ct | 35.47 |
| D3 | 25.49 | 26.41 | 25.68 | 28.27 | 27.26 | 27.5 | D3 | 29 | 31.64 | 29.8 | 26.58 | 30.55 | 30.24 |
| D4 | 28.23 | 28.82 | No Ct | 28.76 | 26.44 | 29.5 | D4 | 29.73 | 30.66 | 31.06 | 28.87 | 31.01 | 31.4 |
| D5 | 33.68 | 34.58 | 34.59 | No Ct | 36.12 | 33.99 | D5 | 27.57 | 29.02 | 28.5 | 27.74 | 30.01 | 30.1 |
| D6 | 26.13 | 28.92 | 27 | 30.83 | 29.97 | 28.84 | D6 | 24.97 | 26.86 | 26.23 | 24.62 | 27.08 | 27.96 |
| D7 | 20.05 | 20.12 | 20.07 | 23.27 | 21.65 | 22.87 | D7 | 26.99 | 27.4 | 29.46 | 25.16 | 25.65 | 27.85 |
| D8 | 26.63 | 28.17 | 27.59 | 31.99 | 30.51 | 30.06 | D8 | 28.68 | 29.5 | 29.76 | 28.77 | 30.82 | 30.83 |
| D9 | 26.88 | 28.5 | 26.86 | 30.98 | 29.65 | 29.58 | D9 | 33.14 | 35.17 | 38.53 | 31.58 | 34.64 | 35.44 |
| D10 | 29.53 | 36.54 | 35.31 | 37.46 | 37.62 | 35.53 | D10 | 32.21 | 22.13 | 21.81 | 24.13 | 24.23 | 24.28 |
| D11 | 27.46 | 29.1 | 29.73 | 31.93 | 31.75 | 32.62 | D11 | No Ct | No Ct | No Ct | 29.87 | No Ct | 33.76 |
| D12 | 28.12 | 29.66 | 28.66 | 31.86 | 31.22 | 31.98 | D12 | 16.84 | 17.69 | 18.04 | 16.53 | 18.02 | 19.73 |
| E1 | 27.56 | 27.82 | 27.45 | 29.86 | 27.2 | 28.02 | E1 | 30.1 | 32.12 | 32.03 | 28.49 | 31.3 | 31.96 |
| E2 | No Ct | 31.07 | No Ct | No Ct | 31.63 | 36.84 | E2 | 20.69 | 22.21 | 22.08 | 21.57 | 23.71 | 24.09 |
| E3 | 33.77 | No Ct | No Ct | No Ct | 33.44 | No Ct | E3 | 26.13 | 27.45 | 27.72 | 27.29 | 29.17 | 28.26 |
| E4 | 31.55 | No Ct | No Ct | No Ct | No Ct | No Ct | E4 | 27.39 | 28.53 | 27.08 | 27.01 | 29.21 | 28.72 |
| E5 | 29.7 | 31.23 | 30.58 | 31.36 | 32.41 | 30.75 | E5 | No Ct | No Ct | 33.11 | 28.44 | 32.69 | No Ct |
| E6 | 18.92 | 18.43 | 19.04 | 21.55 | 19.52 | 20.73 | E6 | 21.21 | 22.44 | 21.29 | 20.47 | 23.57 | 24.47 |
| E7 | 21.92 | 22.27 | 22.43 | 24.54 | 22.51 | 23.84 | E7 | 25.96 | 27.36 | 26.74 | 24.25 | 27.96 | 28.72 |
| E8 | 25.07 | 27.21 | 26.87 | 30.11 | 27.64 | 28.64 | E8 | 28.31 | 29.29 | 29.72 | 27.55 | 30.16 | 30.33 |
| E9 | 26.19 | 27.49 | 27.21 | 29.2 | 28.75 | 28.97 | E9 | 29.54 | 32.04 | 30.92 | 31.97 | 32.47 | 33.91 |
| E10 | 26.06 | 28.3 | 27.15 | 30.25 | 30.13 | 29.32 | E10 | 32.15 | 34.36 | No Ct | 28.29 | No Ct | No Ct |
| E11 | 22.16 | 24.33 | 23.27 | 26.93 | 25.21 | 25.6 | E11 | 26.33 | 29.5 | 32.12 | 25.46 | 30.25 | 30.78 |
| E12 | 28.04 | 28.45 | 34.43 | 28.63 | 29.41 | 32.61 | E12 | 31.64 | 34.33 | 35.52 | 28.1 | 29.85 | 32.63 |
| F1 | 26.8 | 27.88 | 28.31 | 28.4 | 29.61 | 29.09 | F1 | 31.03 | 32.25 | 31.67 | 30.7 | 33.26 | 39.82 |
| F2 | 27.93 | 29.3 | 29.79 | 30.66 | 29.18 | 29.29 | F2 | 33.76 | 34.7 | No Ct | 31.95 | No Ct | No Ct |
| F3 | 25.67 | 28.25 | 26.66 | 29.19 | 28.82 | 27.9 | F3 | 38.53 | 37.46 | 39.72 | No Ct | No Ct | No Ct |
| F4 | 25.83 | 27.53 | 27.32 | 31.39 | 29.4 | 29.54 | F4 | 18.56 | 18.68 | 19.85 | 20.48 | 20.91 | 22.21 |
| F5 | 27.99 | 27.78 | 30.28 | 31.73 | 27.5 | 28.44 | F5 | 37.49 | 34.27 | 38.22 | 31.9 | No Ct | 38.17 |
| F6 | No Ct | No Ct | No Ct | No Ct | No Ct | No Ct | F6 | 32.09 | 32.79 | 34.81 | 31.12 | 34.32 | 32.62 |
| F7 | 28.66 | 31.04 | 30.08 | 32.07 | 30.46 | 31.7 | F7 | 28.51 | 30.38 | 30.96 | 26.26 | 29.48 | 29.66 |
| F8 | 27.43 | 30.97 | 30.21 | 29.86 | 29.64 | 29.98 | F8 | 30.4 | 32.05 | 32.05 | 29.99 | 32.32 | 33.73 |
| F9 | 35.21 | 36.79 | 33.41 | No Ct | No Ct | 35.59 | F9 | 21.43 | 22.57 | 22.64 | 23.74 | 23.35 | 24.58 |
| F10 | 30 | 30.2 | 28.97 | 29.19 | 27.55 | 28.67 | F10 | 28.11 | 28.14 | 28.47 | 28.23 | 30.26 | 30.16 |
| F11 | 29.22 | 28.82 | 28.9 | 29.78 | 27.6 | 29.9 | F11 | 34.37 | 23.92 | 22.63 | 34.99 | 23.86 | 24.16 |
| F12 | 28.7 | 26.46 | 28.4 | 30.23 | 26.31 | 31.23 | F12 | 27.97 | 29.63 | 30.38 | 25.85 | 27.86 | No Ct |
| G1 | 25.4 | 26.99 | 26.26 | 27.33 | 26.38 | 28.2 | G1 | 34.51 | 34.75 | 34.92 | 31.59 | 37.97 | 37.56 |
| G2 | No Ct | No Ct | No Ct | No Ct | 34.28 | No Ct | G2 | 23.83 | 26.41 | 25.88 | 24.81 | 27.7 | 27.35 |
| G3 | 29 | 29.35 | 29.31 | 29.52 | 29.2 | 29.06 | G3 | 27.6 | 30.92 | 31.39 | 27.47 | 28.63 | 30.3 |
| G4 | 27.2 | 25.72 | 26.45 | 29.31 | 26.77 | 29.46 | G4 | 27.17 | 27.61 | 27.5 | 24.06 | 27.26 | 28.06 |
| G5 | 29.48 | 31.62 | 30.05 | 32.47 | 32.02 | 31.46 | G5 | 26.71 | 28.18 | 27.38 | 25.81 | 29.01 | 29.48 |
| G6 | 34.53 | 35.05 | 35.38 | 32.44 | 31.61 | No Ct | G6 | 27.11 | 31.16 | 29.68 | 26.21 | 29.4 | 28.68 |
| G7 | 25.8 | 26.69 | 25.92 | 28.52 | 27.59 | 28.28 | G7 | 26.77 | 28.97 | 30.97 | 27.04 | 27.65 | 37.04 |
| G8 | 26.69 | 28.12 | 27.5 | 30.55 | 29.36 | 28.77 | G8 | 24.14 | 25.37 | 26.81 | 25.39 | 26.44 | 26.89 |
| G9 | 27.37 | 29.08 | 27.15 | 31.32 | 29.6 | 29.8 | G9 | No Ct | No Ct | No Ct | No Ct | No Ct | No Ct |
| G10 | 27.26 | 30.58 | 28.53 | 34.95 | 31.15 | 29.91 | G10 | 22.44 | 23.48 | 23.47 | 23.37 | 23.79 | 24.63 |
| G11 | 32.37 | No Ct | 30.97 | 31.67 | No Ct | No Ct | G11 | 27.46 | 28.23 | 27.21 | 26.31 | 29.07 | 28.78 |
| G12 | 25.4 | 26.58 | 26.1 | 28.28 | 27.53 | 29.19 | G12 | 26.9 | 27.87 | 27.49 | 27.2 | 29.02 | 29.07 |
| H1 | 19 | 18.61 | 18.88 | 20.7 | 18.99 | 20.39 | H1 | 17.46 | 18.17 | 18.6 | 19.13 | 18.34 | 20.33 |
| H2 | 20.17 | 19.89 | 20.03 | 23.91 | 21.43 | 22.68 | H2 | 19.76 | 19.91 | 20.23 | 22.84 | 21.6 | 22.89 |
| H3 | 23.42 | 25.06 | 24.18 | 26.98 | 25.68 | 26.18 | H3 | 24.48 | 25.93 | 24.92 | 25.82 | 26.16 | 26.31 |
| H4 | 18.08 | 17.66 | 17.95 | 20.96 | 19.04 | 20.07 | H4 | 16.63 | 16.84 | 17.71 | 20.26 | 18.65 | 20.08 |
| H5 | 18.3 | 18.82 | 18.5 | 21.56 | 20.1 | 20.73 | H5 | 17.61 | 18.55 | 18.57 | 19.82 | 19.47 | 20.85 |
| H6 | No Ct | No Ct | No Ct | 32.45 | 30.09 | 36.02 | H6 | 31.3 | No Ct | No Ct | 29.29 | 30.49 | No Ct |
| H7 | 22.09 | 21.82 | 22.06 | 22.17 | 22.02 | 22.11 | H7 | 21.78 | 22.37 | 22.3 | 22.13 | 22.16 | 22.24 |
| H8 | 22.12 | 21.91 | 21.95 | 21.98 | 22.21 | 22.48 | H8 | 22.19 | 22.26 | 22.34 | 21.91 | 22.18 | 22.29 |
| H9 | 22.22 | 22.05 | 22.06 | 22.37 | 22.13 | 22.31 | H9 | 21.99 | 22.56 | 22.27 | 22.31 | 22.4 | 22.56 |
| H10 | 17.85 | 18.2 | 17.83 | 18.65 | 18.48 | 18.7 | H10 | 16.9 | 16.87 | 16.97 | 20.24 | 16.47 | 16.96 |
| H11 | 17.94 | 18.07 | 17.99 | 18.64 | 18.69 | 18.44 | H11 | 17.23 | 16.72 | 16.99 | 20.48 | 16.52 | 17.18 |
| H12 | 18.02 | 18.52 | 17.92 | 18.84 | 18.52 | 18.56 | H12 | 17.32 | 18.5 | 17.2 | 20.21 | 16.42 | 17.32 |

Array data represent raw CT values for each of the three biological replicate samples. The codes for identification of genes in each row of the PCR array are provided on the SA Biosciences website.
