# Supplementary material for: Pathway-Focused PCR Array Profiling of Enriched Populations of Laser Capture Microdissected Hippocampal Cells after Traumatic Brain Injury
Source: PLoS One. 2015 May 27;10(5):e0127287. doi: 10.1371/journal.pone.0127287 (PMC4446038; doi:10.1371/journal.pone.0127287)
Supplement: S4 Table — (DOCX) [file pone.0127287.s006.docx]

| **Table S4. Comparison of genes in Agilent microarrays and pathway-specific PCR arrays.** | | | | |
| --- | --- | --- | --- | --- |
|  | PCR Fold Change | Array Ratio | PCR P‑value | Full Name |
| Neurotrophin set | | | | |
| Bdnf | 0.5192 | 6.349 | 0.39019 | Brain-derived neurotrophic factor |
| Hprt1 | 0.6349 | 5.324 | 0.101294 | Hypoxanthine phosphoribosyltransferase 1 |
| Il1b | 3.5173 | -5.917 | 0.025003 | Interleukin 1 beta |
| Stat3 | 2.1702 | <5fold | 0.548485 | Signal transducer and activator of transcription 3 |
| Apoptosis set | | | | |
| Casp3 | 2.0801 | -10.194 | 0.002937 | Caspase 3 |
| Hprt1 | 0.7649 | 5.324 | 0.38904 | Hypoxanthine phosphoribosyltransferase 1 |
| Mcl1 | 1.7818 | -5.236 | 0.054103 | Myeloid cell leukemia sequence 1 |
| Comparison of differential gene expression in the microarrays vs PCR arrays showed similar trends (expression higher or lower in dying vs surviving cells) in 9 common genes found differentially expressed between dying/injured and surviving/uninjured neurons, i.e. in the PCR arrays, the gene expression ratios reflect fold changes (increase or decrease) in dying vs surviving neurons, values <1 mean that expression was lower (blue) in dying neurons and values>1 mean that expression was higher (pink) in dying neurons. In the microarrays, the colors have a different meaning, blue represents 5-fold or greater expression levels in dying vs surviving neurons and pink represents 5-fold or greater expression in surviving vs dying neurons. | | | | |
