# Supplementary material for: Pathway-Focused PCR Array Profiling of Enriched Populations of Laser Capture Microdissected Hippocampal Cells after Traumatic Brain Injury
Source: PLoS One. 2015 May 27;10(5):e0127287. doi: 10.1371/journal.pone.0127287 (PMC4446038; doi:10.1371/journal.pone.0127287)
Supplement: S1 References — (DOC) [file pone.0127287.s007.doc]

Supplemental Reference List

Alder J, Thakker-Varia S, Bangasser DA, Kuroiwa M, Plummer MR, Shors TJ, Black IB. Brain-derived neurotrophic factor-induced gene expression reveals novel actions of VGF in hippocampal synaptic plasticity. J Neurosci, 2003; 23: 10800-8.

Babcock JT, Nguyen HB, He Y, Hendricks JW, Wek RC, Quilliam LA. Mammalian target of rapamycin complex 1 (mTORC1) enhances bortezomib-induced death in tuberous sclerosis complex (TSC)-null cells by a c-MYC-dependent induction of the unfolded protein response. J Biol. Chem., 2013; 288: 15687-98.

Baloh RH, Tansey MG, Lampe PA, Fahrner TJ, Enomoto H, Simburger KS, Leitner ML, Araki T, Johnson EM, Jr., Milbrandt J. Artemin, a novel member of the GDNF ligand family, supports peripheral and central neurons and signals through the GFRalpha3-RET receptor complex. Neuron, 1998; 21: 1291-302.

Barrera-Vilarmau S, Obregon P, de AE. Intrinsic order and disorder in the bcl-2 member harakiri: insights into its proapoptotic activity. PLoS. One., 2011; 6: e21413.

Besirli CG, Chinskey ND, Zheng QD, Zacks DN. Autophagy activation in the injured photoreceptor inhibits fas-mediated apoptosis. Invest Ophthalmol. Vis. Sci, 2011; 52: 4193-9.

Blaise S, Kneib M, Rousseau A, Gambino F, Chenard MP, Messadeq N, Muckenstrum M, Alpy F, Tomasetto C, Humeau Y, Rio MC. In vivo evidence that TRAF4 is required for central nervous system myelin homeostasis. PLoS. One., 2012; 7: e30917.

Boku S, Nakagawa S, Takamura N, Kato A, Takebayashi M, Hisaoka-Nakashima K, Omiya Y, Inoue T, Kusumi I. GDNF facilitates differentiation of the adult dentate gyrus-derived neural precursor cells into astrocytes via STAT3. Biochem. Biophys. Res. Commun., 2013; 434: 779-84.

Brooks C, Wei Q, Feng L, Dong G, Tao Y, Mei L, Xie ZJ, Dong Z. Bak regulates mitochondrial morphology and pathology during apoptosis by interacting with mitofusins. Proc Natl. Acad. Sci U. S. A, 2007; 104: 11649-54.

Buga AM, Scholz CJ, Kumar S, Herndon JG, Alexandru D, Cojocaru GR, Dandekar T, Popa-Wagner A. Identification of new therapeutic targets by genome-wide analysis of gene expression in the ipsilateral cortex of aged rats after stroke. PLoS. One., 2012; 7: e50985.

Butterick TA, Nixon JP, Billington CJ, Kotz CM. Orexin A decreases lipid peroxidation and apoptosis in a novel hypothalamic cell model. Neurosci Lett., 2012; 524: 30-4.

Buyukuysal RL. Protein S100B release from rat brain slices during and after ischemia: comparison with lactate dehydrogenase leakage. Neurochem. Int., 2005; 47: 580-8.

Calingasan NY, Erdely HA, Altar CA. Identification of CD40 ligand in Alzheimer's disease and in animal models of Alzheimer's disease and brain injury. Neurobiol. Aging, 2002; 23: 31-9.

Camici M, Micheli V, Ipata PL, Tozzi MG. Pediatric neurological syndromes and inborn errors of purine metabolism. Neurochem. Int., 2010; 56: 367-78.

Cartagena CM, Schmid KE, Phillips KL, Tortella FC, Dave JR. Changes in apoptotic mechanisms following penetrating ballistic-like brain injury. J Mol. Neurosci, 2013; 49: 301-11.

Chakraborty S, Li L, Tang H, Xie Y, Puliyappadamba VT, Raisanen J, Burma S, Boothman DA, Cochran B, Wu J, Habib AA. Cytoplasmic TRADD confers a worse prognosis in glioblastoma. Neoplasia., 2013; 15: 888-97.

Chang MX, Chen WQ, Nie P. Structure and expression pattern of teleost caspase recruitment domain (CARD) containing proteins that are potentially involved in NF-kappaB signalling. Dev. Comp Immunol., 2010; 34: 1-13.

Chen L, McKenna JT, Bolortuya Y, Brown RE, McCarley RW. Knockdown of orexin type 2 receptor in the lateral pontomesencephalic tegmentum of rats increases REM sleep. Eur. J Neurosci, 2013; 37: 957-63.

Chen Z, Norris JY, Finck BN. Peroxisome proliferator-activated receptor-gamma coactivator-1alpha (PGC-1alpha) stimulates VLDL assembly through activation of cell death-inducing DFFA-like effector B (CideB). J Biol. Chem., 2010; 285: 25996-6004.

Cheng MY, Lee AG, Culbertson C, Sun G, Talati RK, Manley NC, Li X, Zhao H, Lyons DM, Zhou QY, Steinberg GK, Sapolsky RM. Prokineticin 2 is an endangering mediator of cerebral ischemic injury. Proc Natl. Acad. Sci U. S. A, 2012; 109: 5475-80.

Cho B, Choi SY, Park OH, Sun W, Geum D. Differential expression of BNIP family members of BH3-only proteins during the development and after axotomy in the rat. Mol. Cells, 2012; 33: 605-10.

Curtis KM, Gomez LA, Rios C, Garbayo E, Raval AP, Perez-Pinzon MA, Schiller PC. EF1alpha and RPL13a represent normalization genes suitable for RT-qPCR analysis of bone marrow derived mesenchymal stem cells. BMC. Mol. Biol., 2010; 11: 61.

Dash PK, Zhao J, Hergenroeder G, Moore AN. Biomarkers for the diagnosis, prognosis, and evaluation of treatment efficacy for traumatic brain injury. Neurotherapeutics., 2010; 7: 100-14.

Deverman BE, Patterson PH. Exogenous leukemia inhibitory factor stimulates oligodendrocyte progenitor cell proliferation and enhances hippocampal remyelination. J Neurosci, 2012; 32: 2100-9.

Di P, V, Amorini AM, Tavazzi B, Hovda DA, Signoretti S, Giza CC, Lazzarino G, Vagnozzi R, Lazzarino G, Belli A. Potentially neuroprotective gene modulation in an in vitro model of mild traumatic brain injury. Mol. Cell Biochem., 2013; 375: 185-98.

Diaz-Horta O, Kamagate A, Herchuelz A, Van EF. Na/Ca exchanger overexpression induces endoplasmic reticulum-related apoptosis and caspase-12 activation in insulin-releasing BRIN-BD11 cells. Diabetes, 2002; 51: 1815-24.

Dmitrieva VG, Dergunova LV, Povarova OV, Skvortsova VI, Limborskaya SA, Myasoedov NF. The effect of semax and the C-terminal peptide PGP on expression of growth factor genes and receptors in rats under conditions of experimental cerebral ischemia. Dokl. Biochem. Biophys., 2008; 422: 261-4.

Dufner A, Pownall S, Mak TW. Caspase recruitment domain protein 6 is a microtubule-interacting protein that positively modulates NF-kappaB activation. Proc Natl. Acad. Sci U. S. A, 2006; 103: 988-93.

Dunn HA, Walther C, Godin CM, Hall RA, Ferguson SS. Role of SAP97 protein in the regulation of corticotropin-releasing factor receptor 1 endocytosis and extracellular signal-regulated kinase 1/2 signaling. J Biol. Chem., 2013; 288: 15023-34.

Dutia R, Kim AJ, Mosharov E, Savontaus E, Chua SC, Jr., Wardlaw SL. Regulation of prolactin in mice with altered hypothalamic melanocortin activity. Peptides, 2012; 37: 6-12.

Echeverry R, Wu F, Haile WB, Wu J, Yepes M. The cytokine tumor necrosis factor-like weak inducer of apoptosis and its receptor fibroblast growth factor-inducible 14 have a neuroprotective effect in the central nervous system. J Neuroinflammation, 2012; 9: 45.

Espinosa-Garcia C, Vigueras-Villasenor RM, Rojas-Castaneda JC, guilar-Hernandez A, Monfil T, Cervantes M, Morali G. Post-ischemic administration of progesterone reduces caspase-3 activation and DNA fragmentation in the hippocampus following global cerebral ischemia. Neurosci Lett., 2013; 550: 98-103.

Ferri GL, Noli B, Brancia C, D'Amato F, Cocco C. VGF: an inducible gene product, precursor of a diverse array of neuro-endocrine peptides and tissue-specific disease biomarkers. J Chem. Neuroanat., 2011; 42: 249-61.

Forrest SL, Keast JR. Expression of receptors for glial cell line-derived neurotrophic factor family ligands in sacral spinal cord reveals separate targets of pelvic afferent fibers. J Comp Neurol., 2008; 506: 989-1002.

Fronczek R, Baumann CR, Lammers GJ, Bassetti CL, Overeem S. Hypocretin/orexin disturbances in neurological disorders. Sleep Med Rev., 2009; 13: 9-22.

Gaughran F, Payne J, Sedgwick PM, Cotter D, Berry M. Hippocampal FGF-2 and FGFR1 mRNA expression in major depression, schizophrenia and bipolar disorder. Brain Res. Bull., 2006; 70: 221-7.

Gibson L, Holmgreen SP, Huang DC, Bernard O, Copeland NG, Jenkins NA, Sutherland GR, Baker E, Adams JM, Cory S. bcl-w, a novel member of the bcl-2 family, promotes cell survival. Oncogene, 1996; 13: 665-75.

Goc A, Kochuparambil ST, Al-Husein B, Al-Azayzih A, Mohammad S, Somanath PR. Simultaneous modulation of the intrinsic and extrinsic pathways by simvastatin in mediating prostate cancer cell apoptosis. BMC. Cancer, 2012; 12: 409.

Gonzalez P, Burgaya F, Acarin L, Peluffo H, Castellano B, Gonzalez B. Interleukin-10 and interleukin-10 receptor-I are upregulated in glial cells after an excitotoxic injury to the postnatal rat brain. J Neuropathol. Exp Neurol., 2009; 68: 391-403.

Graham RK, Deng Y, Carroll J, Vaid K, Cowan C, Pouladi MA, Metzler M, Bissada N, Wang L, Faull RL, Gray M, Yang XW, Raymond LA, Hayden MR. Cleavage at the 586 amino acid caspase-6 site in mutant huntingtin influences caspase-6 activation in vivo. J Neurosci, 2010; 30: 15019-29.

Grammatopoulos DK, Chrousos GP. Functional characteristics of CRH receptors and potential clinical applications of CRH-receptor antagonists. Trends Endocrinol. Metab, 2002; 13: 436-44.

Gridley CL, Rangarajan S, Firbank S, Dalal S, Sweasy JB, Jaeger J. Structural changes in the hydrophobic hinge region adversely affect the activity and fidelity of the I260Q mutator DNA polymerase beta. Biochemistry, 2013; 52: 4422-32.

Guerra-Crespo M, Gleason D, Sistos A, Toosky T, Solaroglu I, Zhang JH, Bryant PJ, Fallon JH. Transforming growth factor-alpha induces neurogenesis and behavioral improvement in a chronic stroke model. Neuroscience, 2009; 160: 470-83.

Guo WP, Wang J, Li RX, Peng YW. Neuroprotective effects of neuregulin-1 in rat models of focal cerebral ischemia. Brain Res., 2006; 1087: 180-5.

Hakem R, Hakem A, Duncan GS, Henderson JT, Woo M, Soengas MS, Elia A, de la Pompa JL, Kagi D, Khoo W, Potter J, Yoshida R, Kaufman SA, Lowe SW, Penninger JM, Mak TW. Differential requirement for caspase 9 in apoptotic pathways in vivo. Cell, 1998; 94: 339-52.

Hasan SM, Sheen AD, Power AM, Langevin LM, Xiong J, Furlong M, Day K, Schuurmans C, Opferman JT, Vanderluit JL. Mcl1 regulates the terminal mitosis of neural precursor cells in the mammalian brain through p27Kip1. Development, 2013; 140: 3118-27.

He J, Huang C, Jiang J, Lv L. Propofol exerts hippocampal neuron protective effects via up-regulation of metallothionein-3. Neurol. Sci, 2013; 34: 165-71.

He JQ, Zarnegar B, Oganesyan G, Saha SK, Yamazaki S, Doyle SE, Dempsey PW, Cheng G. Rescue of TRAF3-null mice by p100 NF-kappa B deficiency. J Exp Med, 2006; 203: 2413-8.

Hernandez-Acosta NC, Cabrera-Socorro A, Morlans MP, Delgado FJ, Suarez-Sola ML, Sottocornola R, Lu X, Gonzalez-Gomez M, Meyer G. Dynamic expression of the p53 family members p63 and p73 in the mouse and human telencephalon during development and in adulthood. Brain Res., 2011; 1372: 29-40.

Hitomi J, Katayama T, Eguchi Y, Kudo T, Taniguchi M, Koyama Y, Manabe T, Yamagishi S, Bando Y, Imaizumi K, Tsujimoto Y, Tohyama M. Involvement of caspase-4 in endoplasmic reticulum stress-induced apoptosis and Abeta-induced cell death. J Cell Biol., 2004; 165: 347-56.

Hoh NZ, Wagner AK, Alexander SA, Clark RB, Beers SR, Okonkwo DO, Ren D, Conley YP. BCL2 genotypes: functional and neurobehavioral outcomes after severe traumatic brain injury. J Neurotrauma, 2010; 27: 1413-27.

Holm L, Hilke S, Adori C, Theodorsson E, Hokfelt T, Theodorsson A. Changes in galanin and GalR1 gene expression in discrete brain regions after transient occlusion of the middle cerebral artery in female rats. Neuropeptides, 2012; 46: 19-27.

Hsieh YS, Kuo MH, Chen PN, Kuo DY. The identification of neuropeptide Y receptor subtype involved in phenylpropanolamine-induced increase in oxidative stress and appetite suppression. Neuromolecular. Med, 2013; 15: 159-68.

Hsu SY, Kaipia A, Zhu L, Hsueh AJ. Interference of BAD (Bcl-xL/Bcl-2-associated death promoter)-induced apoptosis in mammalian cells by 14-3-3 isoforms and P11. Mol. Endocrinol., 1997; 11: 1858-67.

Hsu WL, Chiu TH, Tai DJ, Ma YL, Lee EH. A novel defense mechanism that is activated on amyloid-beta insult to mediate cell survival: role of SGK1-STAT1/STAT2 signaling. Cell Death. Differ., 2009; 16: 1515-29.

Hu S, Ying Z, Gomez-Pinilla F, Frautschy SA. Exercise can increase small heat shock proteins (sHSP) and pre- and post-synaptic proteins in the hippocampus. Brain Res., 2009; 1249: 191-201.

Huang C, Xia PY, Zhou H. Sustained expression of TDP-43 and FUS in motor neurons in rodent's lifetime. Int. J Biol. Sci, 2010; 6: 396-406.

Huang JY, Chuang JI. Fibroblast growth factor 9 upregulates heme oxygenase-1 and gamma-glutamylcysteine synthetase expression to protect neurons from 1-methyl-4-phenylpyridinium toxicity. Free Radic. Biol. Med, 2010; 49: 1099-108.

Hung CC, Jen TJ, Kao PJ, Lin MS, Liou HH. Association of polymorphisms in NR1I2 and ABCB1 genes with epilepsy treatment responses. Pharmacogenomics., 2007; 8: 1151-8.

Hung TM, Hu RH, Ho CM, Chiu YL, Lee JL, Jeng YM, Shih DT, Lee PH. Downregulation of alpha-fetoprotein expression by LHX4: a critical role in hepatocarcinogenesis. Carcinogenesis, 2011; 32: 1815-23.

Iglesias-Guimarais V, Gil-Guinon E, Sanchez-Osuna M, Casanelles E, Garcia-Belinchon M, Comella JX, Yuste VJ. Chromatin collapse during caspase-dependent apoptotic cell death requires DNA fragmentation factor, 40-kDa subunit-/caspase-activated deoxyribonuclease-mediated 3'-OH single-strand DNA breaks. J Biol. Chem., 2013; 288: 9200-15.

Inui S, Noguchi F, Nishiyama A, Itami S. Multipotential functions of Hic-5 in growth, differentiation, migration and adhesion of human keratinocytes. J Dermatol. Sci, 2012; 68: 197-9.

Ito M, Nagasawa M, Omae N, Ide T, Akasaka Y, Murakami K. Differential regulation of CIDEA and CIDEC expression by insulin via Akt1/2- and JNK2-dependent pathways in human adipocytes. J Lipid Res., 2011; 52: 1450-60.

Iwata A, Morgan-Stevenson V, Schwartz B, Liu L, Tupper J, Zhu X, Harlan J, Winn R. Extracellular BCL2 proteins are danger-associated molecular patterns that reduce tissue damage in murine models of ischemia-reperfusion injury. PLoS. One., 2010; 5: e9103.

Jabado O, Wang Q, Rideout HJ, Yeasmin M, Guo KX, Vekrellis K, Papantonis S, Angelastro JM, Troy CM, Stefanis L. RAIDD aggregation facilitates apoptotic death of PC12 cells and sympathetic neurons. Cell Death. Differ., 2004; 11: 618-30.

Jarvinen K, Hotti A, Santos L, Nummela P, Holtta E. Caspase-8, c-FLIP, and caspase-9 in c-Myc-induced apoptosis of fibroblasts. Exp Cell Res., 2011; 317: 2602-15.

Johnson CE, Huang YY, Parrish AB, Smith MI, Vaughn AE, Zhang Q, Wright KM, Van DT, Wechsler-Reya RJ, Kornbluth S, Deshmukh M. Differential Apaf-1 levels allow cytochrome c to induce apoptosis in brain tumors but not in normal neural tissues. Proc Natl. Acad. Sci U. S. A, 2007; 104: 20820-5.

Kamm K, Vanderkolk W, Lawrence C, Jonker M, Davis AT. The effect of traumatic brain injury upon the concentration and expression of interleukin-1beta and interleukin-10 in the rat. J Trauma, 2006; 60: 152-7.

Kanter-Schlifke I, Fjord-Larsen L, Kusk P, Angehagen M, Wahlberg L, Kokaia M. GDNF released from encapsulated cells suppresses seizure activity in the epileptic hippocampus. Exp Neurol., 2009; 216: 413-9.

Kawakami T, Wakabayashi Y, Aimi Y, Isono T, Okada Y. Developmental expression of glial cell-line derived neurotrophic factor, neurturin, and their receptor mRNA in the rat urinary bladder. Neurourol. Urodyn., 2003; 22: 83-8.

Ke F, Bouillet P, Kaufmann T, Strasser A, Kerr J, Voss AK. Consequences of the combined loss of BOK and BAK or BOK and BAX. Cell Death. Dis., 2013; 4: e650.

Keane RW, Kraydieh S, Lotocki G, Alonso OF, Aldana P, Dietrich WD. Apoptotic and antiapoptotic mechanisms after traumatic brain injury. J. Cereb. Blood Flow Metab, 2001; 21: 1189-98.

Kholodilov N, Kim SR, Yarygina O, Kareva T, Cho JW, Baohan A, Burke RE. Glial cell line-derived neurotrophic factor receptor-alpha1 expressed in striatum in trans regulates development and injury response of dopamine neurons of the substantia nigra. J Neurochem., 2011; 116: 486-98.

Kim HW, Haider HK, Jiang S, Ashraf M. Ischemic preconditioning augments survival of stem cells via miR-210 expression by targeting caspase-8-associated protein 2. J Biol. Chem., 2009; 284: 33161-8.

Kim SJ, Zhang Z, Hitomi E, Lee YC, Mukherjee AB. Endoplasmic reticulum stress-induced caspase-4 activation mediates apoptosis and neurodegeneration in INCL. Hum. Mol. Genet., 2006; 15: 1826-34.

Krajewska M, You Z, Rong J, Kress C, Huang X, Yang J, Kyoda T, Leyva R, Banares S, Hu Y, Sze CH, Whalen MJ, Salmena L, Hakem R, Head BP, Reed JC, Krajewski S. Neuronal deletion of caspase 8 protects against brain injury in mouse models of controlled cortical impact and kainic acid-induced excitotoxicity. PLoS. One., 2011; 6: e24341.

Lai AY, Swayze RD, El-Husseini A, Song C. Interleukin-1 beta modulates AMPA receptor expression and phosphorylation in hippocampal neurons. J Neuroimmunol., 2006; 175: 97-106.

Lam SP, Luk JM, Man K, Ng KT, Cheung CK, Rose-John S, Lo CM. Activation of interleukin-6-induced glycoprotein 130/signal transducer and activator of transcription 3 pathway in mesenchymal stem cells enhances hepatic differentiation, proliferation, and liver regeneration. Liver Transpl., 2010; 16: 1195-206.

Larner SF, McKinsey DM, Hayes RL, KK WW. Caspase 7: increased expression and activation after traumatic brain injury in rats. J Neurochem., 2005; 94: 97-108.

Leadbeater WE, Gonzalez AM, Logaras N, Berry M, Turnbull JE, Logan A. Intracellular trafficking in neurones and glia of fibroblast growth factor-2, fibroblast growth factor receptor 1 and heparan sulphate proteoglycans in the injured adult rat cerebral cortex. J Neurochem., 2006; 96: 1189-200.

Leak RK, Zhang L, Luo Y, Li P, Zhao H, Liu X, Ling F, Jia J, Chen J, Ji X. Peroxiredoxin 2 battles poly(ADP-ribose) polymerase 1- and p53-dependent prodeath pathways after ischemic injury. Stroke, 2013; 44: 1124-34.

Lee KH, Yu DH, Lee YS. Gene expression profiling of rat cerebral cortex development using cDNA microarrays. Neurochem. Res., 2009; 34: 1030-8.

Lee N, Batt MK, Cronier BA, Jackson MC, Bruno Garza JL, Trinh DS, Mason CO, Spearry RP, Bhattacharya S, Robitz R, Nakafuku M, MacLennan AJ. Ciliary neurotrophic factor receptor regulation of adult forebrain neurogenesis. J Neurosci, 2013; 33: 1241-58.

Li Z, Sun C, Zhang T, Mo J, Shi Q, Zhang X, Yuan M, Chen L, Mao X, Yu R, Zhou X. Geranylgeranyltransferase I mediates BDNF-induced synaptogenesis. J Neurochem., 2013; 125: 698-712.

Lomonosova E, Chinnadurai G. BH3-only proteins in apoptosis and beyond: an overview. Oncogene, 2008; 27 Suppl 1: S2-19.

Longart M, Liu Y, Karavanova I, Buonanno A. Neuregulin-2 is developmentally regulated and targeted to dendrites of central neurons. J Comp Neurol., 2004; 472: 156-72.

Malik IA, Triebel J, Posselt J, Khan S, Ramadori P, Raddatz D, Ramadori G. Melanocortin receptors in rat liver cells: change of gene expression and intracellular localization during acute-phase response. Histochem. Cell Biol., 2012; 137: 279-91.

Mao W, Yi X, Qin J, Tian M, Jin G. CXCL12 inhibits cortical neuron apoptosis by increasing the ratio of Bcl-2/Bax after traumatic brain injury. Int. J Neurosci, 2013; ahead of print.

Martins-de-Souza D, Guest PC, Mann DM, Roeber S, Rahmoune H, Bauder C, Kretzschmar H, Volk B, Baborie A, Bahn S. Proteomic analysis identifies dysfunction in cellular transport, energy, and protein metabolism in different brain regions of atypical frontotemporal lobar degeneration. J Proteome. Res., 2012; 11: 2533-43.

Mastrandrea LD, Sessanna SM, Del TA, Laychock SG. ATP-independent glucose stimulation of sphingosine kinase in rat pancreatic islets. J Lipid Res., 2010; 51: 2171-80.

Masumoto J, Zhou W, Chen FF, Su F, Kuwada JY, Hidaka E, Katsuyama T, Sagara J, Taniguchi S, Ngo-Hazelett P, Postlethwait JH, Nunez G, Inohara N. Caspy, a zebrafish caspase, activated by ASC oligomerization is required for pharyngeal arch development. J Biol. Chem., 2003; 278: 4268-76.

Matsumori Y, Northington FJ, Hong SM, Kayama T, Sheldon RA, Vexler ZS, Ferriero DM, Weinstein PR, Liu J. Reduction of caspase-8 and -9 cleavage is associated with increased c-FLIP and increased binding of Apaf-1 and Hsp70 after neonatal hypoxic/ischemic injury in mice overexpressing Hsp70. Stroke, 2006; 37: 507-12.

Mayer BA, Rehberg M, Erhardt A, Wolf A, Reichel CA, Kracht M, Krombach F, Tiegs G, Zahler S, Vollmar AM, Furst R. Inhibitor of apoptosis proteins as novel targets in inflammatory processes. Arterioscler. Thromb. Vasc. Biol., 2011; 31: 2240-50.

Mc GC, Volckaert T, Wolke U, Sze M, de RR, Waisman A, Prinz M, Beyaert R, Pasparakis M, van LG. Oligodendrocyte-specific FADD deletion protects mice from autoimmune-mediated demyelination. J Immunol., 2010; 185: 7646-53.

Medrano S, Burns-Cusato M, Atienza MB, Rahimi D, Scrable H. Regenerative capacity of neural precursors in the adult mammalian brain is under the control of p53. Neurobiol. Aging, 2009; 30: 483-97.

Mehmeti I, Gurgul-Convey E, Lenzen S, Lortz S. Induction of the intrinsic apoptosis pathway in insulin-secreting cells is dependent on oxidative damage of mitochondria but independent of caspase-12 activation. Biochim. Biophys. Acta, 2011; 1813: 1827-35.

Mihara M, Hashizume M, Yoshida H, Suzuki M, Shiina M. IL-6/IL-6 receptor system and its role in physiological and pathological conditions. Clin. Sci (Lond), 2012; 122: 143-59.

Milbrandt J, de Sauvage FJ, Fahrner TJ, Baloh RH, Leitner ML, Tansey MG, Lampe PA, Heuckeroth RO, Kotzbauer PT, Simburger KS, Golden JP, Davies JA, Vejsada R, Kato AC, Hynes M, Sherman D, Nishimura M, Wang LC, Vandlen R, Moffat B, Klein RD, Poulsen K, Gray C, Garces A, Johnson EM, Jr., . Persephin, a novel neurotrophic factor related to GDNF and neurturin. Neuron, 1998; 20: 245-53.

Minnich JE, Mann SL, Stock M, Stolzenbach KA, Mortell BM, Soderstrom KE, Bohn MC, Kozlowski DA. Glial cell line-derived neurotrophic factor (GDNF) gene delivery protects cortical neurons from dying following a traumatic brain injury. Restor. Neurol. Neurosci, 2010; 28: 293-309.

Mishra OP, ivoria-Papadopoulos M. Mechanism of tyrosine phosphorylation of procaspase-9 and Apaf-1 in cytosolic fractions of the cerebral cortex of newborn piglets during hypoxia. Neurosci Lett., 2010; 480: 35-9.

Mitchell GC, Wang Q, Ramamoorthy P, Whim MD. A common single nucleotide polymorphism alters the synthesis and secretion of neuropeptide Y. J Neurosci, 2008; 28: 14428-34.

Montazeri F, Esmaeili A, Miroliaei M, Moshtaghian SJ. Messenger RNA expression patterns of p75 neurotrophin receptor and tropomyosin-receptor-kinase A following spinal cord injury. J Spinal Cord. Med, 2013; 36: 231-6.

Mori M, Burgess DL, Gefrides LA, Foreman PJ, Opferman JT, Korsmeyer SJ, Cavalheiro EA, Naffah-Mazzacoratti MG, Noebels JL. Expression of apoptosis inhibitor protein Mcl1 linked to neuroprotection in CNS neurons. Cell Death. Differ., 2004; 11: 1223-33.

Nair S, Hagberg H, Krishnamurthy R, Thornton C, Mallard C. Death associated protein kinases: molecular structure and brain injury. Int. J Mol. Sci, 2013; 14: 13858-72.

Nakadate K, Imamura K, Watanabe Y. c-Fos activity mapping reveals differential effects of noradrenaline and serotonin depletion on the regulation of ocular dominance plasticity in rats. Neuroscience, 2013; 235: 1-9.

Nilufer YG, Dodurga Y, Kurtulus A, Boz B, Acar K. Caspase 1, caspase 3, TNF-alpha, p53, and Hif1-alpha gene expression status of the brain tissues and hippocampal neuron loss in short-term dichlorvos exposed rats. Mol. Biol. Rep., 2012; 39: 10355-60.

Nogueira-Silva C, Piairo P, Carvalho-Dias E, Peixoto FO, Moura RS, Correia-Pinto J. Leukemia inhibitory factor in rat fetal lung development: expression and functional studies. PLoS. One., 2012; 7: e30517.

Odemis V, Moepps B, Gierschik P, Engele J. Interleukin-6 and cAMP induce stromal cell-derived factor-1 chemotaxis in astroglia by up-regulating CXCR4 cell surface expression. Implications for brain inflammation. J Biol. Chem., 2002; 277: 39801-8.

Omae N, Ito M, Hase S, Nagasawa M, Ishiyama J, Ide T, Murakami K. Suppression of FoxO1/cell death-inducing DNA fragmentation factor alpha-like effector A (Cidea) axis protects mouse beta-cells against palmitic acid-induced apoptosis. Mol. Cell Endocrinol., 2012; 348: 297-304.

Onder L, Danuser R, Scandella E, Firner S, Chai Q, Hehlgans T, Stein JV, Ludewig B. Endothelial cell-specific lymphotoxin-beta receptor signaling is critical for lymph node and high endothelial venule formation. J Exp Med, 2013; 210: 465-73.

Oshitari T, Yoshida-Hata N, Yamamoto S. Effect of neurotrophin-4 on endoplasmic reticulum stress-related neuronal apoptosis in diabetic and high glucose exposed rat retinas. Neurosci Lett., 2011; 501: 102-6.

Ozisik K, Ozisik P, Yildirim E, Misirlioglu M, Tuncer S. Expression of antiapoptotic survivin and aven genes in rat heart tissue after traumatic brain injury. Transplant. Proc, 2006; 38: 2784-7.

Paintlia MK, Paintlia AS, Singh AK, Singh I. S-nitrosoglutathione induces ciliary neurotrophic factor expression in astrocytes, which has implications to protect the central nervous system under pathological conditions. J Biol. Chem., 2013; 288: 3831-43.

Park KI, Himes BT, Stieg PE, Tessler A, Fischer I, Snyder EY. Neural stem cells may be uniquely suited for combined gene therapy and cell replacement: Evidence from engraftment of Neurotrophin-3-expressing stem cells in hypoxic-ischemic brain injury. Exp Neurol., 2006; 199: 179-90.

Pennarun B, Meijer A, de Vries EG, Kleibeuker JH, Kruyt F, de JS. Playing the DISC: turning on TRAIL death receptor-mediated apoptosis in cancer. Biochim. Biophys. Acta, 2010; 1805: 123-40.

Petkova-Kirova P, Giovannini MG, Kalfin R, Rakovska A. Modulation of acetylcholine release by cholecystokinin in striatum: receptor specificity; role of dopaminergic neuronal activity. Brain Res. Bull., 2012; 89: 177-84.

Philips MF, Mattiasson G, Wieloch T, Bjorklund A, Johansson BB, Tomasevic G, Martinez-Serrano A, Lenzlinger PM, Sinson G, Grady MS, McIntosh TK. Neuroprotective and behavioral efficacy of nerve growth factor-transfected hippocampal progenitor cell transplants after experimental traumatic brain injury. J. Neurosurg., 2001; 94: 765-74.

Pizzi M, Sarnico I, Lanzillotta A, Battistin L, Spano P. Post-ischemic brain damage: NF-kappaB dimer heterogeneity as a molecular determinant of neuron vulnerability. FEBS J, 2009; 276: 27-35.

Planchamp V, Bermel C, Tonges L, Ostendorf T, Kugler S, Reed JC, Kermer P, Bahr M, Lingor P. BAG1 promotes axonal outgrowth and regeneration in vivo via Raf-1 and reduction of ROCK activity. Brain, 2008; 131: 2606-19.

Poon WW, Carlos AJ, Aguilar BL, Berchtold NC, Kawano CK, Zograbyan V, Yaopruke T, Shelanski M, Cotman CW. beta-Amyloid (Abeta) oligomers impair brain-derived neurotrophic factor retrograde trafficking by down-regulating ubiquitin C-terminal hydrolase, UCH-L1. J Biol. Chem., 2013; 288: 16937-48.

Presti-Torres J, de Lima MN, Scalco FS, Caldana F, Garcia VA, Guimaraes MR, Schwartsmann G, Roesler R, Schroder N. Impairments of social behavior and memory after neonatal gastrin-releasing peptide receptor blockade in rats: Implications for an animal model of neurodevelopmental disorders. Neuropharmacology, 2007; 52: 724-32.

Qu R, Li Y, Gao Q, Shen L, Zhang J, Liu Z, Chen X, Chopp M. Neurotrophic and growth factor gene expression profiling of mouse bone marrow stromal cells induced by ischemic brain extracts. Neuropathology., 2007; 27: 355-63.

Quan Y, Jiang J, Dingledine R. EP2 receptor signaling pathways regulate classical activation of microglia. J Biol. Chem., 2013; 288: 9293-302.

Robinson K, Vona-Davis L, Riggs D, Jackson B, McFadden D. Peptide YY attenuates STAT1 and STAT3 activation induced by TNF-alpha in acinar cell line AR42J. J Am. Coll. Surg., 2006; 202: 788-96.

Rousselet E, Traver S, Monnet Y, Perrin A, Mandjee N, Hild A, Hirsch EC, Zheng TS, Hunot S. Tumor necrosis factor-like weak inducer of apoptosis induces astrocyte proliferation through the activation of transforming-growth factor-alpha/epidermal growth factor receptor signaling pathway. Mol. Pharmacol, 2012; 82: 948-57.

Rucinski M, Ziolkowska A, Szyszka M, Malendowicz LK. Precerebellin-related genes and precerebellin 1 peptide in the adrenal gland of the rat: expression pattern, localization, developmental regulation and effects on corticosteroidogenesis. Int. J Mol. Med, 2009; 23: 363-71.

Ruland J, Duncan GS, Elia A, del BB, I, Nguyen L, Plyte S, Millar DG, Bouchard D, Wakeham A, Ohashi PS, Mak TW. Bcl10 is a positive regulator of antigen receptor-induced activation of NF-kappaB and neural tube closure. Cell, 2001; 104: 33-42.

Salehi AH, Roux PP, Kubu CJ, Zeindler C, Bhakar A, Tannis LL, Verdi JM, Barker PA. NRAGE, a novel MAGE protein, interacts with the p75 neurotrophin receptor and facilitates nerve growth factor-dependent apoptosis. Neuron, 2000; 27: 279-88.

Sama DM, Mohmmad AH, Furman JL, Artiushin IA, Szymkowski DE, Scheff SW, Norris CM. Inhibition of soluble tumor necrosis factor ameliorates synaptic alterations and Ca2+ dysregulation in aged rats. PLoS. One., 2012; 7: e38170.

Samuels-Lev Y, O'Connor DJ, Bergamaschi D, Trigiante G, Hsieh JK, Zhong S, Campargue I, Naumovski L, Crook T, Lu X. ASPP proteins specifically stimulate the apoptotic function of p53. Mol. Cell, 2001; 8: 781-94.

Sarkisian MR, Siebzehnrubl D. Abnormal levels of Gadd45alpha in developing neocortex impair neurite outgrowth. PLoS. One., 2012; 7: e44207.

Sarras H, Alizadeh AS, McPherson JP. In search of a function for BCLAF1. ScientificWorldJournal., 2010; 10: 1450-61.

Sayan AE, Sayan BS, Gogvadze V, Dinsdale D, Nyman U, Hansen TM, Zhivotovsky B, Cohen GM, Knight RA, Melino G. P73 and caspase-cleaved p73 fragments localize to mitochondria and augment TRAIL-induced apoptosis. Oncogene, 2008; 27: 4363-72.

Scheinfeld MH, Ghersi E, Davies P, D'Adamio L. Amyloid beta protein precursor is phosphorylated by JNK-1 independent of, yet facilitated by, JNK-interacting protein (JIP)-1. J Biol. Chem., 2003; 278: 42058-63.

Schmidt-Kastner R, guirre-Chen C, Kietzmann T, Saul I, Busto R, Ginsberg MD. Nuclear localization of the hypoxia-regulated pro-apoptotic protein BNIP3 after global brain ischemia in the rat hippocampus. Brain Res., 2004; 1001: 133-42.

Schneider TJ, Fischer GM, Donohoe TJ, Colarusso TP, Rothstein TL. A novel gene coding for a Fas apoptosis inhibitory molecule (FAIM) isolated from inducibly Fas-resistant B lymphocytes. J Exp Med, 1999; 189: 949-56.

Sculley DG, Dawson PA, Emmerson BT, Gordon RB. A review of the molecular basis of hypoxanthine-guanine phosphoribosyltransferase (HPRT) deficiency. Hum. Genet., 1992; 90: 195-207.

Shaw PJ, Barr MJ, Lukens JR, McGargill MA, Chi H, Mak TW, Kanneganti TD. Signaling via the RIP2 adaptor protein in central nervous system-infiltrating dendritic cells promotes inflammation and autoimmunity. Immunity., 2011; 34: 75-84.

Shepherd AJ, Loo L, Gupte RP, Mickle AD, Mohapatra DP. Distinct modifications in Kv2.1 channel via chemokine receptor CXCR4 regulate neuronal survival-death dynamics. J Neurosci, 2012; 32: 17725-39.

Shi H, Sun BL, Zhang J, Lu S, Zhang P, Wang H, Yu Q, Stetler RA, Vosler PS, Chen J, Gao Y. miR-15b suppression of Bcl-2 contributes to cerebral ischemic injury and is reversed by sevoflurane preconditioning. CNS. Neurol. Disord. Drug Targets, 2013; 12: 381-91.

Shim SY, Kim HS, Kim EK, Choi JH. Expression of peroxiredoxin 1, 2, and 6 in the rat brain during perinatal development and in response to dexamethasone. Free Radic. Res., 2012; 46: 231-9.

Shimoke K, Amano H, Kishi S, Uchida H, Kudo M, Ikeuchi T. Nerve growth factor attenuates endoplasmic reticulum stress-mediated apoptosis via suppression of caspase-12 activity. J Biochem., 2004; 135: 439-46.

Shinoda S, Skradski SL, Araki T, Schindler CK, Meller R, Lan JQ, Taki W, Simon RP, Henshall DC. Formation of a tumour necrosis factor receptor 1 molecular scaffolding complex and activation of apoptosis signal-regulating kinase 1 during seizure-induced neuronal death. Eur. J Neurosci, 2003; 17: 2065-76.

Shioda N, Han F, Moriguchi S, Fukunaga K. Constitutively active calcineurin mediates delayed neuronal death through Fas-ligand expression via activation of NFAT and FKHR transcriptional activities in mouse brain ischemia. J Neurochem., 2007; 102: 1506-17.

Sifringer M, Stefovska V, Endesfelder S, Stahel PF, Genz K, Dzietko M, Ikonomidou C, Felderhoff-Mueser U. Activation of caspase-1 dependent interleukins in developmental brain trauma. Neurobiol. Dis., 2007; 25: 614-22.

Slane McQuade JM, Vorhees CV, Xu M, Zhang J. DNA fragmentation factor 45 knockout mice exhibit longer memory retention in the novel object recognition task compared to wild-type mice. Physiol Behav., 2002; 76: 315-20.

Slotkin TA, Seidler FJ, Fumagalli F. Targeting of neurotrophic factors, their receptors, and signaling pathways in the developmental neurotoxicity of organophosphates in vivo and in vitro. Brain Res. Bull., 2008; 76: 424-38.

Smith AK, Conneely KN, Kilaru V, Mercer KB, Weiss TE, Bradley B, Tang Y, Gillespie CF, Cubells JF, Ressler KJ. Differential immune system DNA methylation and cytokine regulation in post-traumatic stress disorder. Am. J Med Genet. B Neuropsychiatr. Genet., 2011; 156B: 700-8.

So T, Croft M. Regulation of PI-3-Kinase and Akt Signaling in T Lymphocytes and Other Cells by TNFR Family Molecules. Front Immunol., 2013; 4: 139.

Soane L, Siegel ZT, Schuh RA, Fiskum G. Postnatal developmental regulation of Bcl-2 family proteins in brain mitochondria. J Neurosci Res., 2008; 86: 1267-76.

Spulber S, Mateos L, Oprica M, Cedazo-Minguez A, Bartfai T, Winblad B, Schultzberg M. Impaired long term memory consolidation in transgenic mice overexpressing the human soluble form of IL-1ra in the brain. J Neuroimmunol., 2009; 208: 46-53.

Strausberg RL, Feingold EA, Grouse LH, Derge JG, Klausner RD, Collins FS, Wagner L, Shenmen CM, Schuler GD, Altschul SF, Zeeberg B, Buetow KH, Schaefer CF, Bhat NK, Hopkins RF, Jordan H, Moore T, Max SI, Wang J, Hsieh F, Diatchenko L, Marusina K, Farmer AA, Rubin GM, Hong L, Stapleton M, Soares MB, Bonaldo MF, Casavant TL, Scheetz TE, Brownstein MJ, Usdin TB, Toshiyuki S, Carninci P, Prange C, Raha SS, Loquellano NA, Peters GJ, Abramson RD, Mullahy SJ, Bosak SA, McEwan PJ, McKernan KJ, Malek JA, Gunaratne PH, Richards S, Worley KC, Hale S, Garcia AM, Gay LJ, Hulyk SW, Villalon DK, Muzny DM, Sodergren EJ, Lu X, Gibbs RA, Fahey J, Helton E, Ketteman M, Madan A, Rodrigues S, Sanchez A, Whiting M, Madan A, Young AC, Shevchenko Y, Bouffard GG, Blakesley RW, Touchman JW, Green ED, Dickson MC, Rodriguez AC, Grimwood J, Schmutz J, Myers RM, Butterfield YS, Krzywinski MI, Skalska U, Smailus DE, Schnerch A, Schein JE, Jones SJ, Marra MA. Generation and initial analysis of more than 15,000 full-length human and mouse cDNA sequences. Proc Natl. Acad. Sci U. S. A, 2002; 99: 16899-903.

Sun D, Bullock MR, McGinn MJ, Zhou Z, Altememi N, Hagood S, Hamm R, Colello RJ. Basic fibroblast growth factor-enhanced neurogenesis contributes to cognitive recovery in rats following traumatic brain injury. Exp Neurol., 2009; 216: 56-65.

Sun D, Huang W, Hwang YY, Zhang Y, Zhang Q, Li MD. Regulation by nicotine of Gpr51 and Ntrk2 expression in various rat brain regions. Neuropsychopharmacology, 2007; 32: 110-6.

Sun H, Huang Y, Yu X, Li Y, Yang J, Li R, Deng Y, Zhao G. Peroxisome proliferator-activated receptor gamma agonist, rosiglitazone, suppresses CD40 expression and attenuates inflammatory responses after lithium pilocarpine-induced status epilepticus in rats. Int. J Dev. Neurosci, 2008; 26: 505-15.

Sun Y, Lee JH, Kim NH, Lee CW, Kim MJ, Kim SH, Huh SO. Lysophosphatidylcholine-induced apoptosis in H19-7 hippocampal progenitor cells is enhanced by the upregulation of Fas Ligand. Biochim. Biophys. Acta, 2009; 1791: 61-8.

Syed AU, Koide M, Braas KM, May V, Wellman GC. Pituitary adenylate cyclase-activating polypeptide (PACAP) potently dilates middle meningeal arteries: implications for migraine. J Mol. Neurosci, 2012; 48: 574-83.

Tamm C, Zhivotovsky B, Ceccatelli S. Caspase-2 activation in neural stem cells undergoing oxidative stress-induced apoptosis. Apoptosis., 2008; 13: 354-63.

Tan SC, Carr CA, Yeoh KK, Schofield CJ, Davies KE, Clarke K. Identification of valid housekeeping genes for quantitative RT-PCR analysis of cardiosphere-derived cells preconditioned under hypoxia or with prolyl-4-hydroxylase inhibitors. Mol. Biol. Rep., 2012; 39: 4857-67.

Tanriverdi F, De BA, Bizzarro A, Sinisi AA, Bellastella G, Pane E, Bellastella A, Unluhizarci K, Selcuklu A, Casanueva FF, Kelestimur F. Antipituitary antibodies after traumatic brain injury: is head trauma-induced pituitary dysfunction associated with autoimmunity? Eur. J Endocrinol., 2008; 159: 7-13.

Tewari M, Yu M, Ross B, Dean C, Giordano A, Rubin R. AAC-11, a novel cDNA that inhibits apoptosis after growth factor withdrawal. Cancer Res., 1997; 57: 4063-9.

Thangavel R, Stolmeier D, Yang X, Anantharam P, Zaheer A. Expression of glia maturation factor in neuropathological lesions of Alzheimer's disease. Neuropathol. Appl. Neurobiol., 2012; 38: 572-81.

Tiwari S, Siddiqi S, Siddiqi SA. CideB protein is required for the biogenesis of very low density lipoprotein (VLDL) transport vesicle. J Biol. Chem., 2013; 288: 5157-65.

Tofighi R, Joseph B, Xia S, Xu ZQ, Hamberger B, Hokfelt T, Ceccatelli S. Galanin decreases proliferation of PC12 cells and induces apoptosis via its subtype 2 receptor (GalR2). Proc Natl. Acad. Sci U. S. A, 2008; 105: 2717-22.

Tsuiki H, Asai K, Yamamoto M, Fujita K, Inoue Y, Kawai Y, Tada T, Moriyama A, Wada Y, Kato T. Cloning of a rat glia maturation factor-gamma (rGMFG) cDNA and expression of its mRNA and protein in rat organs. J Biochem., 2000; 127: 517-23.

Upadhyay V, Fu YX. Lymphotoxin signalling in immune homeostasis and the control of microorganisms. Nat Rev. Immunol., 2013; 13: 270-9.

Uribe V, Wong BK, Graham RK, Cusack CL, Skotte NH, Pouladi MA, Xie Y, Feinberg K, Ou Y, Ouyang Y, Deng Y, Franciosi S, Bissada N, Spreeuw A, Zhang W, Ehrnhoefer DE, Vaid K, Miller FD, Deshmukh M, Howland D, Hayden MR. Rescue from excitotoxicity and axonal degeneration accompanied by age-dependent behavioral and neuroanatomical alterations in caspase-6-deficient mice. Hum. Mol. Genet., 2012; 21: 1954-67.

Vivar R, Humeres C, Ayala P, Olmedo I, Catalan M, Garcia L, Lavandero S, az-Araya G. TGF-beta1 prevents simulated ischemia/reperfusion-induced cardiac fibroblast apoptosis by activation of both canonical and non-canonical signaling pathways. Biochim. Biophys. Acta, 2013; 1832: 754-62.

Wan C, Jiang J, Mao H, Cao J, Wu X, Cui G. Involvement of Upregulated P53-Induced Death Domain Protein (PIDD) in Neuronal Apoptosis after Rat Traumatic Brain Injury. J Mol. Neurosci, 2013.

Wang G, Qian P, Xu Z, Zhang J, Wang Y, Cheng S, Cai W, Qian G, Wang C, Decoster MA. Regulatory effects of the JAK3/STAT1 pathway on the release of secreted phospholipase A(2)-IIA in microvascular endothelial cells of the injured brain. J Neuroinflammation, 2012; 9: 170.

Wang HF, Shih YT, Chen CY, Chao HW, Lee MJ, Hsueh YP. Valosin-containing protein and neurofibromin interact to regulate dendritic spine density. J Clin. Invest, 2011; 121: 4820-37.

Wang Q, Li A, Wang H, Wang J. Knockdown of apoptosis repressor with caspase recruitment domain (ARC) increases the sensitivity of human glioma cell line U251MG to VM-26. Int. J Clin. Exp Pathol., 2012; 5: 555-61.

Wang Y, Koroleva EP, Kruglov AA, Kuprash DV, Nedospasov SA, Fu YX, Tumanov AV. Lymphotoxin beta receptor signaling in intestinal epithelial cells orchestrates innate immune responses against mucosal bacterial infection. Immunity., 2010; 32: 403-13.

Wang Y, Tang X, Yu B, Gu Y, Yuan Y, Yao D, Ding F, Gu X. Gene network revealed involvements of Birc2, Birc3 and Tnfrsf1a in anti-apoptosis of injured peripheral nerves. PLoS. One., 2012; 7: e43436.

Waters RJ, Murray GD, Teasdale GM, Stewart J, Day I, Lee RJ, Nicoll JA. Cytokine Gene Polymorphisms and Outcome after Traumatic Brain Injury. J Neurotrauma, 2013.

Wei XH, Na XD, Liao GJ, Chen QY, Cui Y, Chen FY, Li YY, Zang Y, Liu XG. The up-regulation of IL-6 in DRG and spinal dorsal horn contributes to neuropathic pain following L5 ventral root transection. Exp Neurol., 2013; 241: 159-68.

Weinberg MS, Blake BL, McCown TJ. Opposing actions of hippocampus TNFalpha receptors on limbic seizure susceptibility. Exp Neurol., 2013; 247: 429-37.

Wong LF, Ralph GS, Walmsley LE, Bienemann AS, Parham S, Kingsman SM, Uney JB, Mazarakis ND. Lentiviral-mediated delivery of Bcl-2 or GDNF protects against excitotoxicity in the rat hippocampus. Mol. Ther, 2005; 11: 89-95.

Woodbury ME, Ikezu T. Fibroblast Growth Factor-2 Signaling in Neurogenesis and Neurodegeneration. J Neuroimmune. Pharmacol, 2013.

Xiao Q, Ford AL, Xu J, Yan P, Lee KY, Gonzales E, West T, Holtzman DM, Lee JM. Bcl-x pre-mRNA splicing regulates brain injury after neonatal hypoxia-ischemia. J Neurosci, 2012; 32: 13587-96.

Xu C, Lv L, Zheng G, Li B, Gao L, Sun Y. Neuregulin1beta1 protects oligodendrocyte progenitor cells from oxygen glucose deprivation injury induced apoptosis via ErbB4-dependent activation of PI3-kinase/Akt. Brain Res., 2012; 1467: 104-12.

Xu T, Wang X, Cao M, Wu X, Yan Y, Fu H, Zhao W, Gong P, Ke K, Gu X. Increased expression of BAG-1 in rat brain cortex after traumatic brain injury. J Mol. Histol., 2012; 43: 335-42.

Yahya A, Xiao C, Chance WT, Sheriff S. Up-regulation of neuropeptide Y Y4 receptor mRNA expression in the brainstem of refed rats following 48 h of food deprivation: effect of leptin. Peptides, 2006; 27: 2731-7.

Yang J, You Z, Kim HH, Hwang SK, Khuman J, Guo S, Lo EH, Whalen MJ. Genetic analysis of the role of tumor necrosis factor receptors in functional outcome after traumatic brain injury in mice. J Neurotrauma, 2010; 27: 1037-46.

Yang Y, Xie Y, Chai H, Fan M, Liu S, Liu H, Bruce I, Wu W. Microarray analysis of gene expression patterns in adult spinal motoneurons after different types of axonal injuries. Brain Res., 2006; 1075: 1-12.

Yi JH, Park SW, Brooks N, Lang BT, Vemuganti R. PPARgamma agonist rosiglitazone is neuroprotective after traumatic brain injury via anti-inflammatory and anti-oxidative mechanisms. Brain Res, 2008; 1244: 164-72.

Yin XH, Yan JZ, Hou XY, Wu SL, Zhang GY. Neuroprotection of S-nitrosoglutathione against ischemic injury by down-regulating Fas S-nitrosylation and downstream signaling. Neuroscience, 2013; 248C: 290-8.

Yin XM. Bid, a BH3-only multi-functional molecule, is at the cross road of life and death. Gene, 2006; 369: 7-19.

Yoo YM, Lee CJ, Kim YJ. Exogenous GDNF increases the migration of the neural stem cells with no protection against kainic acid-induced excitotoxic cell death in rats. Brain Res., 2012; 1486: 27-38.

Yung LM, Wei Y, Qin T, Wang Y, Smith CD, Waeber C. Sphingosine kinase 2 mediates cerebral preconditioning and protects the mouse brain against ischemic injury. Stroke, 2012; 43: 199-204.

Zhang M, Xu G, Liu W, Ni Y, Zhou W. Role of fractalkine/CX3CR1 interaction in light-induced photoreceptor degeneration through regulating retinal microglial activation and migration. PLoS. One., 2012; 7: e35446.

Zhou YT, Guy GR, Low BC. BNIP-2 induces cell elongation and membrane protrusions by interacting with Cdc42 via a unique Cdc42-binding motif within its BNIP-2 and Cdc42GAP homology domain. Exp Cell Res., 2005; 303: 263-74.

Zou XJ, Yang L, Yao SL. Endoplasmic reticulum stress and C/EBP homologous protein-induced Bax translocation are involved in angiotensin II-induced apoptosis in cultured neonatal rat cardiomyocytes. Exp Biol. Med (Maywood. ), 2012; 237: 1341-9.
